# The skin dose of pelvic radiographs since 1896

**DOI:** 10.1186/s13244-019-0710-1

**Published:** 2019-03-29

**Authors:** Gerrit J. Kemerink, Gerhard Kütterer, Pierre J. Kicken, Jos M. A. van Engelshoven, Kees J. Simon, Joachim E. Wildberger

**Affiliations:** Department of Radiology and Nuclear Medicine, Maastricht University Medical Center, P. Debijelaan 25, 6229 HX Maastricht, The Netherlands

**Keywords:** History radiology, First X-ray systems, Early dosimetry, Pelvic radiography, Dose reconstruction

## Abstract

**Objectives:**

To derive conversions of antiquated exposure data into modern equivalents and to apply these in the assessment of the skin dose of pelvic radiographs since 1896.

**Methods:**

The literature 1896–2018 was searched for implicit and explicit dose information. The early implicit dose data contained now obsolete descriptions of radiation quality and quantity for long since disappeared X-ray systems of limited efficiency. Converting the old information into modern specifications was achieved using contemporary data and computer simulations. Final dose calculations were done with modern software. Explicit radiation doses of later date reported in old quantities and units were adapted according to current recommendations.

**Results:**

For the period before 1927 conversion algorithms for spark gap data and penetrometer hardnesses to high voltage could be derived. Electrical and X-ray efficiencies of several old röntgen systems were determined. Together they allowed reconstruction of 53 doses. After 1927 doses were generally explicitly specified; 114 were retrieved. Although an enormous spread was observed, the average skin dose was reduced by a factor of about 400.

**Conclusions:**

Antiquated exposure data were successfully used for dose reconstruction. Extreme dose variability was a constant. Efforts to cut down doses were effective as skin doses went down from sub-erythema values to about one milligray.

**Electronic supplementary material:**

The online version of this article (10.1186/s13244-019-0710-1) contains supplementary material, which is available to authorized users.

## Key points

- Implicit, antiquated dose information from 1896 to 1926 was successfully used in dose reconstruction
- Explicit doses appeared after 1927, but were up to recent times in now outdated quantities and units
- During 1896–2018 the dose of pelvic radiographs was reduced by a factor of about 400
- At all times an extremely large spread in reconstructed and retrieved doses was observed
- Dose reconstruction methods developed in this study might be applied to radiography or fluoroscopy of other body parts

## Introduction

Investigating the origin of gonad shielding, we searched for information on radiation doses around 1900. It turned out that such data are effectively missing, probably because dosimetry, as we know it today, was only introduced around 1927. To our knowledge only Kotre et al. tried to estimate radiation doses before this time in a quantitative way, actually for patients and staff in Newcastle between 1899 and 1902 [1]. We decided to address this issue on a larger scale, taking the entrance dose required for a radiograph in the pelvic area as the main subject of our investigation. In this study information from the literature was combined with results from computer simulations.

With hindsight the dosimetric problems of users of X-ray systems directly after 1895 are easy to see. Their theoretical framework was incomplete, they lacked the tools to measure all essential system characteristics and their X-ray tubes were inconstant in their functioning. It explains why users gave no (or at best incomplete) descriptions of X-ray exposures. Dose reconstruction for this period is therefore only possible if missing information can be deduced from other sources. Between 1900 and 1910 descriptions of radiographic exposures improved from deficient to complete. However, until 1927 information on high voltage, radiation hardness and dose was always in antiquated form. The transformation of these old data to modern quantities, including the assessment of the röntgen systems involved, is at the core of this study. To put the newly estimated doses from early radiology into perspective, explicit dose data from 1927 through 2018 were retrieved and converted into current equivalents if necessary.

To limit the size of the text, most background information and results from simulations were moved to Additional file 1, often already referred to in the header of a section. In principle the main text can be read without consulting these more technical addenda.

## Materials and methods

When dealing with X-ray exposure of the skin, most investigators today use the “entrance-surface air kerma including backscatter” (ESAK; or simply “dose measured in air on the skin”). However, kerma free in air (KfiA) and the absorbed dose in superficial tissue (skin absorbed dose, SAD) are used as well. We follow the majority and current recommendations [2] and use the ESAK, but conversions are easy on the basis of:

$$ \mathrm{SAD}={\mathrm{f}}^{\ast }\ \mathrm{ESAK}={\mathrm{f}}^{\ast }\ {\mathrm{BSF}}^{\ast }\ \mathrm{KfiA}, $$

where the KfiA is measured at the position of the skin but with the patient removed, f the ratio of mass energy absorption coefficients in tissue and air (about 1.05 for our purpose [3]), and BSF the back scatter factor that accounts for the dose contributed by radiation scattered elsewhere.

Dose information on pelvic radiography for the period 1896–2018 was collected from scientific journals, books and documentation from manufacturers of X-ray equipment in English, German and French. To reduce selection bias, any consulted publication containing adequate dose information was included. If for some parameter a range was given, we took the middle of that range. When in later years the dose quantity (KfiA, ESAK, SAD) was not specified, we would assume what appeared most probable. Likewise, when in later implicit dose data the filtration was missing, we chose a value typical for the time.

### Reconstruction of doses before 1927 – the difficult but interesting period

#### Chromoradiometers (Additional file 1: ES I)

Between 1902 and 1907 several dosimeters were introduced that relied on a chemical or physical change of some substance under the influence of X-rays (e.g. color change of a salt or sensitization of a photographic emulsion). These so-called chromoradiometers were calibrated with a dose corresponding to a certain radiation induced affection of the skin. Relevant to us is the epilation or “pastille” dose amounting to about 3.9 Gy [4]. With this calibration old readings for clinical exposures can easily be converted into ESAK. Unfortunately radiometer data were scarce for diagnostic radiology, so our dose reconstruction had mainly to rely on electrical, geometrical and possibly other information that characterizes an X-ray exposure. Dose estimation along this line we call the “computational approach”.

#### Computational approach

This dose reconstruction was divided into four steps:

I. Identifying the X-rays systems of early radiography associated with useful exposure data.
II. Determining the parameters relevant for the calculation of the dose of a radiograph, and if these were specified in antiquated form, establishing the conversion into modern quantities and units.
III. Finding the output of the ancient (pulsed) X-ray systems relative to that of a direct current (DC) system. This is necessary because modern software programs required for the calculation of dose and the simulation of X-ray spectra are available for DC systems only. From such programs we selected the powerful and user friendly SpekCalc [5].
IV. Retrieving clinical exposure data and computing skin doses.

Steps I-III are presented here under “Materials and Methods”, step IV in the “Results” section.

In our computations we relied on the NIST attenuation data [6, 7] and the “anti-plotter” program Graph Grabber [8]. Additional information on software and other resources is given in Additional file 1: ES II.

**Step I computational approach. Relevant ancient X-ray systems (Additional file** 1: **ES III and ES IV)**

Exposure information must contain the type of X-ray system used as it determines both the electrical and X-ray efficiency. The types we encountered with complete further information, were:

- A. Inductor coil with interrupter and ion tube (1896+)
- B. Transformer, mostly with mechanical rectifier (‘Snook’, 1907+), and ion tube
- C1. Transformer with mechanical rectifier (Snook) and Coolidge tube (1913+)
- C2. Transformer and self-rectifying Coolidge tube (1913+), or transformer with vacuum tube rectifiers and Coolidge tube (1926+)
- D. Inductor and Coolidge tube (1913+)

All systems mentioned applied pulsed voltage to the X-ray tube. We will see that C1 and C2 gave similar output and radiation quality. Additional characteristics of these systems can be found in Additional file 1: ES III; for thermal properties of some early ion tubes see Additional file 1: ES IV.

**Step II computational approach. The parameters that are required**

For a given type of X-ray system the additional parameters required to characterize the making of a radiograph are summarized in Table 1. The left column shows the information needed, the right column the available information from which these parameters might be derived.

**Table 1:** Parameters relevant in characterizing historical radiographs

Modern parameter	Old information (pre-1927)
Peak high-voltage (kVp)	Equivalent spark gap on functioning tube
	Hardness X-rays according to penetrometer or half value layer in water
	Electrical information on primary and secondary circuit
Tube current (mA)^a^	Average current, after 1904 often specified
	Electrical information on primary and secondary circuit
Exposure time (min, s)^a^	Explicitly specified duration of exposure
Target (anode) material	Explicit or implicit: before 1912 platinum (Pt), then replacement by tungsten (W)
Filtration	Implicit: no filter added, i.e. tube envelope only. Later sometimes specified filter
Exposure geometry	Focus-plate or focus-patient distance, field size, type of study
Backscatter factor	kVp-info, filtration, object thickness, expo-geometry. Explicit BSF seldom & later

^a^Often only product of tube current and exposure time was reported (unit: mA.min or mAs)

We now consider the parameters individually.

*– kVp – from spark gap (Additional file* *1* *: ES V)*

The high voltage (HV) over the X-ray tube is the main determinant of the radiation quality (penetrating power) and an important factor in the X-ray output rate (intensity). In early radiology it proved impossible to measure a good proxy for the pulsed voltages commonly used. X-ray users resorted therefore to measuring quantities that were related to it. Best known is the spark gap, where this name can both refer to the length a spark could bridge and the hardware for its measurement (two pins, a pin and plate, or two spheres).

**Fig. 1:**
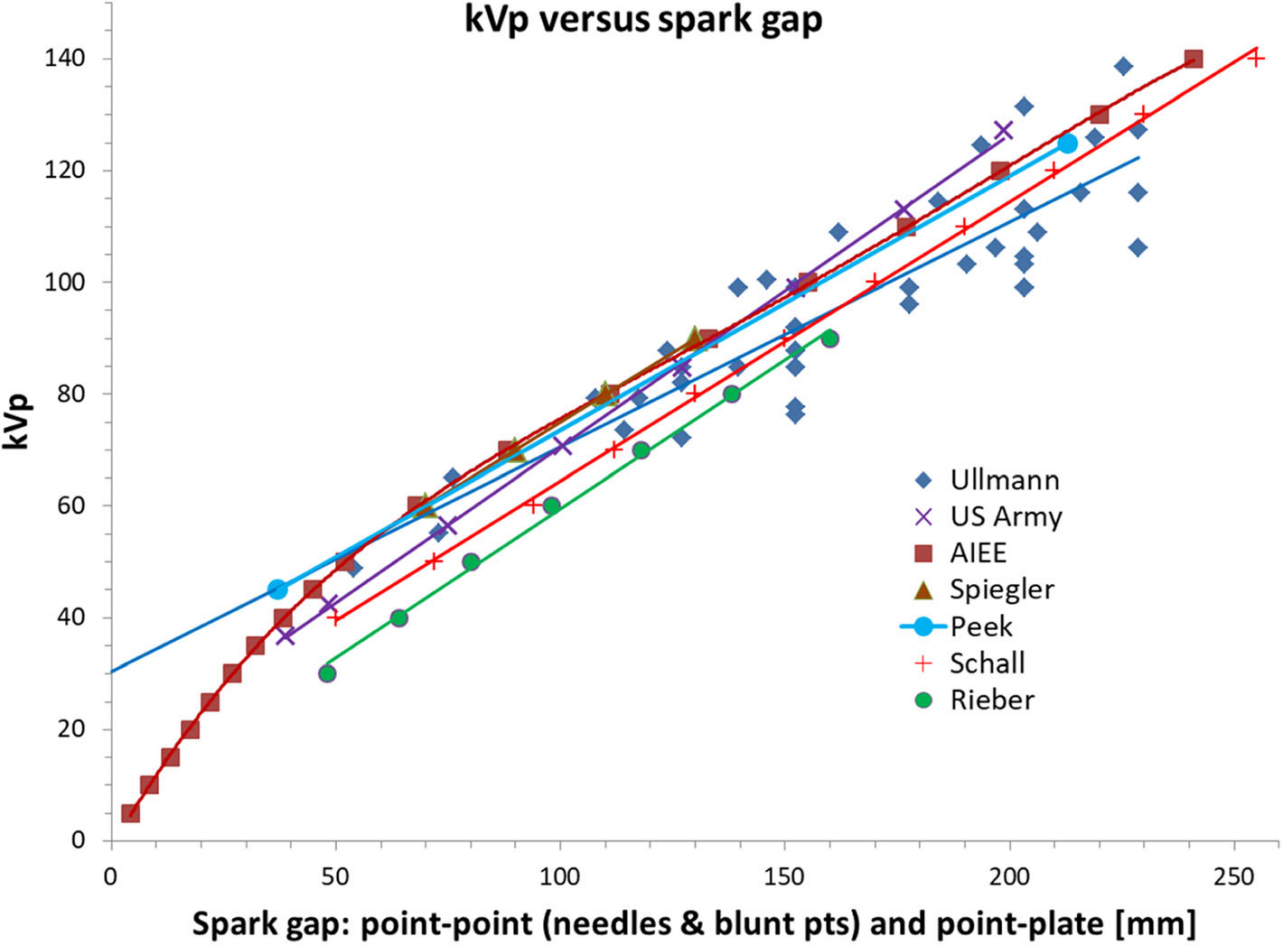
Relation between spark gap length and kVp according to various authors [9–15]

We found several sources giving a relation between spark gap and high voltage (Fig. 1) [9–15]. Linear or polynomial fits to the data points were made. Each fit was separately used in converting a spark gap length reported for a clinical radiograph into a kVp-value and then the average of all results was taken.

Measuring the spark gap was simple, but it appeared that the quality of the X-ray output could vary between systems even if the spark gaps were the same. Possible causes of these disparities are discussed in Additional file 1: ES V.

*– kVp – from penetrometer hardness (Additional file* *1* *: ES VI)*

**Fig. 2:**
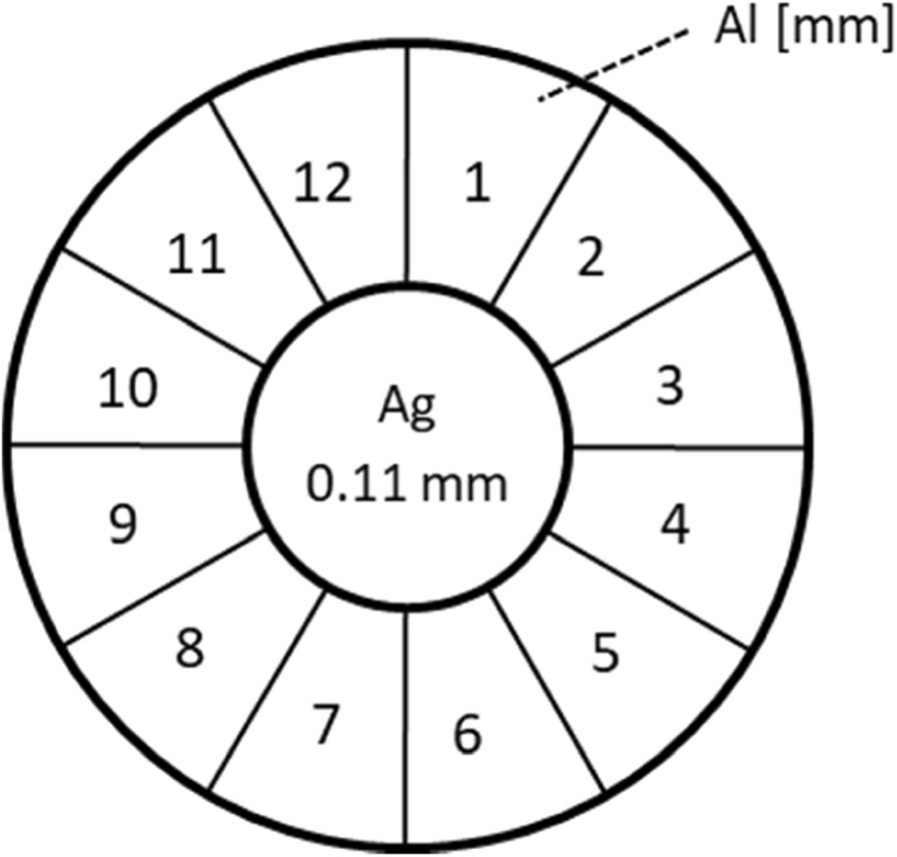
Top view of Benoist hardness meter [16]. In the center a foil of silver (Ag, 0.11 mm thickness), surrounded by a circular staircase of aluminum (Al) with step thicknesses increasing clockwise from 1 to 12 mm. This penetrometer was held in the X-ray beam in front of a fluoroscopic screen and the Benoist hardness was the thickness of the Al-step that showed the same intensity as that of the Ag-foil in the middle. Evaluation was also done with a photographic plate [16]

**Fig. 3:**
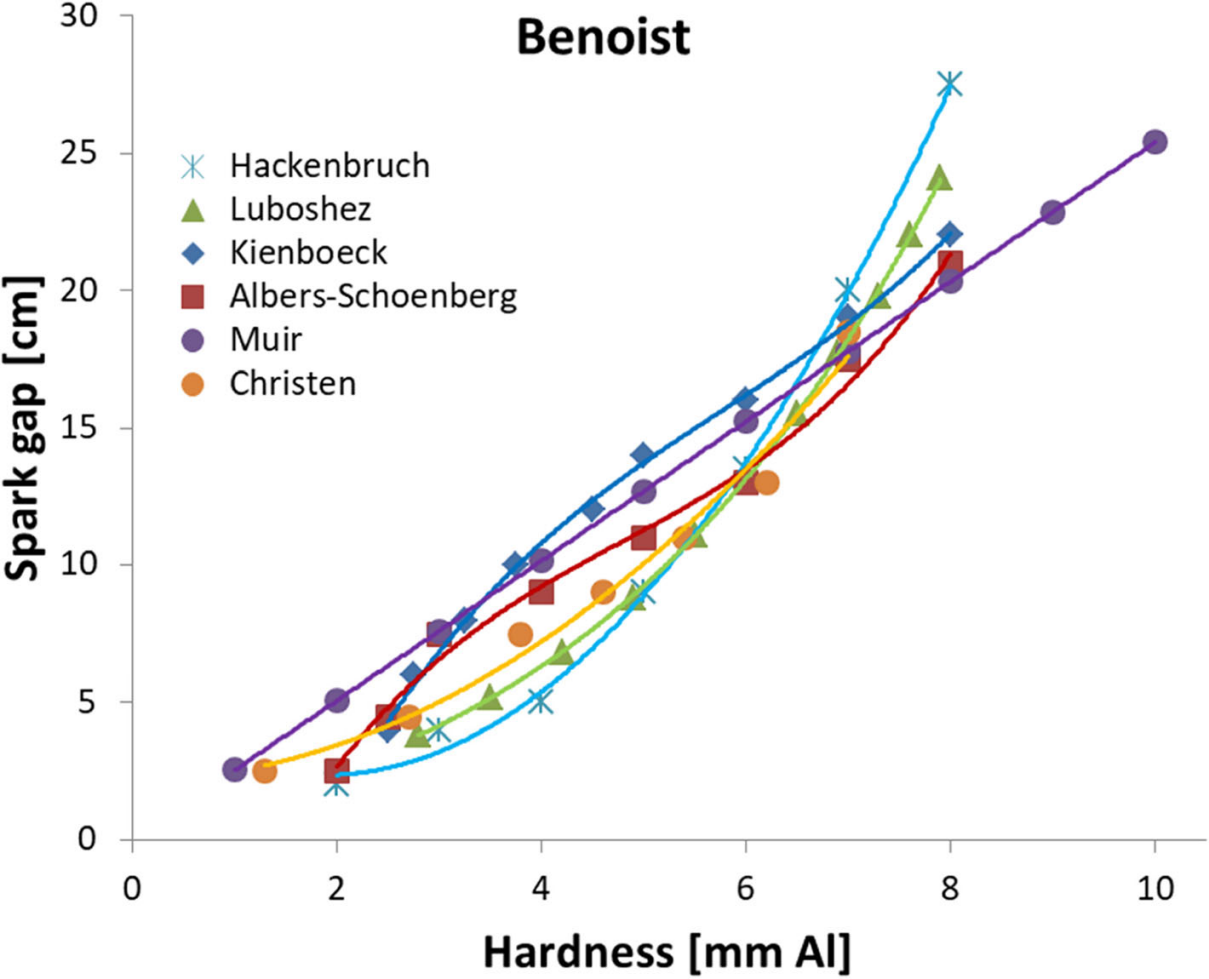
Spark gap length as a function of the Benoist hardness. Most often the letter B was used, only followed by a number, thus without the unit “mm Al” [17–22]

Probably because of the limitations of the spark gap in assessing radiation quality, Benoist [16] designed in 1902 a so called penetrometer that gave a reading (“hardness”) that was affected by the whole X-ray spectrum and not only by its maximum energy represented by the kVp. Figure 2 illustrates the construction of the device. Reported relations between the hardness and the corresponding equivalent spark gap are shown in Fig. 3 [17–22]. The data were fitted with polynomials and the fitted functions hereafter used in the conversion of a clinical hardness into a spark gap. The latter was then converted into a kVp.

Several other penetrometers came into use as well. We consider only the devices encountered in descriptions of exposures, which were in addition to Benoist’s those according Benoist-Walter, Wehnelt and Walter. For the latter three relations between hardness and spark gap are shown in Additional file 1: ES VI. SpekCalc simulations of the Benoist and Wehnelt penetrometers are also presented there.

Christen provided a major contribution to dosimetry in 1913 with his monograph “Messung und Dosierung der Röntgenstrahlen” [23]. He advocated the use of the half value layer (HVL) of X-rays in water for quantifying their hardness. However, we found virtually no HVL data for clinical radiography, but data on the relation between hardness and HVL were relatively numerous; these are also shown in Additional file 1: ES VI.

*– kVp – from electrical information (Additional file* *1* *: ES VII)*

In the first two decades of radiology, exposure data sometimes pertained (partially) to the primary low voltage circuit of the inductor. If the input power (P_prim_) and the system’s electrical transformation efficiency α are known (Table 2), the electrical power available to the secondary high voltage load can be calculated according to P_sec_ = α * P_prim_.

**Table 2:** Electrical transformation efficiency (α) of inductor with various interrupters (Additional file 1: ES VII)

	Coil itself	mechanical	mercury	Wehnelt
Transformation efficiency α	0.60	0.45	0.50	0.17
Estimated variability	0.20	0.25	0.20	0.07

α is power available to the load of the secondary circuit divided by that spent in the primary circuit of the inductor (including interrupter)

Incidentally, for the soft radiation of early radiology simulations show, for a fixed secondary power, that the kVp and tube current can be varied within large margins without changing the KfiA (see Additional file 1: Figure ES13). This means that any (reasonable) kVp can be chosen to calculate the tube current from P_sec_. However, if given, the reported kVp was used.

*– mAs or mA.min*

Contrary to the high voltage over the tube, the current through the tube could relatively easily be measured with a moving coil milliampere meter, introduced in 1904 by Gaiffe [24]. Exposure time was initially measured and controlled manually and later (electro-) mechanically.

*– Anode material – Pt, W (Additional file* *1* *: ES VIII)*

The first dose data pertained to systems with ion tubes having a platinum (Pt) anode (called the anti-cathode in tubes with three electrodes). After 1912 tungsten (W) started to replace platinum. Coolidge also used W for the anode in his hot cathode tube from 1913 [25]. Because the X-ray output is approximately proportional to the atomic number Z, output correction for instance from W (as assumed in SpekCalc) to the actually used Pt is easy: multiplication of the W-output by Z_Pt_/Z_W_ = 78/74 = 1.054 (the anode factor). The anode angle in ion tubes, both for gas and Coolidge tubes, was typically 45 degrees until the introduction of the line (“Goetze”) focus in 1922 [26].

*– Filtration – glass envelope of tube, additional filter, air (Additional file* *1* *: ES IX)*

Several early authors gave information on the thickness of the tube’s glass wall in the exit region of the X-rays; they all agree on values between 0.2 and 1.1 mm, the actual value being related to the diameter of the tube. In our calculations we assumed a wall thickness of 0.85 mm glass, except for two small ion tubes from 1896, for which we took 0.6 mm (Additional file 1: ES IX). For a very long time walls remained thin and until the twenties generally no additional filter was applied in radiography.

*– Exposure geometry*

In descriptions of the geometry used in making a pelvic radiograph often only the distance between the X-ray focus and photographic plate was specified. In such cases we assumed a patient thickness of 20 cm and a distance of 1 cm between patient and plate. When in later years (1920+) the use of a Bucky-Potter grid was reported the latter distance was set to 5 cm.

*– Backscattered X-rays*

The backscatter factor (BSF) mainly depends on high voltage, X-ray filtration, field size and object thickness, and was calculated from data published by Harrison [27] and the NCRP Report 68 [28]; most published BSF-values apply to too heavily filtered X-rays. For pelvic radiography, BSF-values varied from 1.11 to 1.17 (after 1927 up to 1.49).

**Step III computational approach. Estimation of output of a pulsed system from that of a DC system (Additional file** 1: **ES X)**

After Step II we have, for a given röntgen system, information on all exposure parameters specified in Table 1. For dose calculation using a “DC program” we now have to relate the output of a tube excited with a pulsed voltage to one driven by a DC power supply. One way of accomplishing this is by determining an “X-ray efficiency factor” (ε) with which the output of a DC system, using the kVp of the pulsed system, must be scaled to get the output of the pulsed system. The factor ε we are looking for depends on the waveforms of both the voltage and current exciting the X-ray tube, which vary with the type of the HV-generator and X-ray tube.

Many comparisons of X-ray output were published between 1920 and 1935, a smaller number before this time. We also simulated a few idealized voltage and current waveforms using SpekCalc. The combined result is shown in the column “Average” in Table 3. It turned out that the classical inductor ion tube system had on average an X-ray efficiency similar to that of a system using idealized sine-voltage, sine-current pulses.

**Table 3:** Different combinations of HV-generator and X-ray tube and their X-ray efficiency ε

#	HV-generator	X-ray tube	Ratio (KfiA pulsed)/(KfiA DC) (ε)		# sources
			Average	stand. dev.	
A	Inductor coil	Ion tube	0.69	0.14	12
B	Snook^a^	Ion tube	0.72	0.10	14
C1	Snook^a^	Coolidge tube	0.56	0.08	9
C2	Transformer^b^	Coolidge tube	0.56	0.08	13
D	Inductor coil	Coolidge tube	0.16–0.98^c^		

^a^Transformer with mechanical rectifier [55]. We use the name ‘Snook’ as a generic

^b^Transformer and self-rectifying Coolidge tube (one pulse), or transformer with vacuum tube rectifiers and Coolidge tube (one- and two-pulse)

^c^Tricky combination, coil could easily be overloaded (see Additional file 1: ES XI-D)

The KfiA of some old system, applying a pulsed high-voltage of kVp, can now be obtained by performing a SpekCalc (i.e. DC) simulation at the given value kVp and multiplying the KfiA from the simulation with the corresponding factor ε from Table 3. Multiplication with the BSF, and possibly the anode factor (Z_Pt_/Z_W_), yields the ESAK. An overview of all data used in determining ε is given in Additional file 1: ES XI, whereas Additional file 1: ESXII estimates the uncertainty in the reconstructed dose.

Our search for input for dose reconstruction revealed the impossibility of early radiology to make high quality anteroposterior radiographs of the (large) adult pelvis, mainly due to the loss of contrast by excessive scattered radiation. Good small-area X-ray radiographs of the hip, lumbar spine, kidney stones and abdomen were feasible though. These required more or less similar radiation quality and dose as the full pelvis. For the early years we used therefore information on all four types of images. Good radiographs of the adult pelvis could be made following the introduction of the moving Bucky-Potter anti-scatter grid after 1920 [29].

### Retrieval of “modern” dose data after 1927 – the easy period

Modern dose data became available after two important developments: 1. the wider use of the ionization chamber in the mid-nineteen twenties and 2. the international adoption of the roentgen (r) as the unit of exposure in 1928 [30]. In Germany a nearly identical unit had already been introduced in 1924 [31, 32], the German röntgen (R), which was a little larger, 1 R = 1.065 r. In 1953 and 1954 the units rad and rem were introduced for absorbed dose and dose equivalent, respectively [33, 34]. The quantity kerma followed in 1962 [35], as did in 1981 the new units gray (Gy, succeeded rad) and sievert (Sv, succeeded rem) [36]. The years mark the approximate time of appearance of these quantities and units in publications. Table 4 summarizes some of the older quantities and their units, and the factors for conversion into modern equivalents.

**Table 4:** Conversions of old quantities into ‘kerma in air’ or ‘absorbed dose’^a^

	Unit	Kerma in air [Gy]	Absorbed dose [Gy]
Exposure	r	0.00876	
Exposure	R	0.00934	
Absorbed dose	rad		0.01
Dose equivalent	rem		0.01

^a^Relations hold for diagnostic X-rays and, in case of exposure and kerma, for equilibrium of charged particles. For more details see [56] or [57]

## Results

**Fig. 4:**
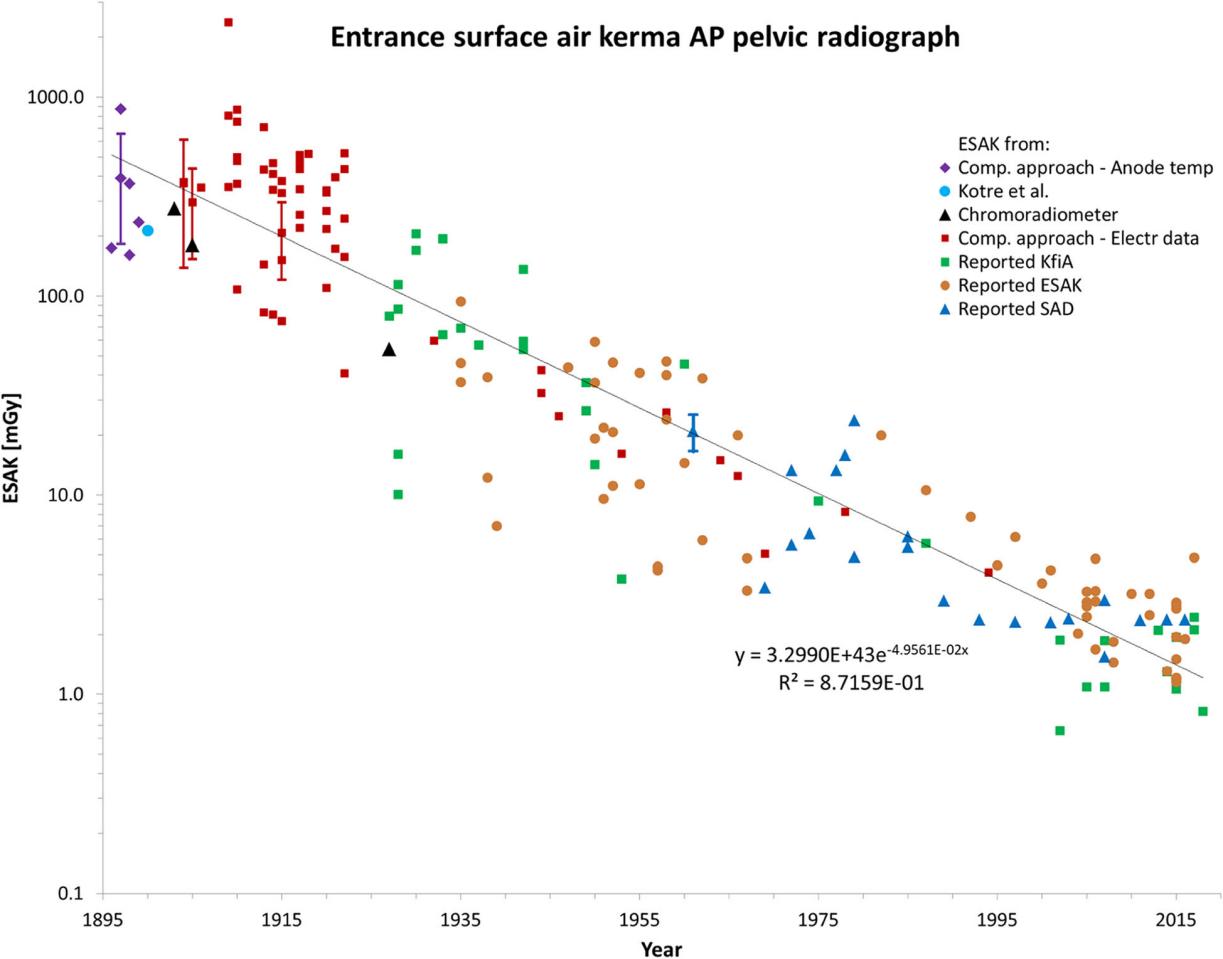
Entrance surface air kerma (which includes backscatter) for pelvic radiographs 1896–2018 (*n* = 182). “Error bar” in 1897 is an estimate of sensitivity to anode temperature (for a 100 °C change; see Table 6). The bars for 1904 and 1905 are from Tables 8 and 9, respectively. The 1915 bar (40% uncertainty) might be typical for 1910–1926. After 1927 an uncertainty in all values of about 20% seems reasonable. This is shown at 1961; the graphical size of this uncertainty bar holds at all times (1927–2018) due to the logarithmic scale. All references in Additional file 1: ES XV

Results are presented more or less chronologically. All collected ESAK values are shown together in Fig. 4.

### Results from computational approach 1896–1900

Directly after 1895 information on X-ray exposures was always incomplete. However, if we interpret the various descriptions of the glowing anode during radiography as a quantitative indication of the temperature, X-ray dose estimation comes within reach. Assuming an anode temperature of 750 °C (Additional file 1: ES IV), the maximum electrical loads of two popular tubes, the Jackson focus tube and one according to Chabaud, were estimated to be roughly 5 and 10 W, respectively.

We found six early studies in which these tubes had been used. Setting the glass wall thickness at 0.6 mm and the backscatter factor at 1.17, the results in Table 5 were obtained.

**Table 5:** Some exposure parameters from before 1900 and the corresponding ESAK

Author	Year	kVp	P_sec_^a,b^	I_av_sec_^a^	t_expo_^a^	FSD^a^	KfiA at 1 m	ESAK
		kV	W	mA	min	cm	μGy/mAs	mGy
Macintyre [58]	1896	96	5	0.066	12	29	362	174
Rosenfeld [59]	1897	60	5	0.106	50	39	220	391
Foveau de Courmelles [60]	1897	75	10	0.170	30	29	282	870
Gocht [61]	1898	80	10	0.159	10	39	300	160
Isenthal [62]	1898	80	5	0.080	25	30	304	367
Walsh [63]	1899	80	10	0.159	30	55	294	235

^a^P_sec_ = secondary power, I_av_sec_ = average tube current, t_expo_ = time, FSD = focus-skin distance

^b^The KfiA effectively depends on the available secondary electrical power only, as the tube current and kVp are largely interchangeable

As the available indications of the anode temperature were only approximate, the dose estimates in Table 5 must be considered as tentative. Table 6 gives an idea of the sensitivity of the output to possible variations in the basic parameters (Additional file 1: ES XII). As for large changes in anode temperature (ΔT) the effect on KfiA depends on the sign of ΔT, Table 6 shows both the change toward lower and higher KfiA.

**Table 6:** Sensitivity of the KfiA to basic parameters for an 1896 X-ray tube with glowing anode

Rosenfeld (1897) [59]	T_anode_	ε	t_exposure_	Wall	FSD	KfiA	Total change KfiA towards	
	°C		min	mm	cm	mGy	lower values	higher values
Parameter value	750	0.69	50	0.60	39	334		
Variation considered^a^	100^a^	0.14	5	0.15	3		51%	67%

^a^The change of 100 °C in T_anode_ could be a realistic uncertainty (say standardard deviation), but we simply do not know due to lacking information. The other uncertainties in basic parameters are deemed realistic

### Kotre et al.

Kotre et al. performed for the period 1899–1902 a nice replication study using old equipment and logbooks with exposure data from the Forth Banks Infirmary in Newcastle. They reported a median ESAK of 189 mGy; according to their (skewed) Fig. 4 the average was about 213 mGy [1].

### Chromoradiometers (Additional file 1: ES XIII and ES I)

We only found three chromoradiometer measurements, from Levy-Dorn [37], Kienböck [19] and Dalton et al. [38].

### Results from computational approach 1900–1927 (Additional file 1: ES XIV)

In the period 1900–1910 the amount of quantitative information slowly increased. Towards 1910 operators were cognizant of the parameters characterizing an exposure and they realized that the numerical values were relevant to others. This is nicely illustrated by an exposure slide rule from 1909 described by Janus from the firm Reiniger, Gebbert und Schall [39]. Also an article by Jaugeas from 1910 bears witness of maturity [40]. Table 7 shows the datasets up to 1910 we were able to retrieve. Thermal anode loading never exceeded the limits estimated in Additional file 1: ES IV.

**Table 7:** Some exposure parameters from the period 1900–1910 and the corresponding ESAK^a^

Author	Year	Inter-	V_prim_^b,c^	I_prim_^b,c^	kVp	I_av_sec_	t_expo_	FSD	KfiA at 1 m	ESAK
		rupter^b,d^	V	A	kV	mA	s	cm	μGy/mAs	mGy
Beck [64]	1904	W	115	1.5	72	0.52	210	23	320	374
Thurston-Holland [65]	1904	Hg	24	8	45	2.4	120	29	316	370
Biddle [66]	1905				72	15	10	30	253	296
Albers-Schönberg [67]	1906	W	70	9	81	1.68	162	40	243	352
Janus [39]	1909				104	5.1	40	39	309	353
Arthur^e^ [68]	1909	Hg	50	8	84	3.0	240	25	247	2374^e^
,,	1909	W	80	15	84	3.1	80	25	247	807
Béclère [69]	1910				110	7	20	29	337	477
Jaugeas [70]	1910				109	15	17.5	29	325	863
,,	1910				110	25	6	29	330	501
,, ^f^	1910				74	10	5	29	213	108
Tousey [71]	1910	W	110	16.5	90	4.4	45	35	266	366
,,	1910				90	9	45	35	266	753

^a^Wall thickness 0.85 mm glass. Inductor and ion tubes only

^b^Only relevant before 1910 when information on primary circuits was needed (and available)

^c^V_prim_ = DC-voltage primary circuit, I_prim_ = average primary current

^d^Interrupter: W=Wehnelt electrolytic break, Hg = mercury break

^e^Exceptionally high ESAK. All input taken from pag 101 in [68]. Their Fig. 14 on page 25 shows a correct configuration for voltage and current measurement, and the dose should generally not cause burns. But note also the uncertainty in our estimates

^f^Jaugeas: This low value is for “new Lumière plates and Gehler Folie screens”

Tables 8 and 9 show two typical sets of early exposure parameters with what might be reasonable uncertainties (we treat them all as standard deviations). According to the approach described in Additional file 1: ES XII, the final uncertainty in KfiA might be typically 50 to 60%.

**Table 8:** Uncertainty estimate for a case with information on primary and secondary circuit

Beck (1904) [64]	ε	α	I_prim_	V_prim_	t_exposure_	Wall	FSD	KfiA	Total
			A	V	s	mm	cm	mGy	uncert.
Parameter value	0.69	0.17	1.5	115	210	0.85	23	320	
Uncertainty assum.	0.14	0.07	0.30^a^	25^b^	21	0.15	3		63%

^a^Current was specified as 1 to 2 A

^b^Voltage specified as 110-120 V, but as loss over series resistance (rheostat) cannot be excluded we raised the uncertainty

**Table 9:** Uncertainty estimate for a case with information on secondary circuit

Biddle (1905) [66]	HV	ε	I_av_sec_	t_exposure_	Wall	FSD	KfiA	Total
	kV		mA	s	mm	cm	mGy	uncert.
Parameter value	72	0.69	15	10	0.85	30	206	
Uncertainty assum.	10	0.14	3	2.5	0.15	3		48%

After 1910 more of the published exposure data were complete and the computation of the ESAK was rather straightforward. Up to 1922 we assumed no filtration apart from the 0.85 mm glass and a column of air equal to the focus skin distance minus the radius of the X-ray tube (taken as 10 cm). After 1922 the use of filters was mentioned more frequently, and often recommended, but clearly not standard. The BSF was estimated casewise. The total number of computed doses before 1927 was 53.

### Retrieved results from explicit (“modern”) dose measurements after 1927 (Additional file 1: ES XIV and ES XV)

After1927 ionization chamber based dose measurements were reported. A few doses had been measured with thermoluminescence dosemeters or a “KAP-meter”, the well-known large flat ionization chamber in the primary beam giving the product of kerma-in-air and beam area. In total 114 results were retrieved. Some additional points (*n* = 11) were still obtained by using our computational approach.

**Fig. 5:**
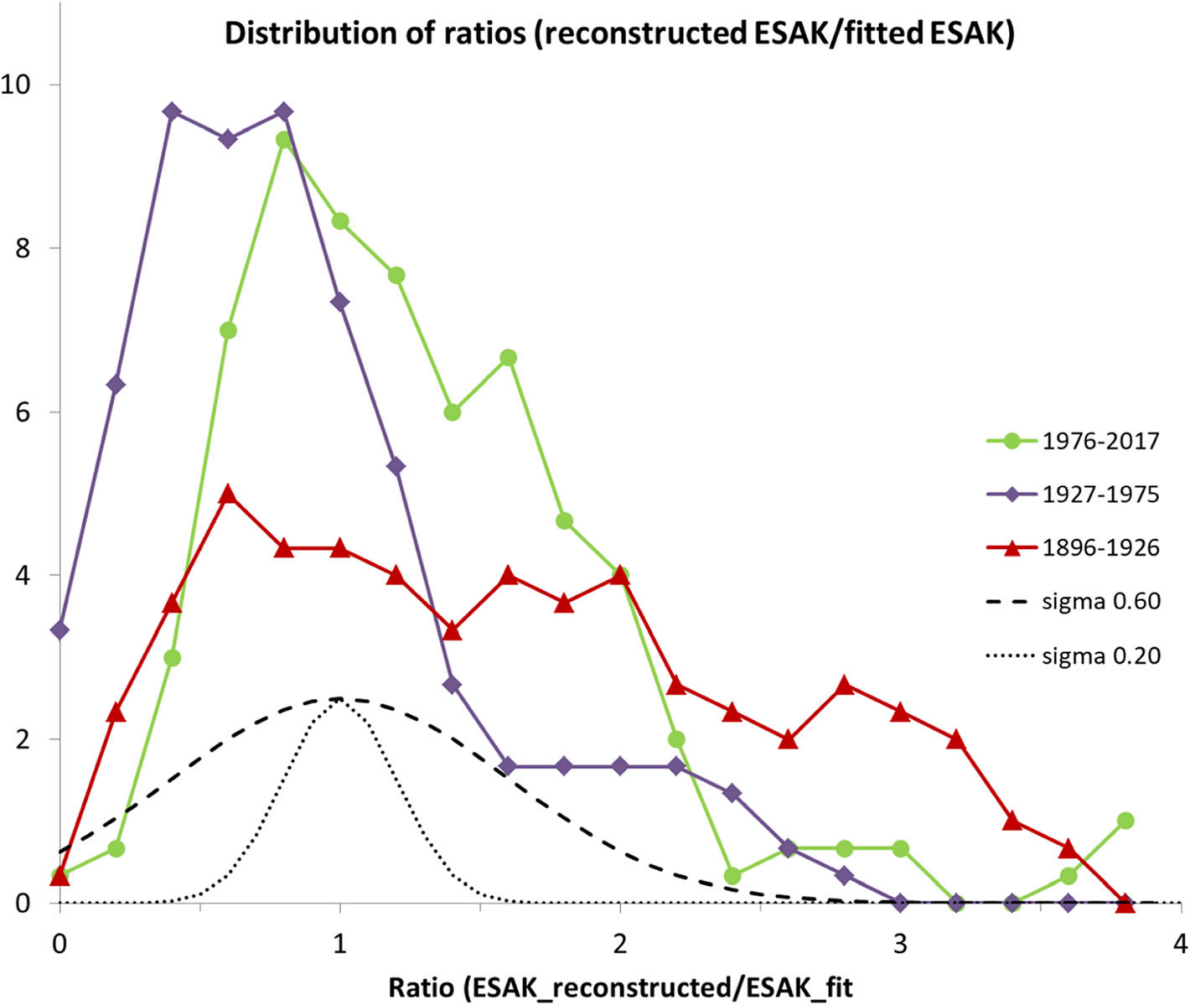
Distributions of the ratio ESAK(reconstructeded)/ESAK(fit) for three intervals of time. Points summed within intervals (x-axis) of 0.2 and curves smoothed. Dashed curve gives shape of hypothetical distribution of doses according to an estimated maximum standard deviation of 60% in our 1900–1910 dose reconstructions. Dotted curve represents the spread of 20% in doses assumed for 1927–1957

The data collected in Fig. 4 could well be fitted with a single exponential. To get a quantitative impression of the spread in ESAK over time, we calculated the ratio of each individual dose and the exponential fit. Distributions of this ratio are shown in Fig. 5 for three periods. Notice that even the estimated maximum uncertainty of about 60% in the dose reconstruction for the early period 1900–1910 corresponds to a less wide distribution than actually observed for the 1896–1926 period.

**Fig. 6:**
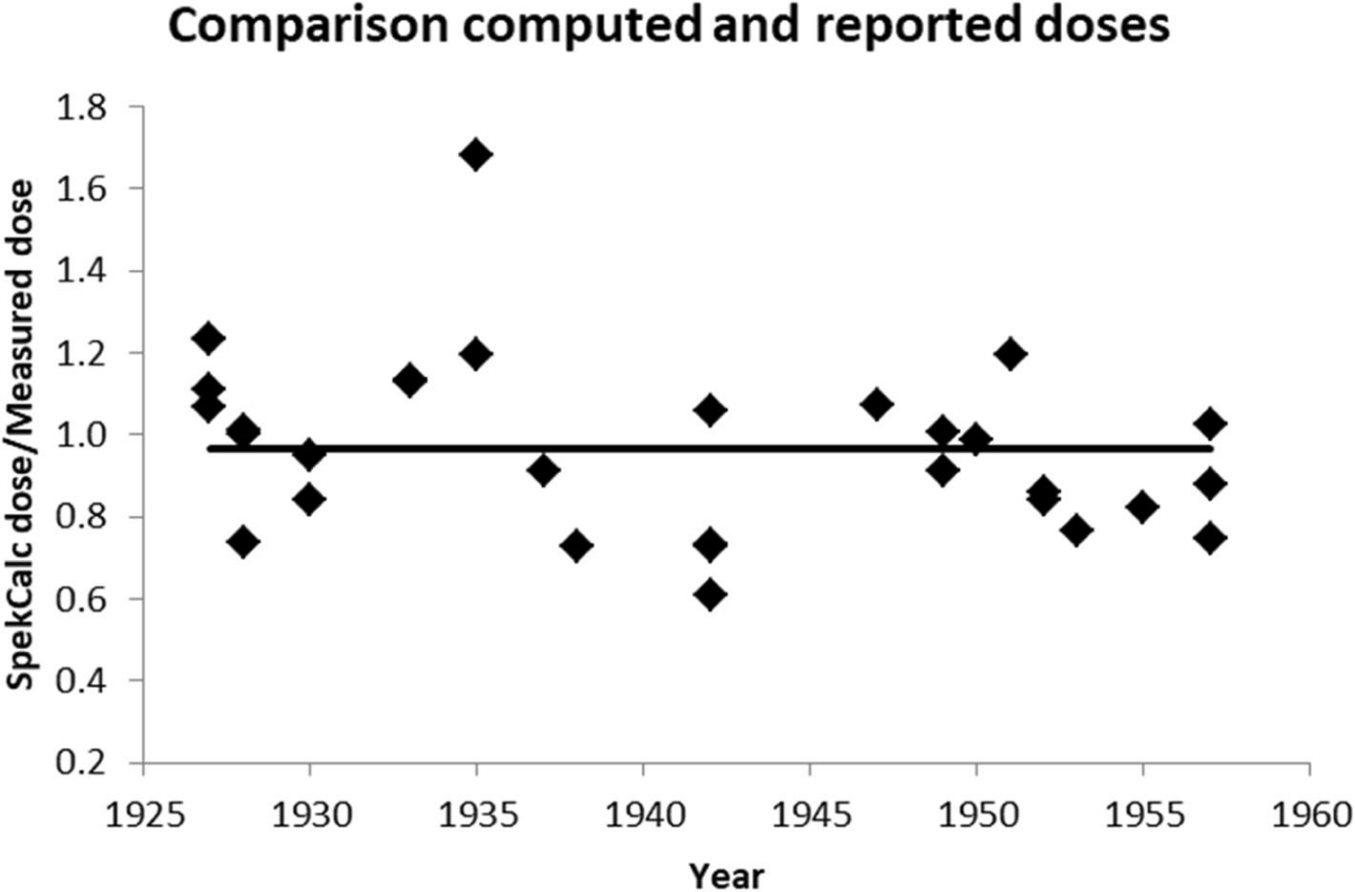
Ratio of dose according to our computational approach and reported measured dose (*n* = 32)

A reasonable number of authors reported both an explicit dose and all relevant exposure parameters needed for our computational approach, which allowed a check on our computations. Figure 6 shows the ratio of calculated and reported doses: the average is 0.97 ± 0.21 (*n* = 32).

## Discussion

When starting the project, we hardly expected that dose reconstruction would be possible for the first decades of radiology. Surprisingly it was, with estimated uncertainties of the order of 40%–60% for the period 1900–1926. It appeared that these uncertainties were still surpassed by the variations in dose caused by differences in X-ray systems and their use. Large variations in dose persisted up to current times. Nevertheless, a substantial decrease in average dose was evident: since 1896 the skin dose for a radiograph in the pelvic region was roughly lowered by a factor of 400.

In our dose reconstruction we tried to use all available sources of information. Kotre et al. [1] reported the dose incurred by patients in the period 1899–1902, the single explicit dose estimate we found from before 1927. Chromoradiometer readings were available from three studies only, and although reportedly inaccurate, they are valuable because the results are methodologically independent from our computational approach. The latter was our mainstay for the period 1896–1926. For 1896–1899 an elementary assessment of the physics of heat loss by radiation of two 1896 X-ray tubes was at the basis of our computations. Due to lack of knowledge of the actual anode temperatures the results are rather speculative, but they seem compatible with the other data. The doses from later specifications of exposure parameters are more reliable, certainly after 1910, when all relevant physical parameters were evidently understood and experimentally assessable.

Our assessment of spark gap data, the various penetrometers, and the efficiency of different old röntgen systems regarding electrical transformation and X-ray production, is the first in its kind as far as we know. Simulations with SpekCalc were very helpful in extracting “modern” information from antiquated data. Generally our evaluations revealed considerable variability in all old data, but the relations between spark gap and high voltage were more concordant than the literature had led us to believe.

Comparison of measured doses (from the literature) with doses calculated using our computational approach was possible for the period 1927–1957, yielding a ratio of 0.97 ± 0.21. The relatively small standard deviation, compared to our pre-1927 uncertainty estimate of 40–60%, is probably due to more stable systems and increased accuracy of the data reported after 1927.

Nearly all low-lying points in Fig. 4 stem from investigators who showed that dose reduction was possible according to their standards. Braun [41] and Saupe [42] in 1928, Ardran and colleagues in 1938, 1939, 1953 and 1957 [43–45], Billings [46] in 1957 and Persliden [47] in 2002 belong to this group. Their doses are clearly not representative for the average practice at the time of reporting. But are other reported values? It is possible that X-ray users who published on radiation dose already adhered to stricter than average dose standards, implying that Fig. 4 might give a more favorable impression than genuine surveys would.

We saw only one other study with doses for an anteroposterior pelvic radiograph as a function of time: Huda et al. [48] published values for 1965–2002 that fall well within our cloud of data.

Because of the relatively large number (*n* = 182) of collected doses, their spread could quantitatively be assessed (Fig. 5). It turns out that the dose distributions are skewed and reminiscent of distributions reported in other dose surveys. The curve for the period 1896–1926 suggests that the spread in doses is largest in the first decades of radiology. This seems understandable considering the large variability in systems and their use. Some used already intensifying screens, which lowered the dose by a factor of about 5 to 10 compared to working without screens. Additional variability in the ion tube era was caused by the unintended change in gas pressure in the tube, whereas extremely high doses could result from inappropriate use. Mitchell et al. [49], Cassidy [50] and Deutschländer [51] reported wet desquamation or necrotization following exposure, implying doses higher than 18 Gy [52]. The occurrence of such severe accidents suggests that the 1896–1926 distribution in Fig. 5 actually has a long tail to high doses.

For the time after 1927 we found explicit dose data, in the form of ESAK, KfiA and SAD. No systematic differences on the basis of data origin are apparent. Certainly in these later years the large spread in doses will have been caused by the users and their equipment, not by errors in measurements or calculations. Note that this variability signaled a large but unused potential for dose reduction by optimization. For some time now the realization of this potential is pursued by the introduction of diagnostic reference levels [53, 54]. This is for good reason, as our historical review proves it never happened by itself in the past century.

## Conclusions

Reconstruction of the skin dose (ESAK) for pelvic radiography was performed for the period before 1927. For the time after 1927 doses were retrieved from the literature. At all times the doses showed a large spread. On average a decrease in dose with a factor of about 400 was observed. The methods of dose reconstruction developed here might be useful in future studies.

## Additional file

Additional file 1: Background information, details on calculations including simulations using SpekCalc and some supporting results. (PDF 2020 kb)

## References

[1] Kotre CJ, Little BG (2006). Patient and staff radiation doses from early radiological examinations (1899−1902). Br J Radiol.

[2] (2005) 3 quantities and units for measurement and calculation in medical X-ray imaging. J ICRU 5:25–34. 10.1093/jicru/ndi025 10.1093/jicru/ndi025 24170872

[3] Wall BF (1996). How to assess the dose to the patient - refresher course R-02.

[4] Kaye GWC, Binks W (1929). The EVALUATION OF the X-ray pastille dose in Rontgen (r) units. Br J Radiol.

[5] Poludniowski G, Landry G, DeBlois F, Evans PM, Verhaegen F (2009) SpekCalc: A program to calculate photon spectra from tungsten anode x-ray tubes. Phys Med Biol 54:N433–N438. 10.1088/0031-9155/54/19/N01 10.1088/0031-9155/54/19/N01 19724100

[6] Hubbell JH, Seltzer S (1995) Tables of X-ray mass attenuation coefficients and mass energy-absorption coefficients from 1 keV to 20 MeV for elements Z = 1 to 92 and 48 additional substances of dosimetric interest, NIST standard reference database 126. https://www.nist.gov/pml/x-ray-mass-attenuation-coefficients. Accessed: 9 Sept 2018

[7] XCOM NIST XCOM: Element/Compound/Mixture. https://physics.nist.gov/PhysRefData/Xcom/html/xcom1.html. Accessed 20 Aug 2018

[8] Graph Grabber 2.0 https://www.quintessa.org/software/downloads-and-demos/graph-grabber-2.0 Accessed: 1 Sept 2018

[9] Ullmann HJ (1921). The practical application of the sphere gap to roentgenotherapy. AJR Am J Roentgenol.

[10] Surgeon General of the Army (1920). United States army X-ray manual.

[11] AIEE (1969). E 61: Spark gap voltages, in CRC handbook of chemistry and physics.

[12] Spiegler G, Zakovsky J (1928). Der Halbwellenapparat der Röntgentechnik. Fortschritte auf dem Geb Röntgenstrahlen.

[13] Peek FW (1915). Dielectric phenomena in high voltage engineering.

[14] Schall WE (1932). X rays: Their origin, dosage and practical application.

[15] Rieber F (1922). Standardization of the measurement of tube potential. AJR Am J Roentgenol.

[16] Benoist ML (1902). Définition expérimentale des diverses sortes de rayons X, par le radiochromomètre. Comptes Rendus Acad Sci.

[17] Hackenbruch PT, Berger W (1915). Mittlerer expositions-Tabelle in Milliampèresekunden. Vademekum für die Verwendung der Röntgenstrahlen und des Distraktionsklammerverfahren im Kriege.

[18] Luboshez BE (1925). On measuring and expressing X-ray quality in radiography. Br J Radiol BIR Sect.

[19] Kienböck R (1905). Über Dosimeter und das quantimetrische Verfahren. Fortschritte auf dem Geb Röntgenstrahlen.

[20] Albers-Schönberg H, Walter B (1910). Die Röntgentechnik.

[21] Muir J, Reid A, Harlow FJ (1927). A manual of practical X-ray work.

[22] Christen T (1914). Zur Theorie und Technik der Härtemessung. Fortschritte auf dem Geb Röntgenstrahlen.

[23] Christen T (1913). Messung und Dosierung der Röntgenstrahlen. Fortschritte auf dem Geb Röntgenstrahlen Ergänzungsband.

[24] Gaiffe G (1905). De l’emploi d’un milliampèremètre sur le circuit d’un tube de rayons X. Arch d‘électricité Médicale.

[25] Coolidge WD (1913). A powerful Röntgen ray tube with a pure electron discharge. Phys Rev.

[26] Goetze O (1923). Verfahren und Glühkathodenröntgenröhre zur Erzeugung scharfer Röntgenbilder (patent 370022).

[27] Harrison RM (1994). X-rays: Half-value layers 0.01-8.0 mm AI (approx. 6-150 kV peak potential difference). Br J Radiol Suppl.

[28] National Council on Radiation Protection and Measurements (1981). Radiation protection in pediatric radiology: recommendations of the National Council on radiation protection and measurements. Report 68.

[29] Grigg ERN (1965). The trail of the invisible light. From X-Strahlen to radio(bio)logy.

[30] (1928) International X-ray unit of intensity. Br J Radiol 1:363–364. 10.1259/0007-1285-1-10-363

[31] Behnken H (1924). Die Eichung von Dosismessern in der Physikalisch-Technischen Reichsanstalt. Fortschritte auf dem Geb Röntgenstrahlen.

[32] Gleßmer-Junike S (2015) X-Strahlen, radiometer und Hauteinheitsdosis. Thesis. Universität Hamburg. http://ediss.sub.uni-hamburg.de/volltexte/2015/7181/

[33] ICRU (1954). Recommendations of the international commission on radiological units. Br J Radiol.

[34] ICRP (1955) Supplement no. 6. Recommendations of the ICRP. Br. J. Radiol. (Suppl. 6):100

[35] International Commission on Radiological Units and Measurements (ICRU) (1962). Radiation quantities and units - report 10a. National Bureau of standards handbook 84.

[36] ICRU 33 A (1981). ICRU report 33 - radiation quantities and units pub: International commission on radiation units and measurements, Washington D.C. USA issued 15 April 1980, pp.25. J Label Compd Radiopharm.

[37] Levy-Dorn M (1903). Schutzmassregelen gegen Röntgenstrahlen und ihre Dosirung. Dtsch Med Wochenschr.

[38] Dalton CHC (1927). The comparative efficiency of different types of transformers. Br J Radiol BIR Sect.

[39] Janus F (1909). Der Expositionsmesser, ein neues Hilfsinstrument für Röntgenaufnahmen. Fortschritte auf dem Geb Röntgenstrahlen.

[40] Jaugeas F (1911). Rapid and instantaneous radiology. Arch Roentgen Ray.

[41] Braun R, Hase H, Küstner H (1928). Über die in der Diagnostik verabfolgten Dosen in R-Einheiten. Fortschritte auf dem Geb Röntgenstrahlen.

[42] Saupe E (1928). Über die bei diagnostischen Arbeiten verabreichten Röntgenstrahlendosen in R-Einheiten. Fortschritte auf dem Geb Röntgenstrahlen.

[43] Ardran GM, Crooks HE (1953). A comparison of radiographic techniques with special reference to dosage. Br J Radiol.

[44] Ardran GM (1956). The dose to operator and patient in X-ray diagnostic procedures. Br J Radiol.

[45] Ardran GM, Crooks HE (1957). Gonad radiation dose from diagnostic procedures. Br J Radiol.

[46] Billings MS, Norman A, Greenfield MA (1957). Gonad dose during routine Roentgenography. Radiology.

[47] Persliden J, Beckman KW, Geijer H, Andersson T (2002). Dose-image optimisation in digital radiology with a direct digital detector: an example applied to pelvic examinations. Eur Radiol.

[48] Huda W, Nickoloff EL, Boone JM (2008). Overview of patient dosimetry in diagnostic radiology in the USA for the past 50 years. Med Phys.

[49] Mitchell JS, Haybittle JL (1955). Carcinoma of the skin appearing 49 years after a single diagnostic roentgen exposure. Acta Radiol.

[50] Cassidy P (1900). Report of a severe X-ray injury. Med Rec.

[51] Deutschländer (1899). Beitrag zu dem Kapitel der Hautverbrennung durch Röntgenstrahlen. Fortschritte auf dem Geb Röntgenstrahlen.

[52] Stewart FA, Akleyev AV, Hauer-Jensen M (2012). ICRP PUBLICATION 118: ICRP statement on tissue reactions and early and late effects of radiation in normal tissues and organs — threshold doses for tissue reactions in a radiation protection context. Ann ICRP.

[53] ICRP (2001). Diagnostic reference levels in medical imaging: Review and additional advice. ICRP supporting guidance 2. Ann ICRP.

[54] Vañó E, Miller DL, Martin CJ et al (2017) ICRP Publication 135: Diagnostic Reference Levels in Medical Imaging. Ann ICRP 46:1–144. 10.1177/0146645317717209 10.1177/0146645317717209 29065694

[55] Snook HC (1908). A new Roentgen generator. Arch Roentgen Ray.

[56] Jennings WA (2007). Evolution over the past century of quantities and units in radiation dosimetry. J Radiol Prot.

[57] Mould RF (1993). A century of x-rays and radioactivity in medicine: with emphasis on photographic records of the early years.

[58] Macintyre J (1896). Roentgen rays: photography of renal calculus; description of an adjustable modification in the focus tube. Lancet.

[59] Rosenfeld G (1897). Die Diagnostik innerer Krankheiten mittels Röntgenstrahlen.

[60] Foveau de Courmelles F-V (1897). Traité de radiographie médicale et scientifique.

[61] Gocht H (1898). Lehrbuch der Röntgen-Untersuchung: Zum Gebrauche für Mediciner.

[62] Isenthal AW, Snowden Ward H (1898). Practical radiography - a handbook of the applications of the X-rays.

[63] Walsh D (1899). The Röntgen rays in medical work.

[64] Beck C (1904). Röntgen ray diagnosis and therapy.

[65] Holland CT (1904). Description of plate: Plate I: the left and right kidney regions: Plate a. (the left) shows the shadow of one stone. Plate B. (the right) shows the shadow of three stones. J Röntgen Soc.

[66] Biddle JG (1905). Typical “roentgen” Equipments.

[67] Albers-Schönberg (1906). Die Röntgentechnik: Lehrbuch für Ärzte und Studierende.

[68] Arthur D, Muir J (1909). A manual of practical X-ray work.

[69] Béclère H (1910). Le Radiodiagnostic des affections du foie.

[70] Jaugeas F (1910) Radiographie rapide et radiographie instantanée. J Radiol Belge:601–629

[71] Tousey S (1910). Medical electricity and Röntgen rays.

