# Supplementary material for: The skin dose of pelvic radiographs since 1896
Source: Insights Imaging. 2019 Mar 29;10:39. doi: 10.1186/s13244-019-0710-1 (PMC6439109; doi:10.1186/s13244-019-0710-1)
Supplement: Supplementary file 1 — Background information, details on calculations including simulations using SpekCalc and some supporting results. (PDF 2020 kb) [file 13244_2019_710_MOESM1_ESM.pdf]

Additional file 1 (Electronic Supplement)

The skin dose of pelvic radiographs since 1896

Gerrit J. Kemerink, Gerhard Kütterer, Pierre J. Kicken, Jos M.A. van Engelshoven, Kees J. Simon, Joachim E. Wildberger.

Content

ES I. Physico-chemical radiometers	2
ES II. Software and other resources	4
ES III. X-ray systems: high-voltage generator and X-ray tube	5
ES IV. Maximum heat dissipation in early ion tubes	7
ES V. The kVp from a Spark Gap	11
ES VI. The kVp from penetrometer hardness	12
ES VII. Electrical information on primary and secondary circuit of ion tube systems	21
ES VIII. Anticathode/anode material	24
ES IX. Filtration (glass envelope of X-ray tube, additional filter and air)	24
ES X. X-ray efficiency of old pulsed systems compared to a DC system	25
ES XI. Summary of data and literature used in determining X-ray efficiency	28
ES XII. Estimation of uncertainty in the results of our computational method	33
ES XIII. Results from chromoradiometers	34
ES XIV. Results, reconstructed and retrieved	35
References ES I – ES XIV	39
ES XV. References for Fig. 4 (in main text)	48

Electronic Supplement

The skin dose of pelvic radiographs since 1896

Gerrit J. Kemerink, Gerhard Kütterer, Pierre J. Kicken, Jos M.A. van Engelshoven, Kees J. Simon, Joachim E. Wildberger.

ES I. Physico-chemical radiometers (PCR)

The scale of a PCR was calibrated with the dose that caused some particular effect on the skin, which one might call the reference skin effect (RSE). If one knows the PCR reading for an RSE in its own unit ($Reading_{RSE}$) and in some modern equivalent (e.g. $KfiA_{RSE}$), it is possible to convert the reading for a given exposure ($Reading_{expo}$) into modern units according to

$$KfiA_{expo}\,[mGy] = Reading_{expo}\,\frac{KfiA_{RSE}}{Reading_{RSE}}\,[mGy].$$

We will see below that the dosimeters that are relevant for us were actually characterized by the epilation dose, estimated at about 3.9 Gy for the soft unfiltered radiation initially used in radiology. We will refer to this value as the “pastille dose” [1].

Physico-chemical radiometers were primarily designed for application in radiotherapy. A nice overview is given by Gleβmer-Junike [2]. Here we only discuss the three devices that are relevant for us (several more existed): The Holzknecht Chromoradiometer, the Kienböck Quantimeter and the Pastille of Sabouraud-Noiré.

The Holzknecht Chromoradiometer was introduced in 1902 and consisted of a salt in the form of a small pill that gradually changed from transparent and nearly colorless to green. The unit 1 H was defined as 1/3 of the dose that caused a light erythema of the skin of the face [3]. It turned out that 5 H caused epilation, 6 H erythema. Holzknecht did not disclose the composition of his radiometer.

Kienböcks Quantimeter (1905) used photographic paper, i.e. cardboard with a layer of rather insensitive “chlorbromsilbergelatine”. He chose a new unit X, defined by 2 X = 1 H [4]. The photographic method was generally considered superior to Holzknecht’s procedure, but was more elaborate because the photographic paper needed development.

A third dosimeter was a pastille made of a piece of paper covered with $BaPt(CN)_4.4H_2O$, developed by Sabouraud and Noiré in 1904 (“chromoradiomètre”) [5]. Its color went under irradiation from bright green to dark yellow-brown; the initial color was indicated with the letter A, the color after a full dose with the letter B. Its unit, SN (some use also the letter B), was also linked to Holzknecht’s scale according to 1 SN = 5 H = 10 X. The authors stated “une dépilation totale d'une région donnée du cuir chevelu, sans radiodermite, sans érythème et sans alopécic définitive”, i.e. a temporary loss of hair on the scalp but no erythema or dermatitis. The SN-pastille was still in use after 1930.

For all radiometers reference scales were available to convert a color or a shade of grey into a multiple or fraction of the units H, X or SN. Unfortunately these dosimeters were far from perfect: temperature, moisture, ageing, ambient light during use, the light that was used during read-out and subjectivity of the reader in assessing subtle color or grey differences could all affect the outcome. For the Quantimeter things could go wrong during development: the chemical composition of the developer, temperature and time were critical.

Apart from the imperfections of the radiometers, there was the problem of a poorly defined radiation induced physiological change of the skin at the fundament of the dose unit. In 1924 in a multisite comparison Grebe and Martius found large variations in erythema doses. It turned out that these differences were mainly caused by considering different degrees of erythema. The ratio of the erythema doses of the institutes visited by Grebe and Martius and their own value ranged from 0.66 to 2.58, illustrating the problem [6].

Roughly in the period 1925 – 1935, while the old radiometers were still partly around, the new ion chamber based dosimetry became mature. It is in this period that better measurements of the erythema dose and more accurate calibrations of radiometers were performed. However, the spread in the number of r for an erythema dose remained initially rather large [7]. Schall [8] and Reisner [9] illustrated the nearly insurmountable difficulties in quantifying erythema.

W.E. Schall, from a London firm and another person than the one above, provided an instructive graph in his book “X Rays” (1932) [10], shown in Fig. ES1.

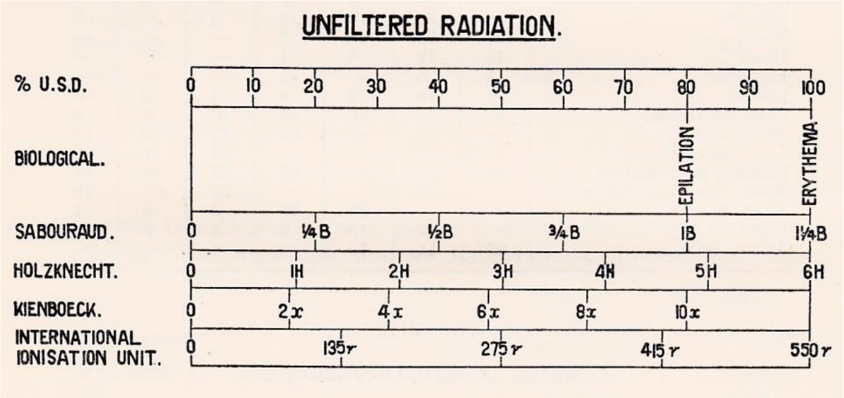

Fig. ES1 The relation between the reading of different radiometers and the corresponding dose in r for unfiltered radiation (170 kVp). The skin erythema and epilation dose are also shown in r and as radiometer readings. From Schall [10]

His erythema dose is set at 550 r; he doesn’t state the field size for which the graph applies, but judging from contemporary literature it must be 6 cm x 8 cm (see for instance Reisner [11,9] and Neeff [12]. One further notes from Schall’s graph that 1 B (1 SN), the pastille dose, corresponds to about 438 r. The graph also illustrates the difference between the epilation and the erythema dose (according to Reisner the difference is about 20% [11]).

Kaye gives an overview of pastille doses in r for low-kVp unfiltered X-rays [1]. From his Table VI (p 573), omitting the 160 and 185 kVp values (such high kVp was never used for radiographs), but including his own results in Table V on P 571, one finds an average of 446 r with a standard deviation of 72 r. Note that this value is similar to that given above (although that was for a higher kVp). The modest standard deviation may reflect improved standardization over the years. Such a pastille dose, measured at that time, seemed to us the most appropriate value to be used together with the radiometer readings retrieved from the literature. We will therefore use a pastille dose equaling 1 SN = 5 H = 10 X = 446 r (3.906 Gy).

Probably it is good to stress that the relation between the number of roentgens, measured with an ionization chamber free in air (as was common practice), and the effect of that amount of radiation on the skin was dependent on the size of the area of the exposed skin. If a certain effect in a very small exposed field was induced for a reading Y, the same effect would already be induced in a large field for a reading of Y/BSF, with BSF the backscatter factor. The dose in air given above for 1 SN corresponds thus to the epilation dose divided by BSF, with the BSF for a 6x8 cm^2 field. However, relevant for us is only the number of r's corresponding to color B (i.e. 1 SN), and that this number refers to the quantity kerma free in air.

ES II. Software and other resources

The most important tools and resources used are SpekCalc [13-15], the NIST attenuation data [16,17], Graph Grabber [18] and Microsoft Excel [19].

The software program SpekCalc enables the calculation of röntgen spectra for x-ray tubes with a tungsten anode. It also estimates the kerma free in air. The program supports a wide range of DC-tube potentials (40–300 kVp) and anode angles (6° – 30°, recommended, but we will use it also for 45°). Several absorbing materials (aluminum, copper, beryllium and air) can be placed in the beam. In principle the intrinsic and added filtration of a tube can thus be accounted for. However, as small and unintended variations in tubes occur, SpekCalc allows compensation for these by adjustment of a scaling parameter, N_f, for instance on the basis of measured and calculated KfiA. We will see below that the average of the ratio of a series of 32 measured doses (retrieved from the literature) and computed doses was very close to one (0.97±0.21). So we left N_f at its default of 0.68. A second parameter, P, is related to characteristic radiation and should not be changed from the default value of 0.33 [13].

In our simulations one pulse of a pulsed power supply was sampled at 31 points and the X-ray spectra in these points were simulated, taking the current at that point into account when forming the composite spectrum. The spectrum-bin in SpekCalc was set to 1 keV. The simulations were always also done for DC voltage. Doubling the number of sample points for a sine voltage and constant current affected KfiA with less than 0.3%, suggesting our sampling frequency (31 per pulse) was adequate.

The free program Graph Grabber [18] was used to digitize a few voltage and current waveforms that had been observed on early X-rays systems, the digitized data being used with

SpekCalc to obtain the composite X-ray spectrum generated by the pulses. Graph Grabber was also very convenient for digitizing all sorts of graphs.

Microsoft Excel was used for nearly all calculations. Conversions that had to be done repeatedly were automated.

ES III. X-ray systems: high-voltage generator and X-ray tube

We consider six combinations, addressing properties that are of interest to our dose reconstruction. More complete information can be found in the 1920 book "The Principles and Practice of Roentgenological Technique" by Hirsch (freely accessible on the Internet) [20] and in Kütterer's book on the first two decades of radiology [21].

A. Inductor with interrupter and ion tube

The first X-ray systems were predominantly of this type. Different types of interrupters were in use: mechanical interrupters, mercury interrupters (of the dipping and jet type) and the Wehnelt and Simon electrolytic interrupters. The form of the voltage and current pulses that were used to excite the gas tube could vary considerably. These waveforms depended on the construction of the inductor, the presence of a capacitor in the primary circuit, the type and adjustment of the interrupter, wiring and tube properties. Moreover, the partial vacuum of the tube was not constant: dependent on the use of the tube gas would be trapped or released. This behavior made the X-ray output rather variable, even over short periods. Important for a meaningful milliampere meter reading of the current through the tube was the absence of inverse (or "closing") current ([20], page 90). Synonyms for "ion tube" are "gas tube" and "cold cathode tube"; the pressure within the tube was of the order of 1 to 10 * 10^{-3} mbar [22].

B. Transformer, generally of the Snook type, and ion tube

The transformer was more stable and permitted higher power than the inductor. The Snook transformer system (1907) had mechanical rectification, the rectifier only conducting during the extremes of the (theoretically) sine-shaped voltage (e.g. from 20° before to 20° after the voltage crest) [23]. Several manufacturers made such rectifier systems. The purpose of using only the voltage crests was reducing the amount of soft radiation by leaving out the low voltages in the sine waveform. However, load switching at high-voltage often caused unpredictable oscillations and pulse distortions. Large losses could occur in arcs if the contacts of the mechanical rectifier were not well adjusted. A transformer with a mechanical rectifier was also called "interrupter-less system", as the classical interrupter needed on an inductor coil driven by a DC-source was absent. With the inductor-ion-tube system the transformer-ion tube had in common that the tube current changed with the voltage (i.e. current was not saturated as normally the case in a Coolidge tube). The mechanical rectifier disappeared after 1926 when the vacuum thermionic valve, the so called kenotron [24,25], had been perfectioned.

C1. Transformer with mechanical ('Snook') rectifier and Coolidge tube

Transformer-Coolidge systems were much more constant in their output than inductor-ion tube combinations. A characteristic of the normal use of a Coolidge tube is a fixed (emission limited) current. Sources of variability were fluctuations in the mains voltage (rather common at the time), which both affected the high-voltage and the tube current (the latter by the heating current of the hot cathode which influenced the electron emission).

Other names for the "Coolidge tube" are "hot cathode tube", "thermionic emission X-ray tube" or "high vacuum tube" (a modern Coolidge tube has a pressure of about 10^{-6} mbar when cold, which can go up to 10^{-4} mbar when fully loaded [26].

C2. Transformer and self-rectifying Coolidge tube (one pulse), or transformer with kenotrons and Coolidge tube (one- or two-pulse)

The high-voltage was often estimated from a voltmeter reading derived from the primary. These readings were at least initially prone to considerable error when there was a change in current load (later on one tried to account for this effect). Voltage loss in the secondary circuit could occur due to an underheated cathode of a vacuum rectifying tube (kenotron, after 1926) [27]. Two-pulse systems gave a better balanced load of the mains, transformer and X-ray tube than single-pulse systems, but both systems gave similar radiation [28].

D. Inductor and Coolidge tube

Dauvillier called the combination of inductor and Coolidge tube a bad one [29]. The Coolidge tube could more or less short-circuit the inductor causing a high tube current but a low voltage which gave soft and generally few X-rays. Proper adjustment was in principle possible, but several authors seem to have noticed the anomalous low output with some puzzlement (see **ES XI** "Inductor and Coolidge tube (D)" for references).

Reference system: DC-generator and Coolidge tube

Today one finds in this class the medium and high frequency power supplies that feed a Coolidge tube. There is some ripple, among others dependent on the magnitude of the current, but from a dosimetric point of view these systems are generally considered as DC. In early radiology also several coil and transformer based DC-generators were developed [30,29,31,24], but they were hardly used in clinical practice. One version was employed by Dauvillier in a study relevant to us in which he compared the output of different generator-tube systems [30].

We might mention that in addition to the pre-1927 indicators for the kVp given in Table 1 we also found in one case the shortest wavelength of the X-rays as a measure for the kVp [93] (kVp = 12.4/ λ_{swl}, with λ_{swl} the shortest wavelength in Å with 1 Å = 0.1 nm), and in two cases an explicit value of the kVp in [20] (in its method B) and in [71].

ES IV. Maximum heat dissipation in early ion tubes

In 1896 two simple X-ray tubes were rather popular: Jackson's focus tube (or "Swan Neck") (Fig. ES2, [32]) and the Chabaud tube (Fig. ES3, [33]).

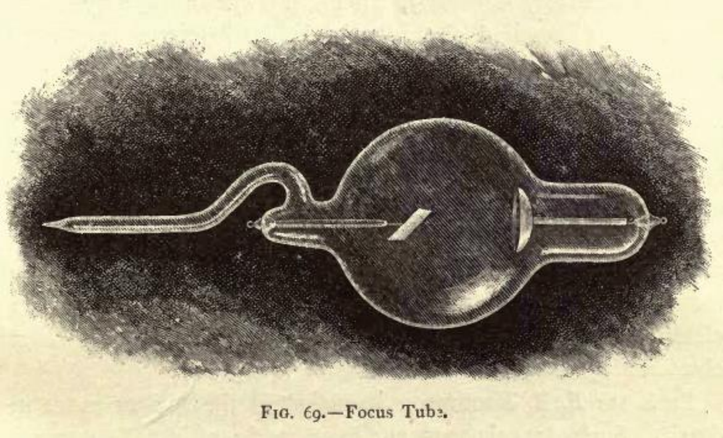

Fig. ES2 Jackson's focus tube (or the Swan Neck) from 1896, a two electrode tube with Pt-anode and focusing Al-cathode [32]

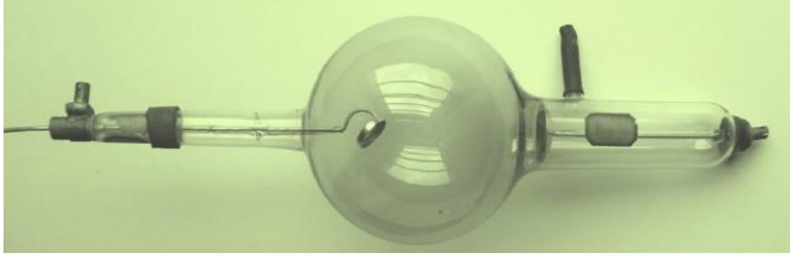

Fig. ES3 Another 1896 tube with Pt-anode, nickel anode backing and focusing Al-cathode according to Chabaud [33]

Unfortunately their early application in radiography was never adequately documented, as generally only the exposure time, the maximum spark length the coil could produce and sometimes the distance between X-ray tube and plate (or patient) were specified. However, the situation becomes more insightful if reported indications of the temperature of the anode in an operational X-ray tube might be interpreted in a quantitative way. Wright noted in 1897 "anode ... dull red-heat, but bright red-heat means too much current" [32]. Rosenfeld in 1897 was less specific as he just states "that the glowing initially is accompanied with a high intensity of X-rays, but that this diminishes" [34]. Foveau de Courmelles wrote in 1897 that during the preparation for an exposure "on constate que l'anticathode atteint le bon rouge" [35]. Gocht in 1898 stated he used his Chabaud-like tubes somewhat ruthlessly as they all got in due time a focal spot with a changed surface, giving the impression of having been molten, or the anode was

penetrated at that spot. And the focal spot was clearly glowing brightest, the rest showed a secondary glow [36]. Howgrave-Graham in 1900 “the anti-cathode is red hot at its center” [37]. Massiot and Biquard [38] stated that the Pt foil of the first tubes could stand “rouge sombre”, i.e. dark red. Ruhmer in 1904 described a Chabaud-like tube with an isolating backing of the Pt-foil that might become white hot, but stipulating that in the past a glowing anode had to be avoided [39]. Arthur in 1909 warned for not hotter than “cherry-red” [40]. Béclère (1910) implied the temperature might be made so high that with some further increase in dissipation the Pt-foil started to bend (tube current going from 1.5 mA to 2 mA) [41]. Morton in 1915 spoke of “red or nearly white heat” [42]. Bright red glowing of an anticathode is shown in a painting of early fluoroscopy of the thorax by J. Stohr (Fig. ES4) [43]. Dauvillier (1924) is the only author who specified the maximum power of a later Chabaud tube explicitly, in this case a version with the osmo-regulator according to Villard, as “hardly more than forty Watts” [29]. The better the tubes had been baked during evacuation, and probably this process was optimized over the years, the hotter they might be made during use without deteriorating the vacuum. However, “training” or “educating” the tube by using it carefully could improve its load ability also in the first years after 1895. From all reports it becomes clear, that notwithstanding the melting temperature of platinum is as high as 2041 K, it often happened that a hole was burned into the Pt-foil or that it was even heated to liquefaction. The anode could clearly become real hot.

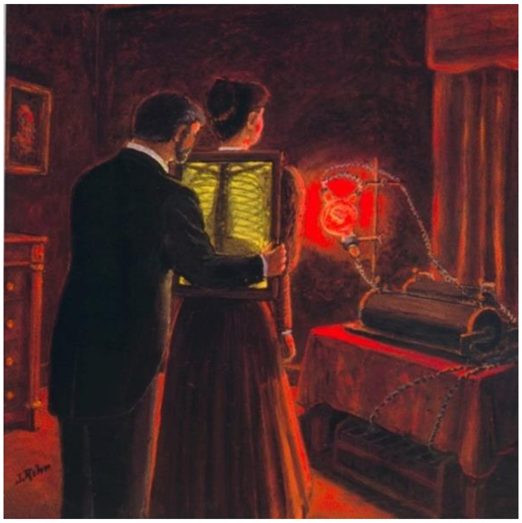

Fig. ES4 A painting by J. Rohr showing fluoroscopy of the thorax in 1896 using an ion X-ray tube with a glowing anticathode. Probably painted in 1985 after contemporary descriptions for “Histoire illustrée de la Radiologie” [43]. Courtesy of Madame Marie-José Pallardy

To limit the (very long) exposure times as much as possible, it is likely the tubes were operated close to their thermal limit. When we assume this limit to correspond with a “dark red” anode, then according to Chapman [44] the temperature of the Pt-anode must have been about 700-800°C. We take the average, 750°C. From construction details and basic physics rough estimates of the maximum thermal load and X-ray output can now be made for the two simple tubes shown in Fig. ES2 and ES3.

In an ion tube about 10% of the energy is dissipated in the cathode [45]. Another and more substantial loss comes from electrons that are scattered from the anode or anti-cathode. According to Behling [26] this energy loss is 38.6% in a Coolidge tube (smooth W anode, impact angle 90°, p 70). According to data from Reimer this value is slightly higher for a Pt target that is hit under the more glancing angle of 45°: about 43.5% [46]. These scattered electrons caused the green fluorescence of the tube's envelope and the "glass X-rays", but their main effect was heating the glass wall (one reason to make the bulb of tubes large, the other was that the "vacuum" remained longer constant upon release or capture of some gas). The total loss was thus about one half (0.1+0.9*0.435=0.49). Albers-Schönberg reported in his 1910 book [47] a heat production in the tube wall of about 60% of that in the anti-cathode, whereas the modern estimate by Reimer would amount to (0.435/0.565)*100 = 77%. Note that the X-rays generated in the W or Pt target consumed less than 1% of the total electrical energy.

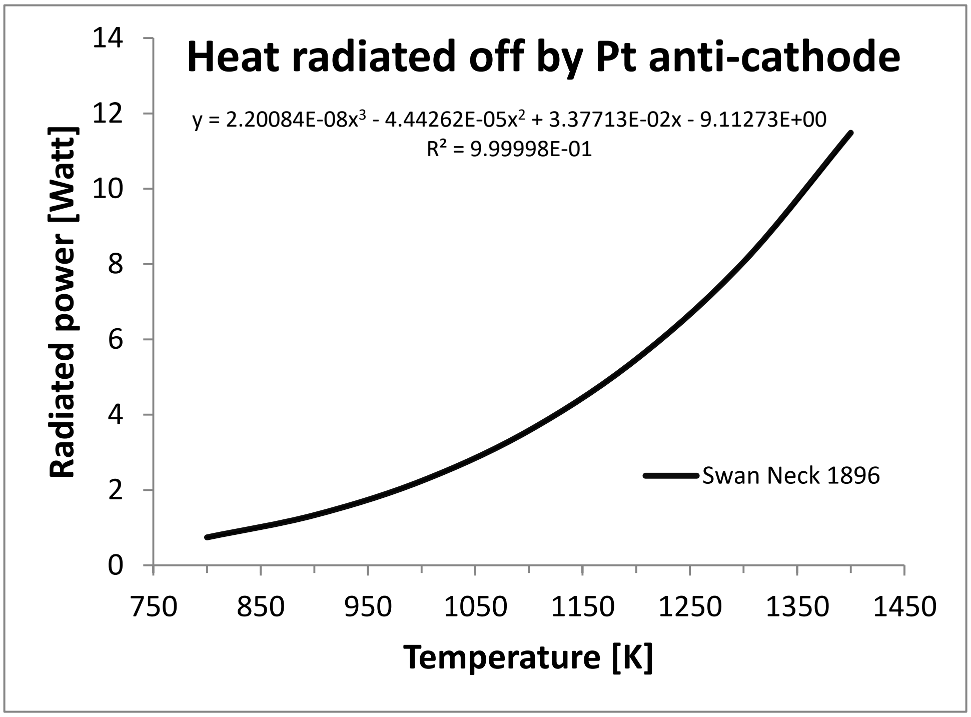

Fig. ES5 Heat radiated off by a Pt anode with an area of 1.6 cm^2 as a function of temperature (for a reading in degrees Celcius subtract 273 from the Kelvin scale). The graph applies to Jackson's focus tube (Swan Neck). For an early Chabaud tube the vertical scale has to be multiplied by about two

- Jackson's focus tube or Swan Neck tube (Fig. ES2)

In July 1896 Macintyre [48] reported in the Lancet about the successful detection of a kidney stone with X-rays using a focus tube from the firm of Newton in London. This ion-tube was equipped with a Pt-foil anode. An idea of the power that such a tube could dissipate can be obtained from the heat that could be given off by radiation (other heat losses are likely to be

small). The dimensions of the anode are about ½ inch square, corresponding to an area of 1.6 cm^2 (measured for us by Dr. Zahi Hakim who owns such a tube). The radiant emittance of a black body is given by the law of Stefan-Boltzmann: 5.67E-08 T^4 $WattK^{-4}m^{-2}$, where T stands for the temperature in Kelvin. However, the emissivity of Pt is much lower than that of a black body. Between 283 and 1773 K it increases from 0.037 to 0.191 [49]. Fig. ES5 shows the radiated power as a function of the temperature of the anode.

Assuming the anode tolerated about 750°C (1023 K), the corresponding loss by heat radiation is according to Fig. ES5 about 2.5 W. Taking the other losses into account, one finds that the electrical power could be about 2.5/0.5 ≈ 5 Watt. The load might be higher if the emissivity were higher than that of lustrous Pt, but one doesn't expect the noble metal Pt to corrode. Due to the T^4 dependence of the heat emission the allowed load is rather sensitive to the temperature seen as safe: for example, a 100 ºC increase would raise the allowed power by about 58%, a similar decrease diminish the allowed loading by 39%. The ESAK would change with nearly the same percentages.

- Chabaud tube (Fig. ES3)

This tube is not too different from the Swan Neck, except that the Pt foil in the anticathode was circular (diameter of about 20 mm) and had a nickel backing [33]. The area of the anticathode was thus about double that of the Swan Neck (3.14 cm^2 instead of 1.6 cm^2). Nickel has a higher emissivity than Pt (0.19 at 1273 K) [49] and the melting point, although lower than that of Pt, is still relatively high at 1727 K. The tube could therefore radiate about twice the heat, allowing an electrical load of about 10 W. This type of tube was made by many manufacturers, as can be seen in the book by Rønne and Nielsen [50].

Finally, note that both the Jackson and Chabaud tube have only two electrodes: a cathode and an anode. Nearly all ion tubes had three, and the X-ray producing electrode was then called the anti-cathode.

Many early inductors could handle around 100 Watt (e.g. the two antique coils in Maastricht). From electrical information scattered in the literature one gets the impression many inductors were driven with a power of this order of magnitude, e.g. Rosenfeld in 1897 [34]: 60-90 W; Foveau de Courmelles 1897 [35]: 200 W; Isenthal 1898 [51]: 120 W; Gocht 1898 [36]: 110-220 W; Walsh 1899 [52]: 80W. With an estimated electrical transformation efficiency of 0.45±0.25 for the initially used mechanical interrupters (see Table 2) the mentioned values of 5 and 10 W appear to be low. Possibly the power actually fed into the coil was lower than the values referred to, for instance when the loss in a rheostat in series with the coil was not accounted for. Otherwise the earliest inductors may have been less efficient than later versions, or our maximum-temperature estimate of the anode was simply too low. A combination of these factors is also possible. Note that Fig. ES15 (top left) also suggests that the pre-1900 dose assessments are somewhat low in comparison with later estimates. Anyhow, this discussion shows one should reckon with considerable uncertainty in these earliest dose reconstructions.

In addition we calculated the electrical loads two later tubes could sustain: a water cooled X-ray tube and a tube with a heavy anti-cathode (both introduced around 1900). The results from these calculations were used to check whether values of high voltage, current and exposure time found in publications before 1910 are compatible with the allowed loading of the anti-cathode.

Albers-Schönberg reported in 1906 that the water in the reservoir of his tube started boiling after 3¼ - 4 minutes [53]. From drawings of the tube we estimated the volume of water as around 200 ml. The power needed to cause boiling in the indicated time corresponds to about 300 Watt, the total energy about 67 kJ. Even when the substantial losses by electron scattering and cathode heating are neglected, this would still allow a current of 4 mA at a DC voltage of 75 kV (in reality thus more). Later they dissipated heat by boiling off water.

Ion tubes with an anti-cathode with a substantial mass of copper or iron allowed bursts of current higher than the water cooled tubes [50]. The all-metal anti-cathodes distributed the heat quickly by conduction, whereas during a heavy load a water cooled anti-cathode suffered from a thin layer of vapour that would isolate the water from the heated anticathode surface by the so called Leidenfrost effect. Assuming a mass of 100 g copper (or iron) and a rise in temperature of 600°C (probably a conservative guess, as a similar anode in the later Coolidge tube could be heated to a dull red-heat [54]), the total heat dissipation could be 23 kJ (for iron 31 kJ). Again neglecting losses, such tubes would still endure 10 mA at 75 kV DC during about thirty (31 resp. 36) seconds.

ES V. The kVp from a Spark Gap

The easiest way to obtain an estimate of the kVp is using the equivalent (or parallel) spark gap. The spark gap was a standard accessory on inductors. It goes back to at least 1791 [55]; see Weber's figure 9, pages 49&362. The version with point and plate was probably the most common, more so than the one with two points, as it allowed the determination of the polarity of the high voltage. Spheres of a large size were later recommended because they gave more accurate results, but they were certainly not widely used. In 1900 Béclère stressed the value of measuring the "equivalent" (or "parallel") spark gap: the maximum distance a spark could bridge between two points while the tube was functioning. Since then the device is also known as Béclère's "spintermètre" [41].

Unfortunately, there are, at least in principle, several causes why the length of the equivalent spark gap might not predict the peak voltage over the tube accurately. The voltage at the start-of-a-discharge may immediately drop after the discharge gets going. And not all individual discharges of a running system were identical (recorded waveforms all showed fluctuations in amplitude and form); the spark gap is likely to hold the larger ones for the tube voltage. Due to inductance and capacitance of the wiring, and the capacitance of the tube, the voltage over the tube might be different from that over the spark gap, especially at higher pulse frequencies or when high frequency oscillations were present. Humidity of the air was also said to play a role, and the dependence of the shape of the spark gap electrodes is always mentioned

as causing different results, but judging from Fig. 1 differences between sharp needles, blunt points, and a point and plate were not excessive. In 2011 we compared two blunt points with a blunt point and a plate: within the accuracy of our measurements the results were identical (unpublished; we measured up to a 27 cm spark gap). Luboshez also implied the same numbers for both types of spark gap [56] (their fig. 7, column 8, contains AIEE data that are also in [57]).

Curiously, Spiegler's data [58] coincide with those of others below 100 kVp but show a sudden and large step above. We excluded the deviating data in Fig. 1. An idea of the variation in the conversion of a spark gap length into a kVp is given in Table ES1 for a typical spark gap for the pelvis of 110 mm. The KfiA for the lowest kV (65 kV) is about 22% below that of the highest (80 kV) assuming 45° anode angle, 0.85 mm glass and 300 mm air.

Table ES1 kVp for 110 mm equivalent spark gap according to different authors [59-64,58,10]

	AIEE	Ullmann	US Army	Rieber	Peek	Spiegler	Schall	average	sd
kVp	80	75	76	65	78	80	69	75	6

ES VI. The kVp from penetrometer hardness

- Penetrometers of interest (Benoist, Benoist-Walter, Wehnelt and Walter)

Alternatives to the spark gap as a measure of the radiation quality are the hardness according to a penetrometer and the half value layer (HVL) of the X-rays in water. The hardness and HVL are not only affected by the peak voltage used in generating the X-rays, but also by the waveforms of the voltage and current, and by the filtration.

The first widely used penetrometer was Benoist's "Radiochromomètre" [65,2] (notice the subtle but apt difference in name with the Sabouraud-Noiré pastille, the "Chromoradiomètre"). It rested on the assumption that silver was "aradiochoique", i.e. attenuated independently of X-ray energy, whereas aluminum was very "radiochroique", i.e. caused attenuation that was strongly dependent on energy [29].

The Benoist-Walter penetrometer was a modification of the original version of Benoist in which the 12 Al steps had been reduced to 6, the thicknesses now increasing as 2.0, 2.4, 3.2, 4.4, 6.0, 8.0 mm [47]. The scale, generally indicated by BW, ran from 1 to 6 (thus a number, not an Al thickness as in Benoist's original).

The Wehnelt penetrometer was also a variation on that of Benoist: Wehnelt used an Ag foil of 0.09 mm thickness, and an Al wedge with a not-linearly increasing thickness (Fig ES6).

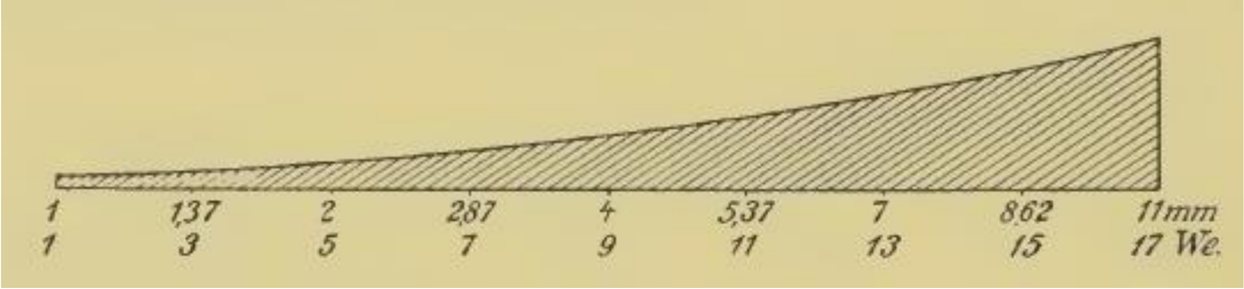

Fig. ES6 Aluminum wedge used by Wehnelt in his penetrometer (“Kryptoradiometer”). The hardness was indicated as the number (We) corresponding to the Al-thickness giving the same brightness as the (in this case) 0.09 mm Ag foil [47]

The wedge shown in Fig. ES6 could be shifted along the Ag foil until, in a window, the brightness of the Ag and Al fields appeared equal. The last penetrometer of interest to us is the one according to Walter (Fig. ES7).

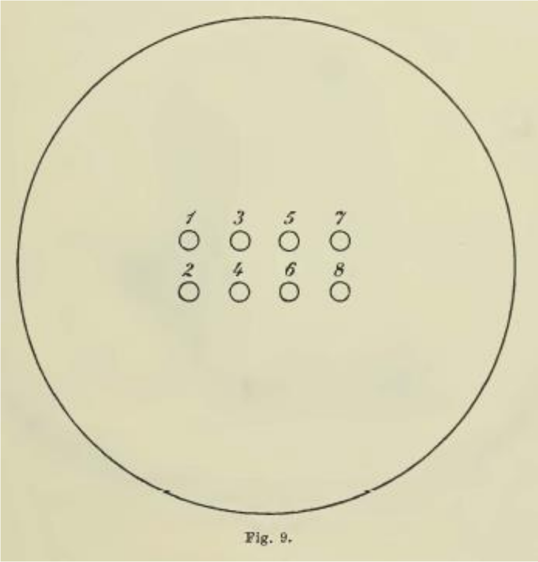

Fig. ES7 Walter penetrometer. A thick Pb plate with holes that were covered with Pt foils with geometrically increasing thicknesses of 0.005, 0.01, 0.02, 0.04, 0.08, 0.16, 0.32 and 0.64 mm, which were numbered from 1 to 8. The hardness was equal to the number of holes that was visible [47]

The Walter penetrometer does not discriminate between hardness and intensity (for X-ray systems of not too different power it worked apparently). For more information on measuring quality and quantity of X-rays in early radiology see the thesis by Gleßmer-Junike [2].

The penetrometers were generally used together with a fluoroscopic screen, most likely based on $BaPt(CN)_4.4H_2O$ or $CdWO_4$, or with some photographic material (“AgBr”). Fig. ES8 and ES9 show the results of SpekCalc simulations that might elucidate the principle of the Benoist penetrometer. Fig. ES9 includes the effect of the detector. Generally the penetrometer was placed directly before the image receptor, so scattered radiation will have affected the transmission through the various fields. As long as all measurements were done under identical circumstances this may not have caused problems.

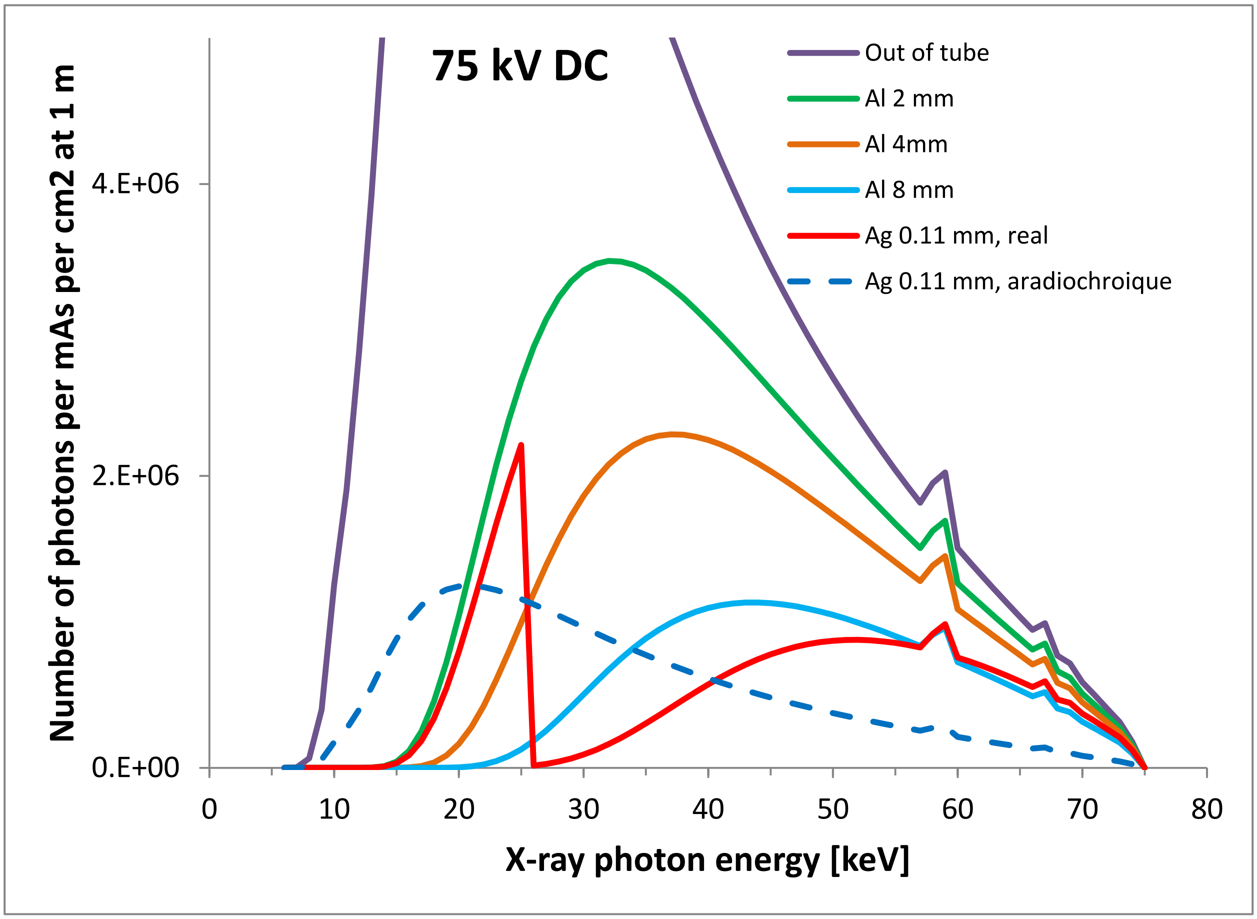

Fig. ES8 How Ag and Al affect the transmission of X-rays in a Benoist penetrometer (simulated with SpekCalc). In purple the spectrum as it comes out of an X-ray tube with a W-anode, 75 kV-DC and a 0.85 mm glass wall. The area under the curve is the total number of photons. The spectrum after attenuation by 0.11 mm Ag is shown in red. Note there is substantial transmission both at low and high photon energies, which is caused by the position of K-edge of silver (at 25.5 keV) and the choice of the thickness of the Ag-foil. The spectra in green, orange and light blue correspond to attenuation by 2, 4 and 8 mm Al. Note that Al affects the intensity mostly at low energies. Here 8 mm Al are required to get equal numbers of photons behind the Ag-foil and the Al. Hardness is therefore Benoist 8 on the basis of photon count (in practice rather on the basis of equal fluorescence). The property of Ag to have reasonable transmission at low and high energy led to the (simplified) conclusion that the attenuation of Ag was “aradiochroique”, i.e. that its attenuation was independent of photon energy. If this were true, the dashed line should hold. It does not really, but it catches part of the truth. What happens in a more realistic situation, including a fluoroscope being used in the evaluation, is shown in Fig. ES9

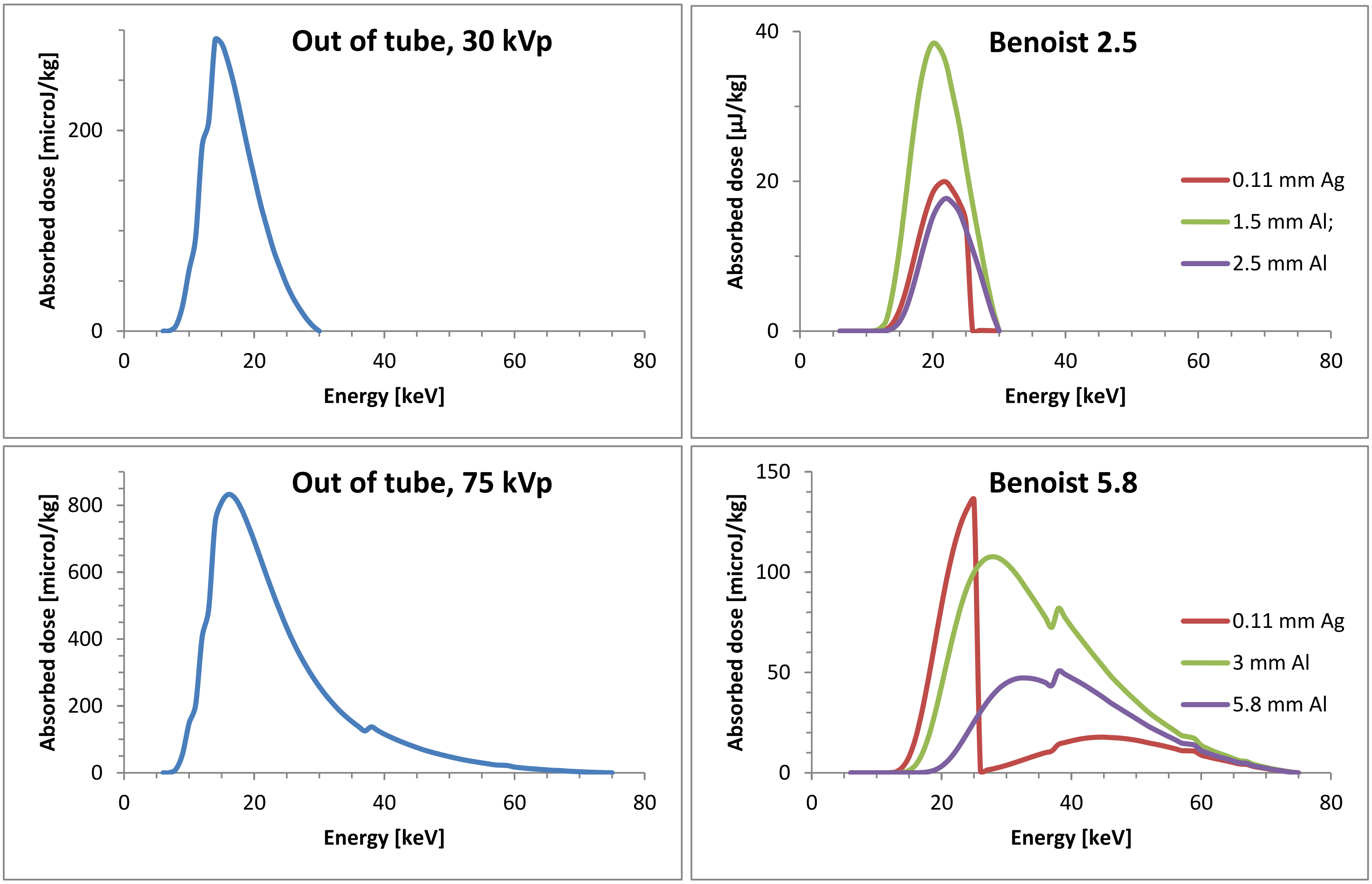

Fig. ES9 Spectrally resolved signals from a thin $BaPt(CN)_4.4H_2O$ detector in relation to a Benoist penetrometer (simulated with SpekCalc). X-ray spectra came from a tube with a wall thickness of 0.5 mm glass that was driven either with 30 kVp or 75 kVp (sine voltage and sine current pulses as from inductor and ion tube; 1 mAs). Detector at 1 m from the X-ray focus, narrow beam geometry. Left the emitted spectra, right those behind 0.11 mm Ag (red) and behind Al (two thicknesses, green and purple). Areas of red and purple graphs are equal; green spectra only to illustrate effect of less than "Benoist attenuation". At low kVp Al is an efficient attenuator, i.e. little is needed to reduce the transmission to match the transmission through Ag. At high kVp Al is not so effective and a lot is needed to match the attenuation by Ag. All edges and bumps in the spectra are due to physical phenomena related to L- and K-electron states in the heavier elements Ag, Ba, W and Pt

- Hardness → spark gap → kVp

In the literature we found several publications giving a relation between hardness and the corresponding equivalent spark gap. The data are shown in Fig. ES10, together with fits of polynomials (for references see Table ES2). Most of the data were from inductors, a few from transformer systems, but we had too few sources to separate them.

The hardness found in a description of a clinical exposure was converted into a spark gap using the fits shown in Fig. ES10 (for completeness Fig. 3 was re-included). The spark gap in turn was used to calculate the kVp (see Fig. 1).

In Table ES2 we show the kVp's, for a radiation quality recommended by Albers-Schönberg [47] for radiography of the pelvis, obtained along this spark gap route. Albers-Schönberg specified this optimal hardness on different scales.

Table ES2 kVp from hardness suitable for pelvis [47] using spark gap route [4,47,56,66-68]

Penetrometer	Hard-ness	Kien-böck	Albers-Schönberg	Lubo-shez	Hacken-bruch	Muir	Christen	average	sd	n
		kVp	kVp	kVp	kVp	kVp	kVp	kVp	kVp	
Benoist	5	88	76	66	64	83	70	74	10	6
Benoist-Walter	4.5	89	75	67	66	-	75	74	9	5
Wehnelt	7.3	72	74	68	66	81	74	72	5	6
Walter	6	-	75	64	57	-	69	66	7	4

The observed differences may at least partly be caused by differences in voltage and current waveforms and filtration. Because the Benoist and Benoist-Walter scales are directly related we can check the consistency of their calibrations. E.g. 5 B corresponds approximately with 4.5 BW (both 5 mm Al). The two corresponding kVp's are both 74 kV, in good agreement. However, we don't know whether the authors performed independent measurements or converted one set of data into the other.

- Hardness → HVL → kVp

Although the HVL was hardly used in clinical radiography to specify the hardness of the X-rays used, data giving the relation between penetrometer hardness and HVL were rather numerous (Fig. ES11). This suggests at least in principle another route for the conversion of a penetrometer reading into a kVp, namely by using the HVL as an intermediate (instead of the spark gap). For this we also need a relation between HVL and kVp, which was provisorily derived using SpekCalc. We calculated the HVL in water for 25, 40, 60, 80, 100 and 125 kV(p), using a pulse with both voltage and current in the shape of a sine lobe. We will see hereafter that the latter may be considered as representative for the average pulse from an inductor used with an ion tube. Table ES3 illustrates an example of conversion along this line.

Table ES3 kVp from hardness suitable for pelvis [47] using HVL data [69,66,68,47,70]

Penetrometer	Hard-ness	Blumen-thal	Hacken-bruch	Christen	Albers-Schönberg	Guille-minot	average	sd	n
		kVp	kVp	kVp	kVp	kVp	kVp	kVp	
Benoist	5	37	38	76	72	131	71	38	5
Ben.-Walter	4.5	77	49	90	72		72	17	4
Wehnelt	7.3	49	43	84	73		62	20	4
Walter	6	44	44	82	72		60	19	4

Note the extreme variations in this Table (e.g. in comparison with Table ES2). SpekCalc implicitly assumes “narrow beam” geometry, whereas for instance Christen did not use an air gap between his “water” absorber and detector, resulting in “wide beam” geometry. HVL measurements, contrary to our simulations, were thus affected by scattered radiation. Our not including scatter in the calculations most likely caused some systematic shift in HVLs, but will not have caused the large variability seen in Table ES3. This large spread must be due to inaccuracies in the reported measurements. We therefore forget about the ‘hardness-HVL-kVp’ path and will limit ourselves to the spark gap route.

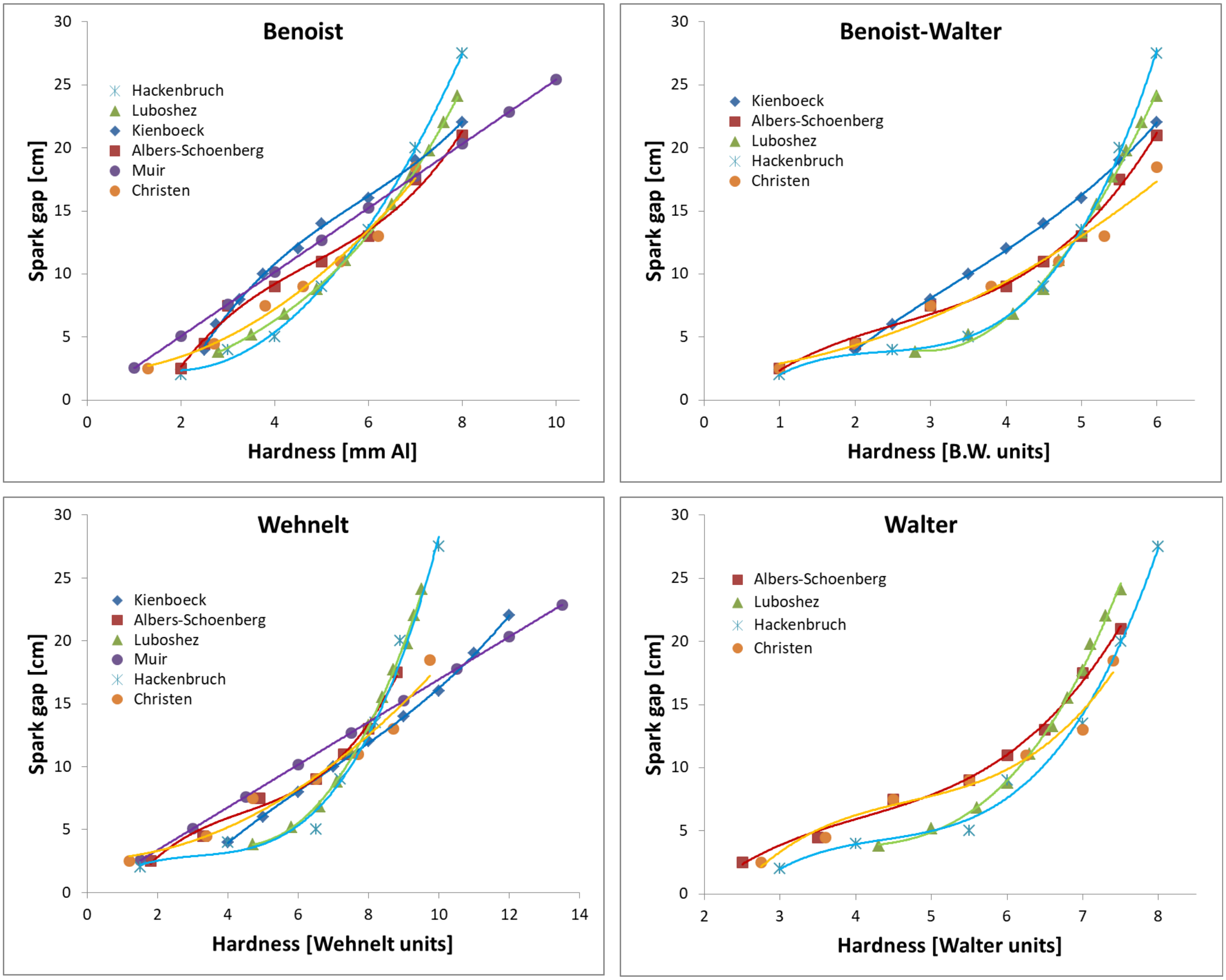

Fig. ES10 Spark gap length as a function of the hardness according to the penetrometer scales of Benoist, Benoist-Walter, Wehnelt and Walter (for completeness we re-included Fig. 3). For Benoist one mostly reads B followed by a number without the unit "mm Al" [4,47,56,66-68]

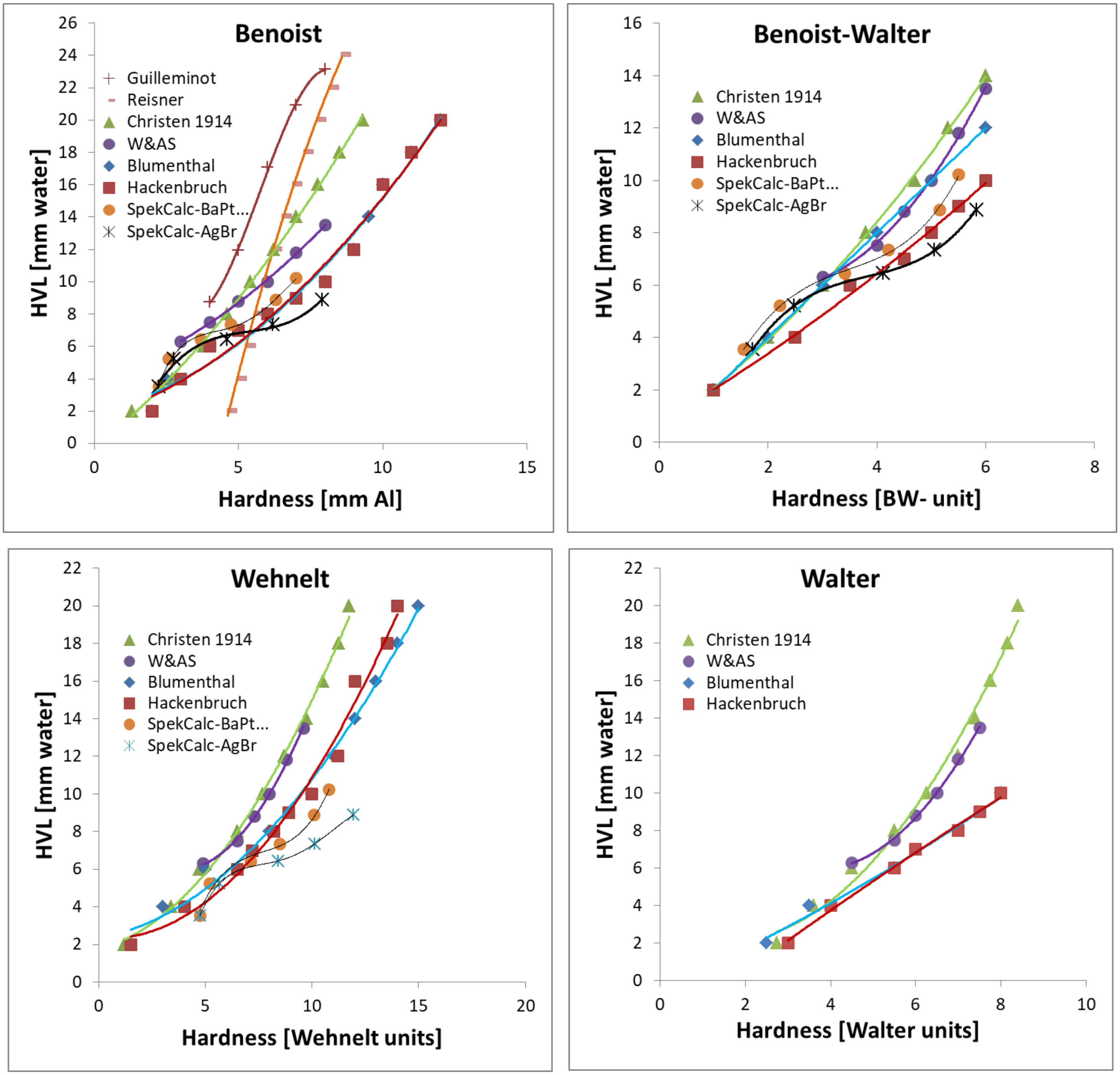

Fig. ES11 HVL as a function of the hardness according to the penetrometer scales according to Benoist, Benoist-Walter, Wehnelt and Walter [69,66,68,47,70]

- Simulation of Benoist and Wehnelt penetrometer

Even though SpekCalc implicitly assumes narrow beam geometry and penetrometers were used in wide beam geometry, we thought that simulation might illustrate the effect of the various individual radiation quality parameters and possibly also reveal an effect of the type of detector used in determining the hardness (fluoroscopic screen or photographic plate). Such an effect was not reported as far we know. Using SpekCalc we simulated therefore the two-element penetrometers including detectors. As the results would be of most interest for data from inductor-ion tube systems, we used sine shaped voltage and current pulses along with DC excitation. At 20, 30, 40, 50, 75, 100 and 125 kVp we simulated:

1. the photon transmission through an Ag foil (0.11 mm for Benoist, 0.09 mm for Wehnelt)
2. the photon transmission through several thicknesses of Al (up to 10 mm), and
3. the energy fluence, as well as the absorbed dose in thin layers of AgBr, $BaPt(CN)_4.4H_2O$ and air behind all absorbers.

Linear interpolation of the Al-data was used to find the Al thickness that transmitted the same signal as the Ag foil. The energy fluence may be seen as the signal that would be obtained if all X-ray energy in the beam contributed to the signal.

In the calculations we assumed a 45 degree anode angle and 250 mm air between tube and detector, and we considered both 0.5 mm and 1.0 mm glass thickness of the tube. The results are shown in Fig. ES12 for the Benoist penetrometer; the results for Wehnelt's device were qualitatively identical (not shown).

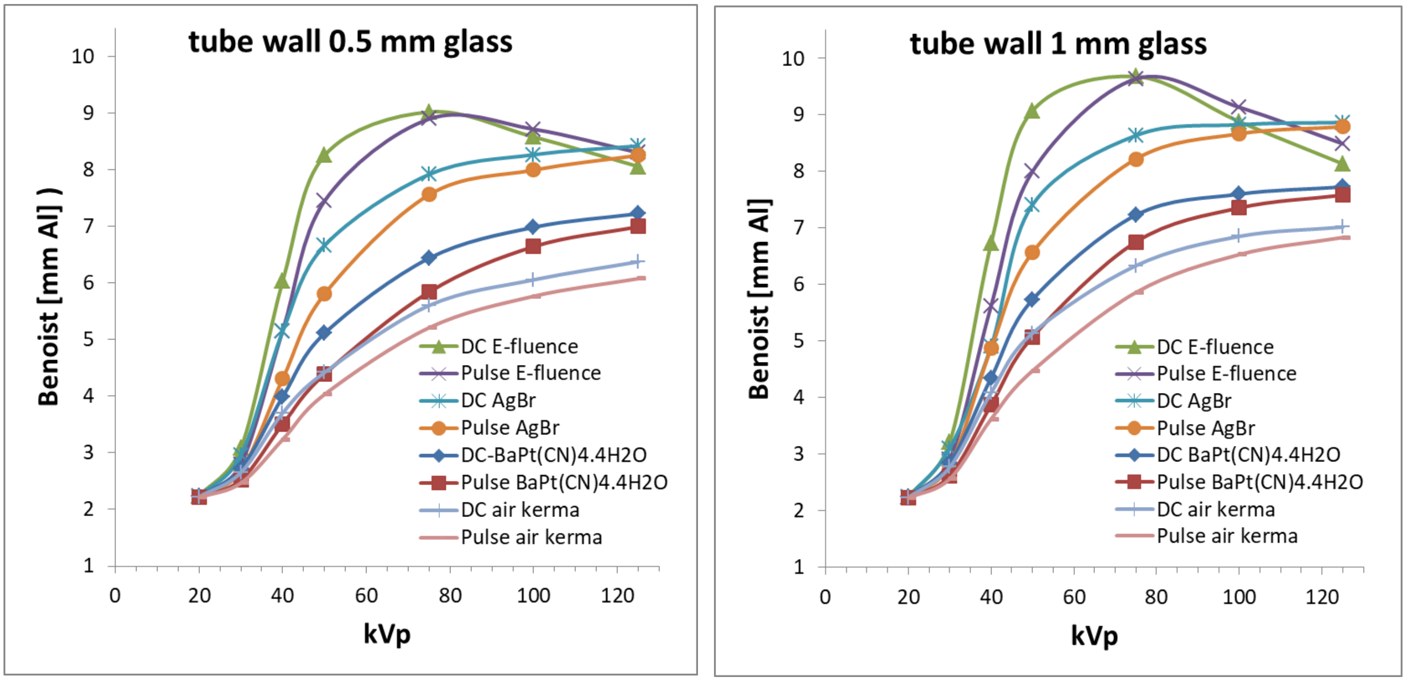

Fig. ES12 Benoist hardness as a function of DC and pulsed ("sine voltage, sine current") high voltage. Hardness derived from transmitted energy fluence and signals from thin detectors of AgBr, $BaPt(CN)_4.4H_2O$ and air. Left filtration by 0.5 mm glass, right 1 mm, and in both cases 250 mm air. Based on simulations using SpekCalc (W-anode)

The curves show that the penetrometer does indeed reflect the effects of kVp, type of power supply (DC or pulsed) and filtration (glass thickness). This implies that our conversion of a hardness found in the literature into a kVp will only be strictly correct if the filtration and waveforms of voltage and current were fully identical for all systems. This will not have been the case. There is also a clear dependence on the detector used in gauging the penetrometer, and that is as to be expected. The X-rays, that would have contributed most to sensitizing the AgBr, had already been absorbed by the Ag-filter; the resulting low AgBr signal (free silver after development) could only be matched by a thicker layer of attenuating Al. The only other source we saw hinting at a possible difference in the observed hardness caused by the detection method is in a brochure from Reiniger, Gebbert und Schall, where they explicitly state that a given Benoist hardness was measured with a fluoroscopic screen [71]. The simulations further corroborate the observation of Dauvillier that the Benoist penetrometer is only useful up to about 80 kVp [29]. The same holds for Wehnelt's device.

Again, remember that our simulations apply to narrow beam geometry and a very thin detector. Thus our simulations give an idea of the behavior of the Benoist and Wehnelt penetrometers, but are not suited for quantitative assessment of old data.

ES VII. Electrical information on primary and secondary circuit of ion tube systems

- Current and power in secondary circuit

After 1895 the amount of information on exposures with inductor - ion tube systems gradually increased. Note that Snook systems only appeared after 1907. Information might be given on the type of primary power supply (batteries, DC-mains), the voltage of that supply (V_{prim}), the average primary current ($I_{prim,av}$), the maximum or the equivalent spark gap of the secondary circuit that fed the X-ray tube and the exposure time. Before 1900, and still some time thereafter, the tube current would be missing but it can be derived if the electrical transformation efficiency (α) is known.

Using the parameters just introduced, the electrical power in the secondary available for the X-ray tube can be written as $P_{sec} = \alpha * P_{prim} = \alpha * V_{prim} * I_{prim,av}$. Using the kVp, from the spark gap, the missing tube current can be calculated. We assume again that the pulse generated by the interrupter-inductor system can be approximated by a sine, both for the voltage and current. Call I_0 the maximum current (amplitude) in the secondary circuit, T the duration of a single pulse and t the dead time in-between two pulses as exists when using an inductor with an ion tube. The frequency of the pulses is then $\nu = 1/(T + t)$. The average current, within the pulse duration T of a sine shaped current, is $I_{av\text{-}pulse} = (2/\pi)\, I_0$, on average over a whole period (T + t) this becomes $I_{av} = (2/\pi)\, I_0 * \{T/(T + t)\}$, i.e. $I_0 = (\pi/2) * I_{av} * (T + t)/T$.

Let kVp be the peak voltage, then the power expended in the secondary amounts to

$$P_{sec} = \nu \int_0^T kV_p\, I_0\, sin^2\left(\frac{\pi}{T} t\right) dt = \frac{1}{2}\, \nu\, kV_p\, I_0\, T = \frac{1}{2}\, \nu\, kV_p\, \frac{\pi}{2}\, I_{av}\, \frac{T+t}{T}\, T = \frac{\pi}{4}\, kV_p\, I_{av}$$

From this one finds for the time averaged current.

$$I_{av} = \frac{4}{\pi}\frac{P_{sec}}{kV_p} = 1.2732\,\frac{P_{sec}}{kV_p} = 1.2732\,\frac{\alpha\, V_{prim}\, I_{prim}}{kV_p}$$

We now have the ingredients for the calculation of the X-ray output if the primary voltage, primary current, the system's electrical transformation efficiency and the secondary kVp (via spark gap or hardness) are known. In fact we only need a very rough estimate of the kVp because for a fixed P_{sec} and unfiltered X-rays (e.g. inherent filter 0.85 mm glass and 250 mm air) the effect of any change in kVp on the KfiA is largely compensated by the concomitant change in tube current ("mA"). For instance, changing the high voltage from 60 to 130 kV only causes an increase in air kerma of 9%, as can easily be checked using Fig. ES13 (left) (the change would even have been zero if the linear functions went through the origin). In other words, knowing P_{sec} for an early system suffices to make an estimate of the system's air kerma, as an estimate of the kVp can always be obtained from the clinical application at hand. A rough estimate of P_{sec} can also be obtained from the thermal characteristics of the anode/anticathode as we have seen (**ES IV**). The situation is quite different if the X-rays are attenuated by 200 mm water ("a patient"): for a fixed P_{sec} the KfiA would already increase by 100% when going from 60 to 130 kV (from data for Fig. ES13, right).

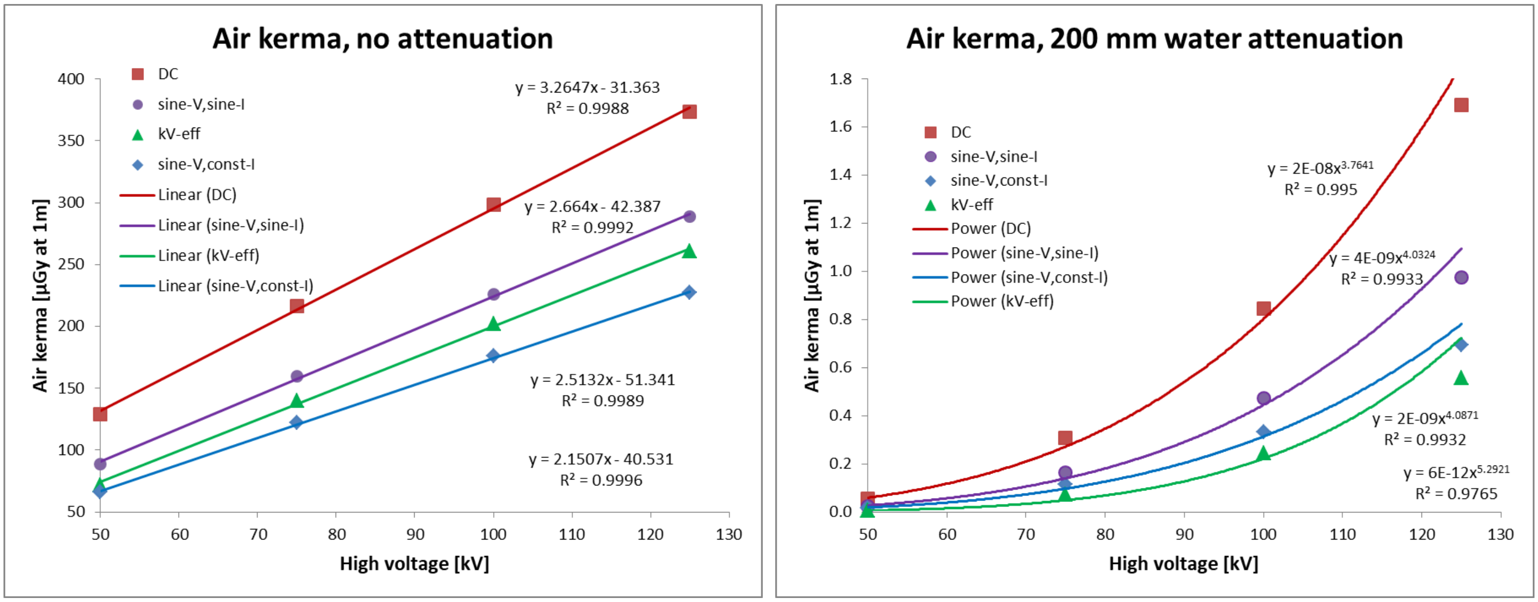

Fig. ES13 Air kerma from tube with W anode at 45º; 1 mAs. Filter 0.85 mm glass (0.76 mm Al-eq.) and 250 mm air (left). Right: Additional attenuation by 200 mm water. SpekCalc simulations

- Electrical transformation efficiency (α) of inductor-interrupter system

The electrical transformation efficiency depends on the inductor and interrupter, both by construction and adjustment. An inductor is an open core transformer that has a considerable

lower efficiency than the normal closed core transformer. Wertheim-Salomonson reported in 1920 a (transformer function only, no interrupter) efficiency of 62±4% (data from two different coils; most likely the spread was much larger for all systems in the field).

The interrupters, required for the commonly used DC primary power supplies, had their own losses, often in the form of arcing, heating or electrolysis. Wertheim-Salomonson reported in 1905 an efficiency of 56 – 59% for a coil with a mechanical interrupter, and referring to "before", 35-40% [72], whereas Armagnac [73] never found an efficiency higher than 40% and others even spoke of 20% [51]. Probably there was some increase in efficiency over the years, but proper adjustment must have been important at all times.

For an inductor with mercury interrupter Wertheim-Salomonson gives in other articles (also from 1905) efficiencies of 48 – 55%, [74,75], in 1920 for a small 12" coil 35 – 54% [76].

Concerning Wehnelt interrupters all investigators agree on a low efficiency. Wertheim-Salomonson reported some of the higher efficiencies, 27 – 32% [75]. Jaugeas reported 33% or less [77,78]. In a thesis Allen reported in 1899 a low efficiency of 9% [79]. Armagnat wrote that sometimes more than 80% of all energy used is dissipated in the Wehnelt interrupter [73], Brunel 70% [80], implying that the efficiency of the whole system was possibly as low as 12 – 19% (taking into account the efficiency of the coil itself, Wertheim-Salomonson's 62%). In a 1912 product catalogue of Reiniger, Gebbert & Schall [81] one reads that a system with a Wehnelt interrupter needs about 2.5 times as much current as a system with a mercury interrupter. Turner [82] and Arthur [40] even give a ratio of 10 to 3. Biddle [83] compares the Wehnelt interrupter with a mechanical one, informing that 2.5 A through the latter gives the same output as 12-14 A with the Wehnelt (3 inch equivalent spark gap, 1.5 mA tube current). Using the Wertheim-Salomonson estimate of 35 – 54% for the efficiency of a mercury interrupter system, that of a Wehnelt system would according to RGS be 14 – 21%, and according to Turner and Arthur 11 – 16%. Biddle's data imply an efficiency of about 9%, assuming an efficiency of 45% for the mechanical interrupter (45*2.5/13).

Table 2 (main text) gives what we think are reasonable estimates of the electrical efficiency α and associated uncertainties (the latter in fact educated guesses). The efficiencies are compatible with Janus' 1909-statement concerning the various systems "Wirkungsgrad: etwa 20 bis 60%" [84]. Jaugeas in 1910 [77] stated "Even with the best and newest coils the output is seldom more than 55 per cent of the total electric energy received."

In some of the earliest publications one gets the impression that the voltage referred to was that of the source, e.g. the set of batteries used. In case there was a resistance in series to adjust the power supplied to the inductor, the actual voltage over the inductor would be smaller than the value specified. We assumed that in these cases the electrical loss in the resistance was small, as the adjustment required after a proper choice of voltage (by the number of battery cells) would be limited. The later mercury and electrolytic interrupters, which replaced the initial mechanical devices, often provided by their construction control over the current (and power).

ES VIII. Anticathode/anode material

In some of the very first experiments the glass wall opposite the cathode was the location where impinging electrons generated X-rays. Very soon the standard X-ray tube was one with a platinum anti-cathode (effectively the anode) and a concave (electron focusing) cathode made of Al. This form had already been designed by Crookes, long before Röntgen's discovery to demonstrate the heating effect of "cathode-rays" (electrons) [85]. In addition to platinum (Pt, atomic number Z=78) also iridium (Ir, Z=77), Ir-Pt alloys and tantalum (Ta, Z=73) found limited use (see patent Siemens&Halske from 1904 [86,50]). Between 1908 and 1910 Siemens succeeded in making sintered tungsten (W, Z=74; patent on use of W already issued in 1905 [87]) and from 1912 onwards it was actually used as anode material in ion tubes, also by licensees [50]. Coolidge in 1913 also used tungsten in his high-vacuum thermionic-electron-emission X-ray tube, both for the hot-cathode filament and the anode [88]. The production of "Bremssstrahlung" is proportional with Z, and as at diagnostic energies the contribution of K-X-rays is a few percent at most, the total output of an X-ray tube scales with Z. With the introduction of the (Goetze) line focus [89] (patent filed in 1918) in a Müller tube in 1922 the anode angle was decreased, both for gas and Coolidge tubes. Today values are between about 7º and 20º; for general radiography the angle may be in the range 12º - 16º.

ES IX. Filtration (glass envelope of X-ray tube, additional filter and air)

After being emitted by the anode, the X-rays were initially only filtered by the glass envelope and the air between tube and patient. To reduce the "waste" of X-rays, the envelope was made as thin as compatible with mechanical strength (there was a risk of implosion).

We assumed that the much used sodium glass had the composition of "Thüringer Waldglas" [90]. Using SpekCalc simulations we found that for voltages around 75 kV the attenuation of 1 mm of that glass corresponds to that of 0.90 mm Al. The thickness of this glass varied with the diameter of the tube: for smaller bulbs (12-15 cm) the thickness was commonly about 0.4-0.8 mm, for larger bulbs (about 15 to 20 cm) 0.8-1.0 mm. Sources are Walter 1907 [91], Christen 1913 [92], Fritz 1922 [93], Neeff 1930 [12], Schall&Son 1914 [94], Albers-Schönberg 1910 [47] and Rønne 1986 [50]. The only deviating value, 2 mm, was reported by Kotre et al. in their retrospective study from 2006 [95]. Wucherpfennig measured in 1928 the half-value layer of 35 X-ray tubes, probably all Coolidge tubes [96]. At 82 kVp on a two-pulse system the HVL in Al varied between 0.23 mm and 0.88 mm, which corresponds to wall thicknesses of 0.1 to 0.7 mm Al eq. (SpekCalc). Some tubes had extremely transparent windows for skin therapy, but such tubes could also be applied in diagnostics. In our post-1900 computations we assumed a wall thickness of 0.85±0.15 mm, before 1900 we took 0.6 mm.

The thickness of the wall had a large impact on the air kerma, and for thin tube envelopes even the air between the tube and patient had a noticeable effect. Setting the KfiA for 75 kV DC and 1 mm glass and 350 mm air at 100%, the KfiA for 0.4 mm glass and 350 mm air would be

204%, and without air the numbers are 103% and 219%. For diagnostic radiography generally no additional filters were applied, as one did not want to lose intensity, even though X-rays of low energy added little to the image. Hirsch still writes in 1920 [20] “The interposition of an aluminum filter, though essential in fluoroscopy, is not necessary in radiography“. Additional filtering was sometimes recommended, starting in the twenties, but even in 1942 Smedal reported that from five X-ray systems only two had 0.5 mm Al added filtration. Harder radiation could in principle be used after the introduction of the anti-scatter grid that reduced “scatter fog”. Bucky introduced his grid in 1913 [97], improvements came from Potter in 1920 [98], [99]. Grigg gives a very nice “digression” (as he calls it) on “Bucky-Potter” [100]. Metal deposits on the tube’s glass wall, which might have been caused by extensive use, had a small effect: the thickest W deposit Coolidge found on returned tubes measured 1 μm [101], which would cause 12% attenuation at 75 kV DC (SpekCalc, 0.85 mm glass, 250 mm air and a 45° anode).

ES X. X-ray efficiency of old pulsed systems compared to a DC system

We related the output of a pulsed X-ray system to a DC system by an efficiency factor. Alternatively, one might also derive a DC-kV ($kV_{equivalent}$) giving the same output as the pulsed system at kVp. Such an approach would be reminiscent of the often used kV-eff approach for rectified single phase - one or two pulse - systems. However, for the first approach, but not for the second, early experimental data do exist, so the efficiency approach appeared the logical choice. In the literature we not only found comparisons of pulsed systems with their DC counterparts, but also between various pulsed systems mutually. We only looked at comparisons based on measurements of (nearly) unattenuated output (KfiA), not at transmission measurements through patients or absorbers. In these comparisons different kinds of detector materials might have been used: we noticed among others air, CH_3Br and CH_3I in ionization chambers, AgBr in photographic emulsions, $CaWO_4$ in intensifying, $BaPt(CN)_4.4H_2O$, $CdWO_4$ and ZnS in fluoroscopic screens. To check if the reported outcome of a comparison of two X-ray systems was dependent on the detection method, we simulated with SpekCalc the absorbed dose in a number of these materials at 50 and 100 kVp for DC voltage and the rectified form of normal AC-voltage with constant current; the latter is the pulse form that deviates most from DC in efficiency of X-ray production as we will see. Table ES3 shows the pulsed/DC ratios, illustrating that it hardly matters how system comparisons had been performed in the past: for our purpose the ratios can be considered as identical as the maximum difference is within 8% (the sd is only 3 – 4%). (The difference between AgBr and BaPt-cyanide was large in the penetrometers because there was heavy filtering, especially by the Ag filter).

Table ES3 Ratio absorbed dose pulsed[a]/DC in different detector material

kV	Air	CH_3Br	CH_3I	AgBr	$CaWO_4$	$BaPt(CN)_4.4H_2O$	Average	Stand. dev.
50	0.512	0.486	0.475	0.469	0.497	0.491	0.488	0.015
100	0.458	0.575	0.540	0.554	0.579	0.568	0.567	0.018

[a] Pulse form of rectified AC over Coolidge tube in current saturation

Quite a number of authors also provided voltage and current waveforms of the electrical pulses that excited the X-ray tube. The forms of these curves often give already a rather good impression of the X-ray efficiency of a system. A few of them were digitized with the “antiplotter” program Graph Grabber [18]. These waveforms were used as input for SpekCalc to calculate the kerma free in air. As before, the air kerma for a DC voltage was always calculated as well. The average tube current was kept constant in these comparisons.

We simulated also a number of idealized waveforms (Fig. ES14):

1. sine voltage and constant current as would ideally apply to a transformer with rectifiers exciting a Coolidge tube under the condition of saturated tube current (i.e. current equal to the total thermal electron emission of the hot cathode). Applies also to transformer with self-rectifying Coolidge tube.
2. a sine voltage together with a sine current
3. a triangular shaped voltage and triangular current
4. sometimes the rising or trailing current flank could be in a region where the voltage was high. This was simulated with a voltage that is half triangular and half constant, while the current was of triangular shape.

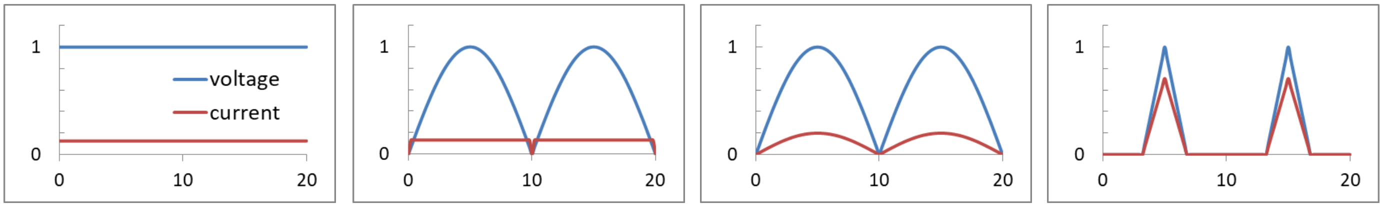

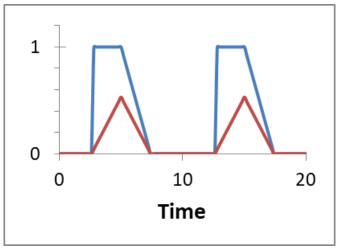

Fig. ES14 Idealized waveforms of voltage and current pulses used to excite an X-ray tube. Top: from left to right: DC, sine shaped voltage and constant current, both voltage and current sine shaped and triangular (“spike”) voltage and current. Bottom: also triangular current but initial half of voltage is high then linearly falling. Average current is in all cases identical. Note sine lobes may also be disjoint (i.e. $t \neq 0$, see **ES VII).**

The results of the comparison between systems excited with these pulses and a DC system are shown in Table ES4. The pulse forms observed on inductor-ion tube systems varied; the forms in columns 3 – 5 are idealizations of what was reported and seen in graphs in the literature.

Table ES4 X-ray efficiency, ε, of different idealized electrical pulse forms

Form of:<br>- voltage	sine	sine	triangular	half triangular,<br>half constant
- current	constant	sine	triangular	triangular
X-ray system	Transformer -Coolidge	Inductor-ion tube<br>Snook-Ion tube	Inductor-ion tube	Inductor-ion tube
50 kVp	0.510	0.688	0.520	0.760
100kVp	0.589	0.757	0.621	0.811
average	0.55	0.72	0.57	0.79

The results found in the literature matched reasonably well with those from simulations of the idealized pulses. The combined information was given in Table 3 in the main text. The Snook-Coolidge system was in practice no more efficient than the transformer-Coolidge combination. Both have an X-ray efficiency close to that of a system excited with a sine voltage and constant current. The higher efficiency for the transformer&Snook ion tube combination is due to the fact that the current increases with the voltage ('sine-sine'). The efficiency of the inductor-ion tube was close to that of the Transf&Snook-ion tube ('sine-sine'), but appeared to be more variable. In **ES XI** we give a very condensed (but still long) overview with information on how we came to the ε-values in Table 3.

A few additional remarks are in order. For transformer-Coolidge systems one often sees the kV-eff approach, i.e one assumes that the DC voltage kV-eff = kVp/√2 gives the same output as a rectified sine voltage with a peak of kVp. Simulations with SpekCalc show that this is not far beside the truth for the kerma free in air for unfiltered X-rays (Fig. ES13 left). The kV-eff approach gives an estimate that is too high by 9 to 15% over 50 to 125 kV (i.e. compared to true 'sine-V, const-I'). Behind 200 mm water the KfiA is underestimated and the discordance larger, the underestimation decreasing from 74% to 20%. Note also the difference in the functions describing the dependence on kV: a linear function in case of minimal filtration versus (roughly) a power function behind a patient. The latter is well-known from textbooks describing the effect of kV-change on exposure.

As stated in **ES III**, we only found one author, Willey [45], who reported the energy loss in the cathode of an ion tube. He found a loss of about 10%, later corroborated by Dauvillier [29]. This limits the maximum X-ray efficiency of any system that uses an ion tube to 90% (note that the energy for the hot cathode in a Coolidge tube is not a loss in the secondary as it comes from a separate electrical power supply).

Many investigators reported anomalously low X-ray yield from the inductor-Coolidge tube combination. Unlike a transformer an inductor is preloaded with electrical and magnetic energy and cannot adjust itself to the load. Given the limited power of most inductors and the increasing power rating of Coolidge tubes, the inductor could easily be overloaded leading to a low-voltage current and soft X-rays.

Finally, in case efficiency relations were reported between two different pulsed systems, we used this information within both groups (on the group's average), which introduces (some)

cyclic dependence. However, omitting this information hardly affected the efficiencies, showing consistency within the data.

ES XI. Summary of data and literature used in determining X-ray efficiency

The following is an overview of information on X-ray efficiencies. Reported waveforms were sometimes used in SpekCalc simulations. The original article may be needed to understand our choice; some arbitrariness in decisions was often not to be avoided. Estimates of efficiency ε are given in red. Numbers within { } are the result of an efficiency specification between two pulsed systems. The average efficiency for the systems considered was given in Table 3. In the following "Fig." refers to figures in the cited literature, not in our text. Likewise, "P" stands for page in the reference.

DC voltage and Coolidge tube

This is our reference system; by definition: ε = 1. We used the appropriate anode angle in SpekCalc and we corrected for the anode material if not tungsten.

A. Inductor and ion tube (often also called gas tube)

1. Willey reports on P 257 that about 10% of the energy dissipated in the ion tube is used for the generation of the cathode rays. Scattered cathode rays same as in Coolidge, except in ion tube they also hit wall, not so in the Coolidge tube. Maximum efficiency is 0.9 wrt our reference. P20, 133[45,29]. **ε** =0.9
2. Dauvillier shows that voltage and current are, forgetting spikes, of an approximately triangular shape (with capacitor of Fizeau) [30], Fig. 17. **ε** =0.57 (from SpekCalc). Without capacitor a current peak (possibly consisting of spikes) from mid to trailing edge of voltage peak, Fig.19. **ε** =0.79 (from SpekCalc).
3. Dauvillier in book ([29], p 20&133) says that he "confirms" a loss of 20% (in cathode he presumes, he seems to cite Willey wrongly, but only his finding matters). **ε** =0.8
4. Glocker: Inductor pulses can be very short, leading to a too high average current reading from a mA-meter ("Drehspulenmilliamperemeter", to be adjusted with 0.72 when compared with transformer [102], P 427. Others believe in correct reading. Not used.
5. Owen shows secondary voltage and current waveforms for two coils. The voltage spikes are identical, but the current forms are not (one spike, other spike with tail). Efficiency compared to DC rather low! [103]. See his Figs. 3 and 14&15. Fig. 14 shows a very large current tail, like inductor on Coolidge in unfortunate case. **ε** =0.57 for Coil 1 (from SpekCalc); **ε** <0.57 for Coil 2, say **ε** =0.5.
6. Crowther (1924) finds triangular shapes for voltage and current for gas tube with mercury break and Simon electrolytic break [104]. But in Fig. 14 V-curve wider than I-curve; they are similar in Fig.8. **ε** =0.57 and **ε** =0.79 (SpekCalc).
7. Owen reports triangular pulses [105]. **ε** =0.57 (from SpekCalc)

8. Duddell does also, both for V and I. He considers both real rectangular pulses as well as a rectangle-with-a-exponentially-decaying-hypothenusa [106]. But V-registration is dubious. Not used.
9. Codd finds a 20% higher efficiency compared to transformer-Coolidge set [107].
   ε =1.2*C2 {= 0.67}
10. Fritz: same Photographs of Snook-Transformer-Ion tube as of Inductor-ion tube [93].
    ε =1.0*B {= 0.72}
11. Wertheim Salomonson: 25% higher output for inductor-ion tube than for Snook-transformer-ion tube [108]. **ε** =1.25*B {= 0.89}
12. Taylor-Jones in his book shows strongly oscillating voltage, meaning lots of soft X-rays [109]. Not used.

B. Transformer or Snook and ion tube

1. Dauvillier reports that a transformer on a gas tube gives a factor of 1.7 more output than that transformer does on a Coolidge tube, P133 [29]. **ε** =1.7*C2 {= 0.95}
2. Dauvillier: transformer on a hard gas tube gives only 20% (loss in cathode in gas tube) less output than a DC-generator on a Coolidge tube (p 133). See also Fig. 57A [29]. **ε** =0.8.
3. Dauvillier shows in [30] voltage and current much alike transformer on Coolidge (Figs 21&22), except that current stops at V>>0 in case of ion tube, and at V=0 for Coolidge. For current see also Fig. 24 & 25 [30]. SpekCalc simulation of digitized Fig. 22:
   ε = 0.9*0.9=0.81.
4. Glocker reports at the identical output of transformer and inductor on ion tube [102]. Also refers to Staunig RoFo 1923;28:363. **ε** (B)= **ε** (A) {=0.69}
5. Glocker reports that at diagnostic energies the output of a transformer (Hochspannungsgleichrichter) on ion tube is about 2.5 to 1.4 times the output on a Coolidge tube (p441) [102]. At high voltages (168 kV) outputs are equal.
   For pelvis use **ε** = 1.4* **ε**(C1) {= 0.78}
6. Glocker shows for Snook with ion tube a deformed sine-shaped current [102], Fig. 6, Platte), voltage unknown. Assuming sine-V & sine-I: **ε** = 0.72 * 0.9 = 0.65.
7. Crowther finds that transformer on ion tube (Fig. 13) gives repeated sharp discharges (as Dauvillier shows in Fig 65) [104]. Suggests relatively high **ε** = 0.79*0.9=0.71, as of A. Crowther Fig. 14 shows similar characteristics as far as I(t) x V(t) concerns: **ε** = 0.71. (For "triangular-I" and "half-half triangular-V & DC-V" **ε** = 0.79 acc. to SpekCalc)
8. Kaye shows rather continuous tube currents, at least he says so about one tube, for another there could be separate discharges (Fig,18) [110]. But also current at lower voltages! Efficiency considerably less than for DC, but slightly better than for triangular voltage and current. In fact SpekCalc's "triangular-I" and "half-half triangular-V & DC-V". **ε** = 0.79*0.9=0.71
9. Wertheimer shows a rather high "start-discharge" and a current only during about a quarter a period (Fig.30)[111]. The current shape is continuous (it looks), say like a sine. But kV-peak

is only 18kV. Efficiency certainly better than V and I both true sines. About half V-sine&half V-triang: **ε** = 0.79 * 0.9 = 0.71.

10. Franke finds that a transformer on an ion tube and a Coolidge tube connected in-parallel and receiving exactly identical currents give completely identical photographs of the same hand [112] . **ε** (B)= **ε** (C2) {= 0.56}
11. Fritz: transformer & ion tube need half the mAs of transformer & Lilienfeldt tube [93,113]. From clinical photographs. Coolidge not Lilienfeldt. Not used.
12. Fritz: same Photographs of Snook-Transformer-Ion tube as of Inductor-ion tube [113]. **ε** (B)= **ε** (A) {= 0.69}
13. See at A (Fritz); ε (B)= ε (A) {= 0.69}
14. See at A (Wertheim-Salomonson); ε (B)= ε (A)/1.25 {= 0.56}
15. In clinical exposures Snook-ion tube two or three time more efficient than Snook on Coolidge according to Gorton [114]. Comparison of heavily attenuated X-rays, not of unattenuated air kerma. Not used.

C1. Snook (Transformer+mechanical rectifier) and Coolidge tube (current saturated)

1. Dauvillier [30]: For Snook system only short periods are current free (Fig. 12). Current when it flows about constant. There are spikes at make (interrupted arcing at rotating contacts) [30]. **ε** =0.55 if voltage sine (SpekCalc).
2. Glocker: from Abb. 3 current roughly constant for Snook (Abb 3a and 3b, 4 and 5 (look at relative change). No V shown! Probably **ε** =0.55 if Vis a sine (SpekCalc).
3. Crowther reports current saturation, so current rather flat (but not completely), voltage is about a sine (Fig. 7). Profiles are narrower than for transformer, but "useful" to "non-useful" ratio is rather similar to transformer with kenotron (if anything, still substantial current at low V). **ε** =0.55, not higher (SpekCalc).
4. Glocker reports that at diagnostic energies the output of a transformer (Hochspannungsgleichrichter) on ion tube is about 2.5 to 1.4 times the output on a Coolidge tube (p441) [102]. At high voltages (168 kV) outputs are equal. **ε** (C1) = **ε** (B)/(1.4 a 2.5). Use 1.4 for pelvis voltage, **ε** (C1) = **ε** (B)/1.4 {=0.51}
5. Glocker shows for Snook with Coolidge tube a deformed sine-shaped voltage (Fig.6 in text), current Fig. 3a (Platte) relatively flat (constant). **ε** calculated for digitized V&I with SpekCalc: **ε** = 0.72.
6. Wertheim Salomonson: 25% higher output for inductor-ion tube than for Snook-transformer-ion tube [108]. ε (C1) = 0.8 *ε (A) {= 0.56}.
7. Taylor [115]: for high 120 kV-peak about a factor of 0.6/1.32=0.45 of DC; at lower kV and thinner glass the ratio becomes even lower (according to simulations). **ε** = 0.45
8. Dalton [28] compares 4-pulse, 2-pulse and Snook: Snook and 2-pulse identical, 4-pulse gives 25% less output (according to Holzknecht Quantimeter). Average of 2- and 4 pulse: **ε** (C1) = 1.125***ε** (C2) {= 0.63}

9. According to Orliansky [116] a Snook system gave less and softer output than a transformer rectified in another way. Not used (but confirms Snook doesn't what expected; by using crests-only higher efficiency).
10. The same can be concluded from Fig 3 in Eddy [117] (from Rieber, [63]). Not used.
11. Chantraine [118]: "Die gasfreie Röhre wird bei Gleichspannung wenigstens die doppelte Strahlenmenge liefern, die sie beim Betrieb mit dem Scheibengleichrichter zu liefern vermag." **ε** =0.5.

C2. Transformer and Coolidge tube (current saturated)

1. Dauvillier reports that the output of unfiltered X-rays at 100 kV is 2.2 times less than for the reference DC-Coolidge combination (P135). Idem for 48 kV on P138 [29]. **ε** =0.45.
2. Dauvillier reports that a transformer on a Coolidge tube gives a factor of 1.7 less output than that transformer does on a gas tube, P133 [29]. **ε** =B/1.7 {=0.42}
3. Dauvillier shows the current is nearly constant [30]. Start current at X-ray generation-phase is relatively high [30]. SpekCalc under this condition **ε** =0.55.
4. Glocker: DC-Coolidge gives 1.5*inductor-Coolidge and 1.6*transformer-Coolidge **ε** =0.63.
5. Glocker reports that at high voltage the output of inductor and transformer is equal on Coolidge tube (he shows it at high voltage, 264 kV). Same finding from Coolidge and Kearsly (200 kV DC and 235 kV-peak sinus have same attenuation) [101]. Too high kV.
6. Glocker: from Abb. 3 current not nearly constant. For Snook better constant (Abb 3a and 3b, 4 and 5 (look at relative change). Might indicate higher efficiency than **ε** =0.55 (SpekCalc). Say **ε** =0.6
7. Crowther reports current saturation, so current flat (but not completely), voltage is about a sine. With Snook the profiles are narrower, but "useful" to "non-useful" ratio is rather similar to transformer with kenotron. **ε** =0.55 (SpekCalc).
8. Bouwers used a construction ("grid") to have most current during the voltage peak [119]. Not used.
9. Kaye shows saturation of current (Fig. 7 bottom and text P 485) [110]. Like Dauvillier **ε** = 1/2.2 = 0.45
10. Codd finds a 20% lower efficiency compared to inductor-gas tube set [107]. **ε** = **ε** (A)*0.8 {= 0.56}
11. Farrer finds at 1 mA that Transformer-Coolidge needs 60-70% more mA than Coil-Coolidge (p369) [120] (if and only if the coil can handle the load easily, otherwise low voltage and high current and no X-rays). **ε** = 1.65* **ε** (D). Not used, D bad combination.
12. Franke finds that a transformer on an ion tube and a Coolidge tube connected in-parallel and receiving exactly identical currents give completely identical photographs of the same hand [112]. **ε** (C2) = **ε** (B) {= 0.72}. (equal below X-rayed hands, thus also approximately above, little attenuation).

13. Fritz: transformer & ion tube need half the mAs of transformer & Lilienfeldt tube [93]. From clinical photographs. Lilienfeldt probably not equivalent with Coolidge. Not used.
14. Glocker: DC-Coolidge gives 1.5*inductor-Coolidge and 1.6*transformer-Coolidge. **ε** = 1/1.6 =0.625.
15. Hull [121] gives a factor of 2/3, but from his Fig. 10 this is an overestimation. I.e. real factor is smaller; say **ε** = 0.6.
16. Taylor [115], Fig. 4: for high 140 kV-peak about a factor of 1.17/2.32=0.50 of DC. NB: 1.17 average of 5 tubes; at lower kV and thinner glass the ratio becomes lower (see simulations). Half-wave similar (Fig.5). **ε** =0.50
17. Codd [107]. **ε** (C2) = **ε** (A)/1.2 {=0.58}

D. Inductor and Coolidge tube

This a bad combination according to Dauvillier [29]. Possibility to shortcircuit the coil by high emission in Coolidge, resulting in low voltage, nevertheless relatively high current but few X-rays.

1. Glocker reports lower output than for Transformer-Coolidge Tube (for filtered radiation a factor of 2.5 to 1.4), a difference that disappears at real high voltage (200 kV) [102]. But spectral differences are small.
2. Crowther reports large useless currents, as many others do [104].
3. Owen does the same [105,122]; Tr-Cool, Tr-Ion&Ind-Ion linear relation between mAs and Ionization [105], Fig. 1. Radiation softer than for coil-gastube & Transf-Coolidge [105].
4. Schall does the same [123].
5. Dauvillier also [30].
6. Levy shows this nicely in a graph (his Fig. 6) [124]. 40 mAs for C2 and 140 mAs for D, ergo ε = ε (C2)/(140/40) {= 0.16}
7. Levy also states that at low mA (1 mA) the efficiency of the coil-Coolidge combination is much more eff. than the interrupterless-transformer: 20 mA.min versus 50 mA.min, but these look extreme curve ends, rather take 20 and 35 mAs [124]. ε (D)= 1.75 * ε (C1) {= 0.98}.
8. Farrer also finds useless mA's. But at 1 mA he finds that Transformer-Coolidge needs 60-70% more mA than Coil-Coolidge [120]. **ε** = **ε** (C2) * 1.65 {=0.92}.
9. Glocker reports a sine-like voltage for a coil on a Coolidge tube [102], contrary to what most investigators report.
10. Glocker: DC-Coolidge gives 1.5*inductor-Coolidge and 1.6*transformer-Coolidge. **ε** = 1/1.5 =0.67.
11. Ledoux made comparisons with DC, but not in primary beam, but behind a skull (bone only) as object and using 45 kV. The transformer-Coolidge combi needs 5 times more radiation, the Snookes transformer with rotating rectifier 3 times more, the transformer-Lilienfeldt tube 3 times, the Mueller tube 4 times, and finally the coil (inductor)-Coolidge 4 times [31]. Note that attenuation accentuates spectral differences, and kV is low! Not used.
12. Codd [125] says a factor 2 less efficient than inductor on gas tube. ε (D) = ε (A)/2 {= 0.35}

13. Russ [126] shows the typical low efficiency and large currents.
14. Donnithorne [127] says the same.
15. Richards [128] shows sine voltage and current, and much (too) narrow X-ray output pulse measured with ionization chamber (CH_3I).

ES XII. Estimation of uncertainty in the results of computational method

Part of dose reconstruction should be the assessment of its accuracy. The latter is difficult in our case because the old literature doesn't provide uncertainties in the various parameters we used in the computations. We estimated therefore uncertainties ourselves, and used them in an elementary "error analysis". Because the KfiA is the primary output from our calculations, we assess the uncertainty in that parameter. In case the available information only pertains to the secondary circuit, then according to first principles the following holds for the KfiA (to keep it practical we give the units actually used in the computations):

$$KfiA = f(kV, d) * \varepsilon * I_{av} * \mathrm{t} * \frac{Z_{anode}}{Z_W} * \mathrm{FSD}^{-2}\ \ \mu Gy \qquad (Eq. 1),$$

with the function f(kV,d) giving the KfiA in µGy/mAs at 1 m, assuming the X-ray tube has a glass wall of d mm thickness and is driven by a DC high voltage kV. Further, ε is the X-ray efficiency of the system compared to DC, I_{av} the average tube current in mA, t the exposure time in s, Z the atomic number and FSD the focus-skin distance in meters. We limit ourselves to changes in KfiA around 75 kV and 0.85 mm tube wall, including 250 mm air between tube and patient. For our error analysis f(kV,d) is needed in two forms, one showing the dependence on changes in kV for fixed d (0.85 mm), and one showing the effect of changes in wall thickness for fixed high voltage (75 kV). The function f(kV,0.85mm) is shown in Fig. ES13 (left, linear dependence, DC): f(kV,0.85mm) = (3.2647 kV - 31.363) µGy/mAs at 1 m. Similarly f(75kV,d) = (-209.68 d + 396.33) µGy/mAs at 1 m (graph not shown).

In the following we use the letter σ for the standard deviation ("uncertainty"); the subscript identifies the pertinent parameter. For an uncertainty of σ_{kV} in the kV, the relative kV-induced uncertainty in air kerma around 75 kV can be approximated by $1.15\frac{\sigma_{kV}}{kV}$, as can easily be shown using the linear expression for f(kV,0.85mm). Similarly, one finds from (f(75kV,d) that around a wall thickness of 0.85 mm the relative wall thickness induced uncertainty in air kerma is approximately given by $0.82\frac{\sigma_d}{d}$. Since the air kerma is linearly dependent on the other parameters in the above equation, except the FSD of course, one obtains for the total relative uncertainty in the calculated air kerma:

$$\left(\frac{\sigma_{KfiA}}{KfiA}\right)^2 = \left(\frac{1.15\sigma_{kV}}{kV}\right)^2 + \left(\frac{\sigma_\varepsilon}{\varepsilon}\right)^2 + \left(\frac{\sigma_{I_{av}}}{I_{av}}\right)^2 + \left(\frac{\sigma_t}{t}\right)^2 + \left(\frac{0.82\sigma_d}{d}\right)^2 + \left(\frac{2\sigma_{FSD}}{FSD}\right)^2.$$

When I_{av} has to be obtained from data applying to the primary and secondary circuit (assuming sine voltage and sine current) Eq. 1 reads:

$$KfiA = f(kV, d) * \varepsilon * 1.2732\ \frac{\alpha\, V_{prim}\, I_{prim}}{kV} * \mathrm{t} * \frac{Z_{anode}}{Z_W} * \mathrm{FSD}^{-2}$$

When the kV in the middle of this equation is put below f(kV,d) one gets for fixed d the following relation $\frac{f(kV,0.85mm)}{kV} = 3.2647 - \frac{31.363}{kV}$, which is nearly constant (varies 2% for 10 kV change). But uncertainties in α (electrical transformation efficiency of generator system), V_{prim} (DC primary voltage) and I_{prim} (average primary current) come into play, and in a linear way. Then the following approximately holds for the total relative uncertainty:

$$\left(\frac{\sigma_{KfiA}}{KfiA}\right)^2 = \left(\frac{\sigma_\varepsilon}{\varepsilon}\right)^2 + \left(\frac{\sigma_\alpha}{\alpha}\right)^2 + \left(\frac{\sigma_{I_{prim}}}{I_{prim}}\right)^2 + \left(\frac{\sigma_{V_{prim}}}{V_{prim}}\right)^2 + \left(\frac{\sigma_t}{t}\right)^2 + \left(\frac{0.82\sigma_d}{d}\right)^2 + \left(\frac{2\sigma_{FSD}}{FSD}\right)^2.$$

Finally, the dependence of the relative uncertainty in KfiA on the various uncertainties in case we base our calculations on an estimate of the anode temperature is given by:

$$\left(\frac{\sigma_{KfiA}}{KfiA}\right)^2 = \left(\frac{\sigma_{P(T)}}{P(T)}\right)^2 + \left(\frac{\sigma_\varepsilon}{\varepsilon}\right)^2 + \left(\frac{\sigma_t}{t}\right)^2 + \left(\frac{0.72\sigma_d}{d}\right)^2 + \left(\frac{2\sigma_{FSD}}{FSD}\right)^2.$$

The allowed thermal load for an anode temperature T, P(T), can be obtained using Fig. ES5. Note we considered for the early small tubes also 75 kV, but a thinner tube wall of 0.6 mm.

In practice it is very difficult to make reasonable guesses for the various sigmas, but we tried (often somewhat conservatively we think) and got at least some idea of the final uncertainty in our dose reconstructions. Note that considering "maximum deviations" in parameters is impractical and makes little sense. In principle some additional uncertainty will be introduced by SpekCalc. We will see that the default value of its calibration factor N_f of 0.68 appears to apply pretty well in our work (as it should theoretically).

ES XIII. Results from chromoradiometers

Levy-Dorn [129] reported that the dose needed for a pelvic radiograph amounted to 1/20 of 1.5 H(olzknecht), with the dose specified in the detector plane at 60 cm from the X-ray focus. At the position of the skin, at 30 cm from the focus the dose is four times higher (it is Levy-Dorn who specifies a 30 cm focus-skin distance). One so finds for the kerma free in air KfiA = 4*(1/20)*1.5*(pastille dose/5) = 4*(1/20)*1.5*(3906/5) = 234 mGy. With BSF=1.17 one finds ESAK = 274 mGy.

Kienböck [4] specified the dose for an X-ray of the kidney using the "Kompressionsblende" as 1/6 X, also in the plane of the photographic plate. We assume the compression diaphragm caused a distance of 39 cm between the focus and the skin [53], and assuming a 20 cm thick patient, 1 cm between patient and plate, one finds for the kerma free in air at the entrance location 1/6*(pastille dose/10)*$(60/39)^2$ = 1/6*3906*$(60/39)^2$ = 154 mGy, and for ESAK 180 mGy. We have a check on Kienböck's dosimetry as he also allows the calculation of the pastille dose. He advised that 1 X was incurred by an exposure characterized by: a hardness BW 5 or an 18 cm spark gap, 0.1 mA, 60 seconds exposure, 5 cm focus-quantimeter (p290). In 1905 Kienböck must have used a Pt anode under 45 degrees and the X-ray efficiency ε

of the inductor and ion tube will have been about 0.69 (Table 3). As 1X is 1/10 of a pastille dose, we now can calculate it. Assuming a glass wall thickness of 0.85 mm (equivalent with 0.76 mm Al), and using 89 kV and 110 kV (from 5 BW and 18 cm spark gap, respectively) one finds with the help of SpekCalc estimates for the pastille dose of 4.6 Gy and 5.8 Gy. These values are larger than the previously given 3.9 Gy, but not impossible considering the limitations of the method.

Dalton et al. [28] also used Holzknecht's device, but they specified their results in an indirect way: at 73 kV-peak, 5 mA, 300 s exposure time and the "Holzknecht sheet" at 15 cm from the focus a reading was obtained of 7.5 – 10 H. These data give a relation between electrical data and the pastille dose. He further reports that his pelvis photographs are made at 80 kV, 30 mA, 2.5 s exposure time and a focus-film distance of 27 inch. From these combined data (and 5H = 3.9 Gy and taking trivial differences in mAs, kVp and distance into account) one finds a kerma free in air of 46 mGy, i.e ESAK = 54 mGy (effect of kV change implemented via SpekCalc's air kerma). Using the electrical data directly in SpekCalc yields ESAK = 59 mGy, in good agreement. Dalton et al. used the Bucky-Potter grid and intensifying screens.

ES XIV. Results, reconstructed and retrieved

- *Time dependence of ESAK in Fig. 4*

The overall time dependence of the dose in Fig. 4 looks rather smooth, but a number of low doses stand out, clearly coming from "the avant garde" in dose reduction. Braun [130] noticed in 1928 that he, like Saupe [131], could do with no more than about 1 R per radiograph, but in a table with practical values he reported 7 R. The low doses in 1938, 1939, 1953 and 1957 were reported by Ardran and colleagues [132-134]. Ardran worked on dose reduction in research institutes. Billings [135] states with respect to his 1957 ESAK of 4.4 mGy: "The resulting skin dose is five to ten times less than that reported by other investigators". Persliden et al. in 2002 [136] reported even lower doses than 0.7 mGy, but this was the lowest dose reportedly giving satisfactory images.

The 2018 result from the Maastricht University Medical Center, is 0.82 mGy (n=348, adult patients; personal communication Cécile Jeukens). The computational method gave an 8% lower value, 0.76 mGy. The result is only slightly higher than the 2012 value of 0.71 mGy for larger 10-15 year old children [137] (obtained after optimization of imaging protocols).

The data in Fig. 4 could well be fitted with a single exponential. Adding a second degree term in the exponent, to allow for curvature in the logarithmic graph, did improve the fit only slightly when the low data points mentioned above had been removed: R^2 increased from 0.8716 to 0.8798. Fig. ES15 shows the collected ESAK values sorted according to their origin.

On a linear scale the distribution of doses (normalized with fitted exponential function) is heavily skewed (Fig. ES16, left), but on a logarithmic scale the distribution is nearly symmetric (Fig. ES16, right). The equivalent of Fig. 4 with a linear vertical scale is shown in Fig. ES17. This graph illustrates the tremendous spread at all times (Fig. ES17), illustrating even better than Fig. 4 missed opportunities for lowering dose by optimization. We will not discuss how the

average dose was lowered over time, as most of it is well known. For the interested reader we recommend the review by Eisenberg [138].

An important cause of variability in exposures in the ion tube era was the unintended change in gas pressure in the tube. Another was unwarranted use, which could result in extremely high doses. Mitchell et al. [139] and Cassidy [140] reported about such X-ray incidents in 1898 and 1899. Judging from the reported wet desquamation and necrotization following exposure, the dose during the imaging of respectively a renal calculus and a hip must have been above 18 Gy according to ICRP Report 118 [141]. Deutschländer [142] made 5 hip radiographs in the same patient in two days in 1899. Wet desquamation followed. As he gave all specifications (in 1899!) of the exposure the total ESAK could be calculated as 19 Gy. His best radiograph obtained with an exposure time of 10 minutes corresponded to a skin dose of 3.6 Gy. Probably many more of such accidents happened.

- *A check of our computational approach*

A number of authors reported both doses and exposure parameters. We considered these “double” data from the earliest period possible, i.e. from 1927 onwards, to about 1957, as they might be best representative for the period before 1927 in terms of radiation softness. In 32 instances we were able to compare measured and computed doses, all from transformer-Coolidge systems (including Snook transformers), resulting in an average ratio of 0.97±0.21 (Fig. 6). This is satisfying and it suggests that the chosen thickness of the glass wall of the X-ray tube was fortunate (an arbitrary choice within the possible range of thicknesses for reasonably sized tubes). It also testifies to the plausibility of other parameters that had to be derived for our computations, but compensation of errors cannot be excluded. Note that only the measured doses were included in Fig. 4, not those computed for this comparison.

There is no reason to assume that the pre- and post-1927 doses computed for Coolidge tubes differ substantially in reconstruction errors (i.e. ≤ 21%). Now notice that in the period 1913-1922 doses from X-ray systems consisting of an ion tube with an inductor/Snook-transformer (red squares in Fig. ES15, top left) roughly match in amplitude and spread with the reconstructions for the Coolidge tube systems (green squares): average 317±163 mGy (n=34) vs 293±147 mGy (n=14). The reconstruction error in ion tube systems might therefore be not too different from that for Coolidge systems, at least when a similar spread in doses introduced by the users and their systems did exist. These results seem to suggest that the reconstruction uncertainty for all X-ray systems from this period does not exceed the 40% we estimated before.

One is often warned that dose estimation using electrical parameters may be quite inaccurate. It is true that large deviations between calculations and measurements have been reported, but our impression is that the correspondence is mostly rather acceptable (as suggested by Fig. 6). The user manual of PCXMC [143] states: “The accuracy of specifying the input dose by the tube current-time product (mA.s) is about 30%” (maximum deviation?). Moreover, many identical systems were reported to give approximately identical (measured) doses. Two examples only: Sorrentino and Yalow in 1950, who report that the output for seven systems is within 10%

[144], and McCullough and Cameron [145], who imply in the comparison in their Table 1 similar output in six different studies. Such results imply a consistency between systems working under the same conditions that is at the basis of dose simulations. An unknown variable in the past was often the inherent filtration. Some tubes for skin therapy had very thin windows [12] and not accounting for this in radiography would of course result in unexpectedly high doses.

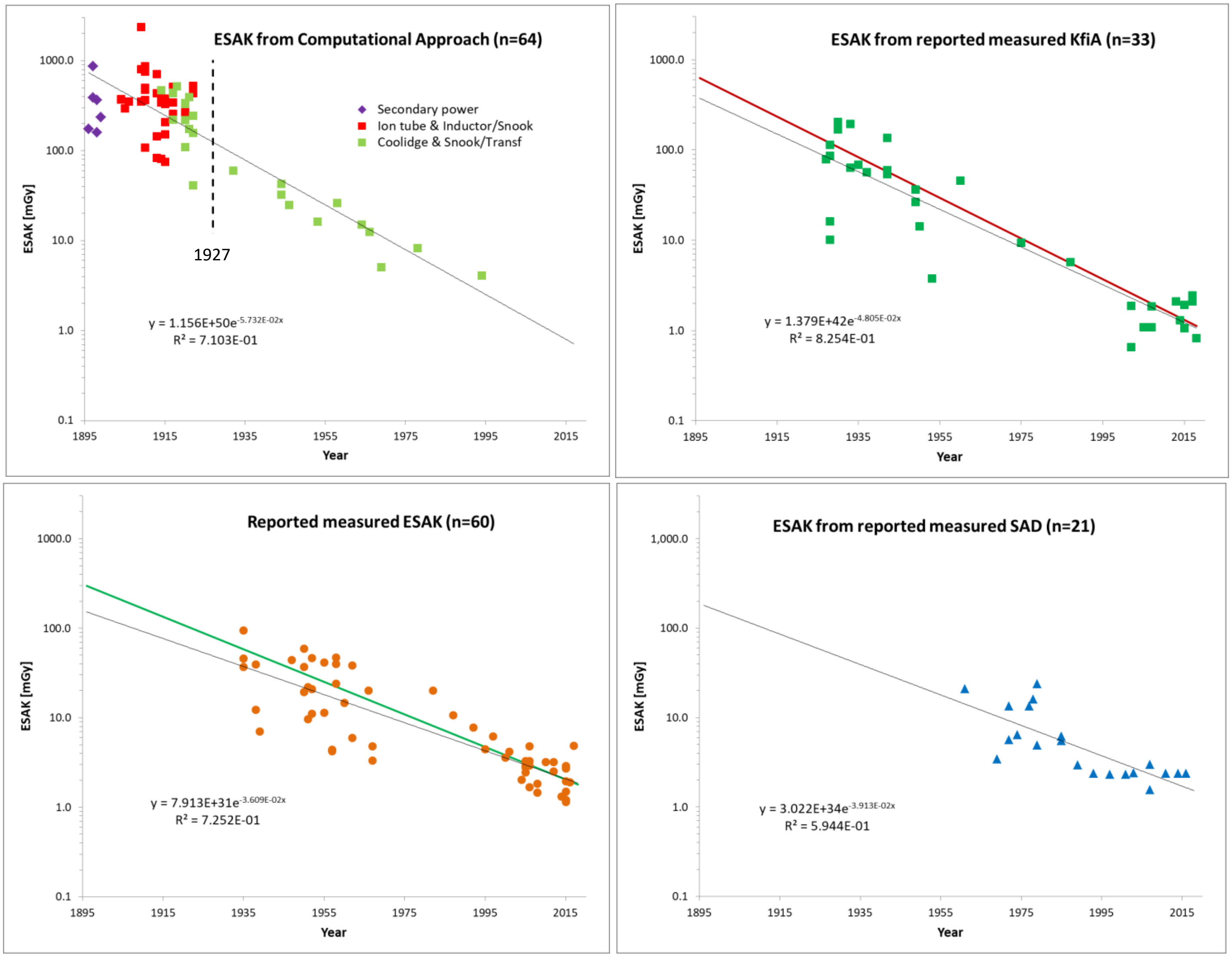

Fig. ES15 ESAK pelvic radiograph since 1896. Data sorted according to their origin. The fits with a simple exponential function (black lines) show to some extent the impact of the separate data groups on the summary fit in Fig. 4. The effect of not considering four “avant garde doses” (i.e. exceptionally low values at the time of reporting) in graph “ESAK from reported measured KfiA” is shown by the red line. Similarly, the effect of not considering seven low values in “Reported measured ESAK” is illustrated by the green line

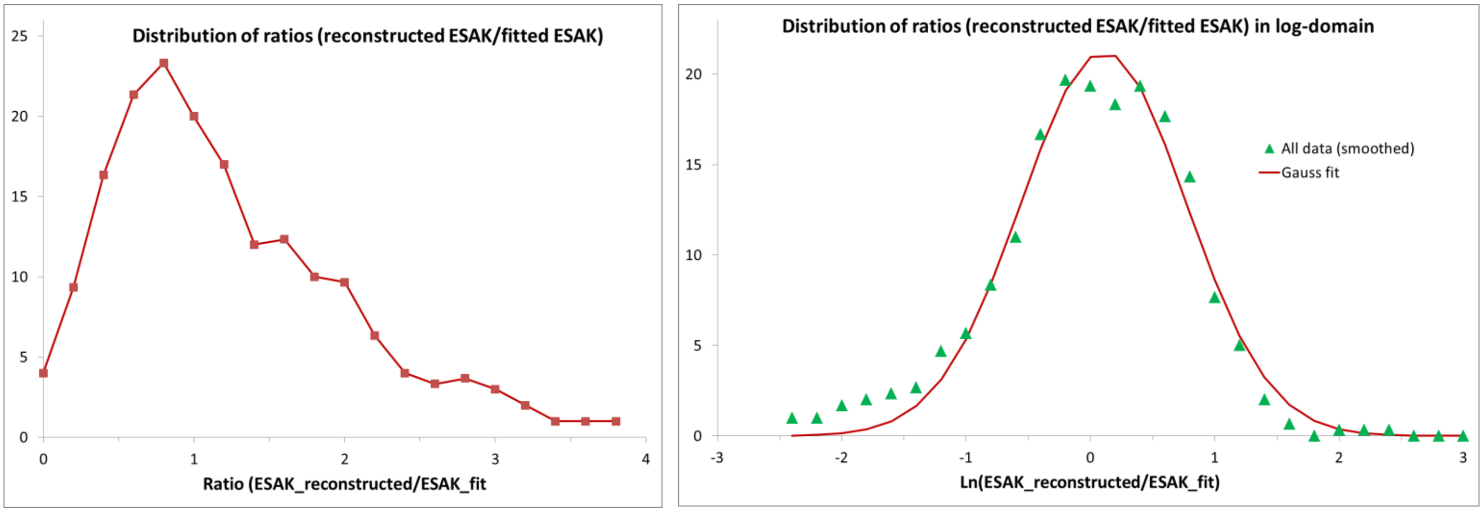

Fig. ES16 Left: relative spread in ESAK from 1896-2018 in linear domain (x-axis is linear; n=182). Right: the same on a logarithmic x-axis, including a Gaussian fitted to the data. Note the distribution is heavily skewed in the linear domain, but rather symmetric in the log-domain. Points summed within intervals (x-axis) of 0.2; data are smoothed.

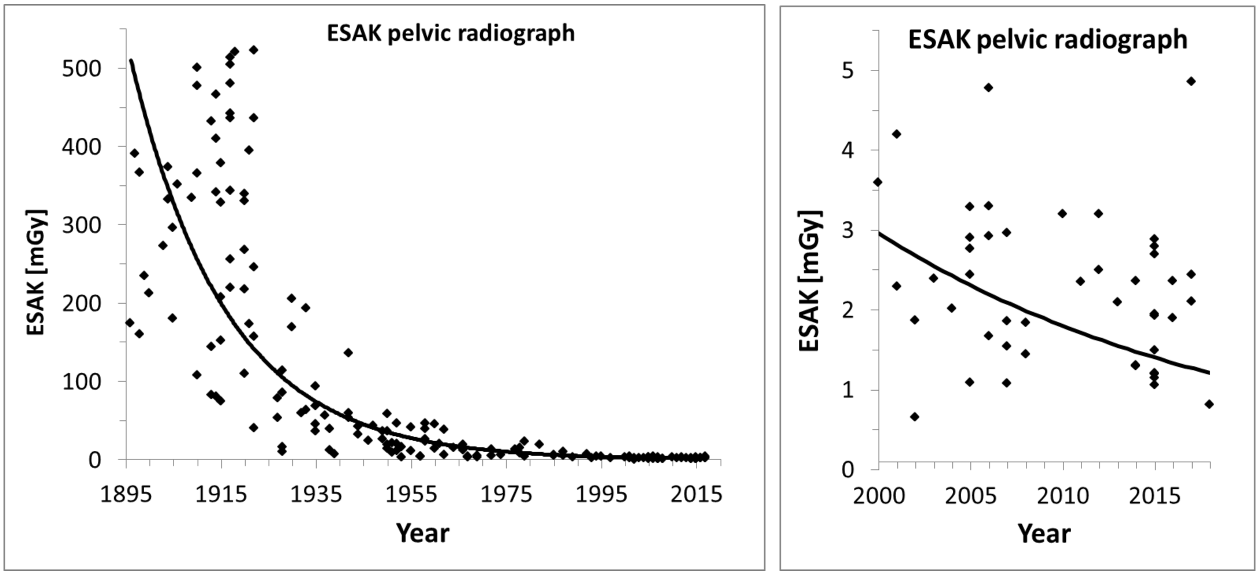

Fig. ES17 Spread and reduction in ESAK over the years on a linear vertical scale. The curve represents the exponential function fitted to the data. To the right the more recent data on an appropriate scale

References

1. Kaye GWC, Binks W (1929) The Evaluation of the X-Ray Pastille Dose in Röntgen (r) Units. Br J Radiol 2:553–578. https://doi.org/10.1259/0007-1285-2-24-553

2. Gleßmer-Junike S (2015) X-Strahlen, Radiometer und Hauteinheitsdosis. Thesis Universität Hamburg

3. Holzknecht G (1902) Eine neue einfache Dosierungsmethode in der Röntgentherapie (Das Chromoradiometer). Fortschritte auf dem Geb Röntgenstrahlen 6:108

4. Kienböck R (1905) Über Dosimeter und das quantimetrische Verfahren. Fortschritte auf dem Geb Röntgenstrahlen 9:276–295

5. Sabouraud RJA, Noiré H (1904) Traitement des teignes tondantes par les rayons X. Presse Médicale 12:825–827

6. Grebe L, Martius H (1924) Vergleichende Messungen über die Größe der zur Erreichung des Hauterythems gebräuchlichen Röntgenstrahlenmengen. Strahlentherapie 18:395–409

7. Reisner A, Neeff TC (1929) Hauttoleranzdosis und Strahlenqualität. Strahlentherapie 33:313–339

8. Schall L (1926) Zur Hauterythemfrage. Strahlentherapie 23:354–361

9. Reisner A (1933) Hauterythem und Röntgenstrahlung. Ergeb Med Strahlenforsch Röntgendiagnostik Röntgen- Radium- Lichttherapie 6:1–60

10. Schall WE (1932) X Rays: their origin, dosage and practical application, Fourth Edition. John Wright & Sons LTD, Stone Bridge, Bristol

11. Reisner A (1929) Wie sollen Röntgenbestrahlungen in der Oberflächentherapie dosiert werden? Röntgenprax Diagn Röntgen- Radium- Lichttherapie 1:825–831

12. Neeff, Th.C. (1930) Toleranzdosen in der Röntgendiagnostik. Fortschritte auf dem Geb Röntgenstrahlen 41:414–426

13. Poludniowski G, Landry G, DeBlois F, et al (2009) SpekCalc: a program to calculate photon spectra from tungsten anode x-ray tubes. Phys Med Biol 54:N433-438. https://doi.org/10.1088/0031-9155/54/19/N01

14. Poludniowski GG, Evans PM (2007) Calculation of x-ray spectra emerging from an x-ray tube. Part I. Electron penetration characteristics in x-ray targets: Calculation of x-ray spectra. Part I. Med Phys 34:2164–2174. https://doi.org/10.1118/1.2734725

15. Poludniowski GG (2007) Calculation of x-ray spectra emerging from an x-ray tube. Part II. X-ray production and filtration in x-ray targets: Calculation of x-ray spectra. Part II. Med Phys 34:2175–2186. https://doi.org/10.1118/1.2734726

16. Seltzer S (1995) Tables of X-Ray Mass Attenuation Coefficients and Mass Energy-Absorption Coefficients, NIST Standard Reference Database 126. https://www.nist.gov/pml/x-ray-mass-attenuation-coefficients Accessed: 9 Sept 2018

17. XCOM. NIST XCOM: Element/Compound/Mixture. https://physics.nist.gov/PhysRefData/Xcom/html/xcom1.html. Accessed 20 Aug 2018

18. Graph Grabber 2.0. https://www.quintessa.org/software/downloads-and-demos/graph-grabber-2.0. Accessed: 1 Sept 2018

19. Microsoft Excel (2010) Excel, Microsoft Office Professional Plus 2010. Microsoft

20. Hirsch IS (1920) The Principles and Practice of Roentgenological Technique. American X-Ray Publishing Co., New York

21. Kütterer G (2005) Ach, wenn es doch ein Mittel gäbe, den Menschen durchsichtig zu machen wie eine Qualle! die Röntgentechnik in ihren ersten beiden Jahrzehnten - ein besonders faszinierendes Stück Medizin- und Technikgeschichte, dargestellt in Zitaten. Books on Demand, Norderstedt

22. Kemerink M, Dierichs TJ, Dierichs J, et al (2012) The Application of X-Rays in Radiology: From Difficult and Dangerous to Simple and Safe. Am J Roentgenol 198:754–759. https://doi.org/10.2214/AJR.11.7844

23. Snook HC (1908) A new Roentgen generator. Arch Roentgen Ray 13:186–188

24. Phillips CES (1925) Constant Voltage High Tension Generators. Br J Radiol Röntgen Soc Sect 21:91–103. https://doi.org/10.1259/rss.1925.0033

25. W.F.H. (1918) New Technique: The Practical Applications of Thermionic Emission. Arch Radiol Electrother 22:355–356. https://doi.org/10.1259/are.1918.0083

26. Behling R (2015) Modern diagnostic X-ray sources: technology, manufacturing, reliability. CRC Press/Taylor & Francis, Boca Raton

27. Janus F (1927) Die Messung der Röhrenspannung. Verhandlungen Dtsch Röntgen Ges 18:86

28. Dalton CHC (1927) The Comparative Efficiency of Different Types of Transformers. Br J Radiol BIR Sect 32:443–446. https://doi.org/10.1259/bir.1927.0136

29. Dauvillier, A. (1924) La Technique des Rayons X. Librairie Scientifique Albert Blanchard, Paris

30. Dauvillier A (1917) Sur la forme de la décharge électrique dans les tubes à rayons X. Rev Générale de l’électricité Paris 1:443–453

31. Ledoux-Lebard R, Dauvillier A (1921) Sur l’utilisation de tensions constantes en radiodiagnostic. Comptes Rendus Hebd Séances Académie Sci 173:382–384

32. Wright L (1897) The induction coil in practical work - including X-rays. Macmillan and Co. Limited, London

33. Gadeceau MA (2015) http://www.biusante.parisdescartes.fr/ressources/pdf/inventaires/tubesRX_Beclere_Gadeceau_2015.pdf

34. Rosenfeld, G. (1897) Die Diagnostik innerer Krankheiten mittels Röntgenstrahlen. Verlag von J.F. Bergman, Wiesbaden

35. Foveau de Courmelles F-V (1897) Traité de radiographie médicale et scientifique. Octave de Doin, Paris

36. Gocht, H. (1898) Lehrbuch der Röntgen-Untersuchung: zum Gebrauche für Mediciner. Verlag von Ferdinand Enke, Stuttgart

37. Howgrave-Howard RP (1900) X-Rays simply explained, second. Percival Marshall & CO., London

38. Massiot G, Biquard R (1917) Manuel pratique du manipulateur radiologiste. A. Maloine et fils, Paris

39. Ruhmer E (1904) Konstruktion, Bau und Betrieb von Funkeninduktoren und deren Anwendung. Hachmeister & Thal, Leipzig

40. Arthur, D., Muir, J. (1909) A Manual of Practical X-Ray Work. Rebman Company, New York

41. Béclère, Henri (1910) Le Radiodiagnostic des affections du foie. G. Steinheil, Editeur, Paris

42. Morton, E.R. (1915) A Text-Book of Radiology. E.B. Treat & CO, New York

43. Pallardy G, Pallardy M-J, Wackenheim A (1989) Histoire illustrée de la radiologie. R. Dacosta, Paris

44. Chapman WAJ (1972) Workshop Technology, Part 1, 5th ed. Elsevier Butterworth-Heinemann, Burlington, MA

45. Willey VJ (1908) Some Experiments with Roentgen Tubes with Respect to the Use of Tubes for Diagnostic Work. Arch Roentgen Ray 12:248–260. https://doi.org/10.1259/arr.1908.0115

46. Reimer L (1998) Scanning electron microscopy: physics of image formation and microanalysis, Second Edition. Springer, Berlin

47. Albers-Schönberg H, Walter B (1910) Die Röntgentechnik, Third Edition. Lucas Gräfe & Sillem, Hamburg

48. Macintyre, J. (1896) Roentgen Rays: photography of renal calculus; description of an adjustable modification in the focus tube. The Lancet 148:118

49. Roeser HT, Wensel WF (1969) Total emissivity (National Bureau of Standards). In: Weast RC (ed) Handbook of Chemistry and Physics, 50 th., p E236, The Chemical Rubber Company, Cleveland, Ohio

50. Rønne P, Nielsen ABW (1986) Development of the ion X-ray tube: with a description of the collection in the Medical Historical Museum, University of Copenhagen. C.A. Reitzel Publishers, Copenhagen

51. Isenthal AW, Snowden Ward H (1898) Practical Radiography - A handbook of the applications of the X-rays, second. Dawbarn & Ward, London

52. Walsh D (1899) The Röntgen rays in medical work, second. Baillière, Tindall and Cox, London

53. Albers-Schönberg, (1906) Die Röntgentechnik: Lehrbuch für Ärzte und Studierende. Second Edition. Lucas Gräfe & Sillem, Hamburg

54. (1920) Coolidge X-ray tube, Instruction Book 89136B. General Electric Company, Schenectady, N.Y.

55. Weber J (1791) Vollständige Lehre von den Gesetzen der Elektricität und von der Anwendung derselben. Anton Weber, Landshut

56. Luboshez BE (1925) On Measuring and Expressing X-Ray Quality in Radiography. Br J Radiol BIR Sect 30:81–89. https://doi.org/10.1259/bir.1925.0019

57. Kaye GWC (1914) X Rays, An introduction to the study of Röntgen rays. Longmans, Green & Co, London

58. Spiegler G, Zakovsky J (1928) Der Halbwellenapparat der Röntgentechnik. Fortschritte auf dem Geb Röntgenstrahlen 37:718–728

59. AIEE (1969) E 61: Spark gap voltages, in CRC handbook of chemistry and physics. Chemical Rubber Company, Cleveland, Ohio

60. Ullmann HJ (1921) The practical application of the sphere gap to roentgenotherapy. Am J Roentgenol 8:195–199

61. Surgeon General of the Army (1920) United States army X-ray manual. Paul B. Hoeber, New York

62. Committee on preparedness & ARRS (1917) X-ray Manual U.S. Army. L. Middleditch CO, New York

63. Rieber F (1922) Standardization of the measurement of tube potential. Am J Roentgenol 9:371–381

64. Peek FW (1915) Dielectric phenomena in high voltage engineering, First Edition. McGraw-Hill Book Company, Inc., New York

65. Benoist ML (1902) Définition expérimentale des diverses sortes de rayons X, par le radiochromomètre. Comptes Rendus Hebd Séances Académie Sci 134:225–227

66. Hackenbruch PT, Berger W (1915) Mittlerer Expositions-Tabelle in Milliampèresekunden. In: Vademekum für die Verwendung der Röntgenstrahlen und des Distraktionsklammerverfahren im Kriege. Otto Nemnich Verlag, Leipzig, p 111

67. Muir J, Reid A, Harlow FJ (1927) A manual of practical X-ray work. Paul B. Hoeber, Inc., New York

68. Christen T (1914) Zur Theorie und Technik der Härtemessung. Fortschritte auf dem Geb Röntgenstrahlen 22:247–253

69. Blumenthal F, Böhmer L (1932) Strahlenbehandlung bei Hautkrankheiten. Verlag von S. Karger, Berlin

70. Guilleminot MH (1914) Sur la loi d'action biologique des rayons X filtrés et non filtrés. Arch d'électricité Médicale 22:331–333

71. Reiniger (1922) "Ergos" der Röntgen-Apparat für den praktischen Arzt und den Facharzt. Reiniger, Gebbert & Schall, A.-G, Erlangen

72. Wertheim Salomonson (1905) Leistungsmessung an Induktorien. Verhandlungen Dtsch Röntgen Ges 1:123–125

73. Armagnat H (1908) The theory, design and construction of induction coils. McGraw Publishing Company, New York

74. Wertheim Salomonson (1905) The Induction Coil—Measurement of Energy and Current in the Secondary Circuit. J Röntgen Soc 2:27–33. https://doi.org/10.1259/jrs.1905.0023

75. Wertheim Salomonson JKA (1905) The Measurement of the Efficiency of the Induction Coil. Arch Roentgen Ray 10:129–131. https://doi.org/10.1259/arr.1905.0078

76. Wertheim Salomonson (1920) Discussion on the Efficiency of the High-tension Transformer as compared with the Induction Coil. J Royal Soc Med 13:105–131

77. Jaugeas, F. (1910) Radiographie rapide et radiographie instantanée. J Radiol Belge December:601–629

78. Jaugeas F (1911) Rapid and Instantaneous Radiology. Arch Roentgen Ray 15:372–387. https://doi.org/10.1259/arr.1911.0130

79. Allen EG (1899) The Efficiency of Interrupters for Induction Coils. Thesis University of Wisconsin

80. Brunel G (1900) Manuel de radioscopie par l'emploi des rayons X. Librairie Bernard Tignol, Paris

81. Reiniger/Gebbert/Schall (1912) Die Röntgenapparate nebst deren Zubehör. Reiniger, Gebbert & Schall, A.-G., Berlin - Erlangen

82. Turner D (1902) A manual of practical medical electricity - The Röntgen rays and Finsen light. William Wood & Company, New York

83. Biddle (1904) Roentgen induction coils and other X-ray apparatus. James G. Biddle, Philadelphia

84. Janus F (1909) Der Expositionsmesser, ein neues Hilfsinstrument für Röntgenaufnahmen. Fortschritte auf dem Geb Röntgenstrahlen 14:253–256

85. Crookes W (1879) Radiant matter: a lecture delivered before the British Association for the Advancement of Science in Sheffield

86. Siemens & Halske (1904) Röntgenröhre (Patent 156746).

87. Siemens & Halske (1904) Röntgenröhre (Patent 165138)

88. Coolidge WD (1913) A Powerful Röntgen Ray Tube with a Pure Electron Discharge. Phys Rev 2:409–430. https://doi.org/10.1103/PhysRev.2.409

89. Goetze O (1923) Verfahren und Glühkathodenröntgenröhre zur Erzeugung scharfer Röntgenbilder (Patent 370022)

90. Hübscher M (1995) Thüringer Glas - Werkstoff der ersten Röntgenröhren. In: 100 Jahre Röntgenstrahlen - Thüringer Beiträge. Technische Universität Ilmenau, Ilmenau, pp 62–65

91. Walter B (1907) Über die Strahlungsregionen der Röntgenröhren und die Absorption ihrer Strahlung in ihrer Glaswand. Fortschritte auf dem Geb Röntgenstrahlen 11:340–349

92. Christen T (1913) Messung und Dosierung der Röntgenstrahlen. Fortschritte auf dem Geb Röntgenstrahlen Ergänzungsband 28:1–122

93. Fritz O (1922) Zum Ausbau der spektrometrischen Eichungsmethode. Fortschritte auf dem Geb Röntgenstrahlen 29:218–223

94. Schall & Son (1914) Exposure Table. In: Electro-Medical Instruments and their Management and Illustrated Price List of Electro-Medical Apparatus, 16e ed. John Wright & Sons Ltd, Stone Bridge, Bristol

95. Kotre CJ, Little BG (2006) Patient and staff radiation doses from early radiological examinations (1899−1902). Br J Radiol 79:837–842. https://doi.org/10.1259/bjr/16982267

96. Wucherpfennig V (1928) Die Dosierung mit der Sabouraud-Tablette unter dem Gesichtspunkt der Aluminium-Halbwertschicht. Strahlentherapie 27:353–357

97. Bucky G (1913) A Grating-Diaphragm to Cut off Secondary Rays from the Object. Arch Roentgen Ray 18:6–9

98. Holbeach CH (1921) The Bucky-Potter diaphragm. J Röntgen Soc 17:179–182

99. Wilsey RB (1922) The Efficiency of the Bucky Diaphragm Principle. J Röntgen Soc 18:77–89. https://doi.org/10.1259/jrs.1922.0020

100. Grigg ERN (1965) The trail of the invisible light. From X-Strahlen to Radio(bio)logy. Charles C. Thomas, Springfield, USA

101. Coolidge WD, Kearsly WK (1922) High voltage X-ray work. Am J Roentgenol 9:77–101

102. Glocker R, Kaupp E (1926) Oszillographische Untersuchungen an Röntgenröhren. Z für tech Phys 7:434–444

103. Owen EA (1926) Oscillographic Records of Induction Coil Discharges. Br J Radiol Röntgen Soc Sect 22:49–66. https://doi.org/10.1259/rss.1926.0029

104. Crowther JA (1924) Studies in X-Ray Production. Br J Radiol Röntgen Soc Sect 20:61–72. https://doi.org/10.1259/rss.1924.0022

105. Owen EA, Bowes PK (1923) Some Observations on Induction Coil and Transformer Discharges with Coolidge and Gas Tubes. J Röntgen Soc 19:78–83. https://doi.org/10.1259/jrs.1923.0027

106. Duddell J (1908) The Measurement of the Current through an “X” Ray Tube. J Röntgen Soc 4:100–114. https://doi.org/10.1259/jrs.1908.0057

107. Codd MA (1922) Investigations of Transformer Rectifier Sets. J Röntgen Soc 18:27–44. https://doi.org/10.1259/jrs.1922.0005

108. Wertheim Salomonson JKA (1910) Milliampèremeter and Röntgenlicht. Fortschritte auf dem Geb Röntgenstrahlen 16:291–296

109. Taylor Jones E (1932) Induction coil - Theory and applications. Sir Isaac Pitman & Sons, Ltd., London

110. Kaye GWC, Bell GE (1934) An Oscillographic Study of the Electrical Characteristics of X-Ray Generators. Br J Radiol 7:476–508. https://doi.org/10.1259/0007-1285-7-80-476

111. Wertheimer A, Wagner RH (1912) On Current and Tension Conditions (Characteristic) in Röntgen Tubes. J Röntgen Soc 8:49–55. https://doi.org/10.1259/jrs.1912.0027

112. Franke H (1926) Die Messung der bei der Aufnahme wirkenden Spannung und ihr Einfluβ auf das Röntgenbild. Verhandlungen Dtsch Röntgen-Ges 17:188

113. Fritz O (1922) Röhrenspannung und Bromsilberschwärzung. Fortschritte auf dem Geb Röntgenstrahlen 29:281–296

114. Gorton WS, Colston JAC (1916) The radiographic efficiency of the Coolidge X-ray tube. Am J Roentgenol 3:388–390

115. Taylor LS, Singer G, Stoneburner CF (1933) Comparison of high voltage X-ray tubes - RP595. Bur Stand J Res 11:341–351

116. Orliansky A (1930) The Output of a Transformer with Different Methods of Rectification of the High-Tension Current. Br J Radiol 3:316–319. https://doi.org/10.1259/0007-1285-3-31-316

117. Eddy CE (1932) The Output of Various Types of X-ray Machines and the Influence of Wave Form on Quality of X Radiation. Br J Radiol 5:892–902. https://doi.org/10.1259/0007-1285-5-60-892

118. Chantraine H (1926) Uber die Strahlenausbeute von gashaltigen und gasfreien Röhren. Fortschritte auf dem Geb Röntgenstrahlen 34:922–929

119. Bouwers A (1932) Economy and Quality in Radiology. Br J Radiol 5:311–323. https://doi.org/10.1259/0007-1285-5-52-311

120. Farrer WJG (1927) Exposures in Radiography. Br J Radiol BIR Sect 32:368–371. https://doi.org/10.1259/bir.1927.0112

121. Hull AW (1915) Roentgen ray spectra. Am J Roentgenol 2:893–899

122. Owen EA, Bowes PK (1921) X-Ray Dosage with Special Reference to the Barium Platino-Cyanide Pastille. J Röntgen Soc 17:107–119. https://doi.org/10.1259/jrs.1921.0053

123. Schall WE (1916) Experiments with a Coolidge Tube. J Röntgen Soc 12:74–76. https://doi.org/10.1259/jrs.1916.0044

124. Levy LA, Stenning HJ (1917) Some Remarks upon Pastilles. J Röntgen Soc 13:13–21. https://doi.org/10.1259/jrs.1917.0004

125. Codd MA (1921) Increasing the Efficiency of X-Ray Tubes by an Improved Design of Coil and Interrupter. J Röntgen Soc 17:67–78. https://doi.org/10.1259/jrs.1921.0031

126. Russ S (1915) Measurements of the Radiation from the Coolidge and other X-Ray Tubes in Clinical Use. J Röntgen Soc 11:42–47. https://doi.org/10.1259/jrs.1915.0026

127. Donnithorne HE (1916) A New Modification of the Ionization Method of Measuring X-Rays. J Röntgen Soc 12:77–83. https://doi.org/10.1259/jrs.1916.0046

128. Richards RC (1926) A Method of Studying the Behaviour of X-Ray Tubes. Proc R Soc Math Phys Eng Sci 112:280–288. https://doi.org/10.1098/rspa.1926.0111

129. Levy-Dorn, M. (1903) Schutzmassregelen gegen Röntgenstrahlen und ihre Dosirung. Dtsch Med Wochenschr 29:921–924

130. Braun R, Hase H, Küstner H (1928) Über die in der Diagnostik verabfolgten Dosen in R-Einheiten. Fortschritte auf dem Geb Röntgenstrahlen 38:385–398

131. Saupe, E. (1928) Über die bei diagnostischen Arbeiten verabreichten Röntgenstrahlendosen in R-Einheiten. Fortschritte auf dem Geb Röntgenstrahlen 37:536–540

132. Ardran GM, Crooks HE (1953) A Comparison of Radiographic Techniques with Special Reference to Dosage. Br J Radiol 26:352–357. https://doi.org/10.1259/0007-1285-26-307-352

133. Ardran GM (1956) The Dose to Operator and Patient in X-ray Diagnostic Procedures. Br J Radiol 29:266–269. https://doi.org/10.1259/0007-1285-29-341-266

134. Ardran GM, Crooks HE (1957) Gonad Radiation Dose from Diagnostic Procedures. Br J Radiol 30:295–297. https://doi.org/10.1259/0007-1285-30-354-295

135. Billings MS, Norman A, Greenfield MA (1957) Gonad Dose During Routine Roentgenography. Radiology 69:37–41. https://doi.org/10.1148/69.1.37

136. Persliden J, Beckman K-W, Geijer H, Andersson T (2002) Dose-image optimisation in digital radiology with a direct digital detector: an example applied to pelvic examinations. Eur Radiol 12:1584–1588. https://doi.org/10.1007/s00330-001-1206-0

137. Frantzen MJ, Robben S, Postma AA, et al (2012) Gonad shielding in paediatric pelvic radiography: disadvantages prevail over benefit. Insights Imaging 3:23–32. https://doi.org/10.1007/s13244-011-0130-3

138. Eisenberg RL (1992) Radiology: an illustrated history. Mosby Year Book, St. Louis

139. Mitchell, J.S., Haybittle, J.L. (1955) Carcinoma of the skin appearing 49 years after a single diagnostic roentgen exposure. Acta Radiol 44:345–350

140. Cassidy, P. (1900) Report of a severe X-ray injury. Med Rec 57:180–181

141. Stewart FA, Akleyev AV, Hauer-Jensen M, et al (2012) ICRP PUBLICATION 118: ICRP Statement on Tissue Reactions and Early and Late Effects of Radiation in Normal Tissues and Organs — Threshold Doses for Tissue Reactions in a Radiation Protection Context. Ann ICRP 41:1–322. https://doi.org/10.1016/j.icrp.2012.02.001

142. Deutschländer (1899) Beitrag zu dem Kapitel der Hautverbrennung durch Röntgenstrahlen. Fortschritte auf dem Geb Röntgenstrahlen 3:182–186

143. Tapiovaara M, Siiskonen T (2008) PCXMC: a Monte Carlo program for calculating patient doses in medical x-ray examinations, 2. ed. STUK, Helsinki

144. Sorrentino J, Yalow R (1950) A nomogram for dose determinations in diagnostic roentgenology. Radiology 55:748–753. https://doi.org/10.1148/55.5.748

145. McCullough EC, Cameron JR (1970) Exposure rates from diagnostic X-ray units. Br J Radiol 43:448–451. https://doi.org/10.1259/0007-1285-43-511-448

ES XV References for Fig. 4.

Note that several sources had more than one dose for a given year (there are data for 79 years).

Year	Reference	Year	Reference	Year	Reference
1896	[1]	1939	[51]	1987	[91], [92]
1897	[2], [3]	1942	[53]	1989	[82]
1898	[4], [5]	1944	[54]	1992	[93]
1899	[6]	1946	[55]	1993	[82]
1900	[7]	1947	[56]	1994	[94]
1903	[8]	1949	[57]	1995	[95]
1904	[9], [10]	1950	[58], [59]	1997	[82], [96]
1905	[11], [12]	1951	[60], [61]	2000	[95]
1906	[13]	1952	[62], [63]	2001	[82]
1909	[14], [15]	1953	[64], [57]	2002	[97]
1910	[16], [17], [18], [19]	1955	[59], [65]	2003	[82]
1913	[20],[21]	1957	[66], [67]	2004	[98]
1914	[22], [23], [24]	1958	[68], [69]	2005	[99], [100], [101]
1915	[25], [26], [27]	1960	[70], [71]	2006	[102], [103]
1917	[21], [28], [29], [30], [31]	1961	[72]	2007	[82], [104], [105]
1918	[31]	1962	[73], [74]	2008	[106]
1920	[31], [32]	1964	[75]	2010	[107]
1921	[33], [34]	1966	[76], [77]	2011	[82]
1922	[35], [36], [37], [38]	1967	[78]	2012	[108]
1927	[39], [40]	1969	[79]	2013	[109]
1928	[41], [42], [43]	1972	[80], [81]	2014	[110], [111], [82]
1930	[44]	1974	[82]	2015	[112], [113], [114], [115]
1932	[45]	1977	[83]	2016	[116], [117]
1933	[46]	1978	[84], [85]	2017	[118], [119]
1935	[47], [48], [49]	1979	[82], [86]	2018	[120]
1937	[50]	1982	[87], [88]		
1938	[51], [52]	1985	[89], [90]		

References

1. Macintyre, J. (1896) Roentgen Rays: photography of renal calculus; description of an adjustable modification in the focus tube. The Lancet 148:118

2. Rosenfeld, G. (1897) Die Diagnostik innerer Krankheiten mittels Röntgenstrahlen. Verlag von J.F. Bergman, Wiesbaden

3. Foveau de Courmelles F-V (1897) Traité de radiographie médicale et scientifique. Octave de Doin, Paris

4. Gocht, H. (1898) Lehrbuch der Röntgen-Untersuchung: zum Gebrauche für Mediciner. Verlag von Ferdinand Enke, Stuttgart

5. Isenthal AW, Snowden Ward H (1898) Practical Radiography - A handbook of the applications of the X-rays, second. Dawbarn & Ward, London

6. Walsh D (1899) The Röntgen rays in medical work, second. Baillière, Tindall and Cox, London

7. Kotre CJ, Little BG (2006) Patient and staff radiation doses from early radiological examinations (1899−1902). Br J Radiol 79:837–842. https://doi.org/10.1259/bjr/16982267

8. Levy-Dorn, M. (1903) Schutzmassregelen gegen Röntgenstrahlen und ihre Dosirung. Dtsch Med Wochenschr 29:921–924

9. Beck, C. (1904) Röntgen ray diagnosis and therapy. D. Appleton and company, New York

10. Holland CT (1904) Description of Plate: Plate I: The Left and Right Kidney Regions: Plate A. (the Left) shows the Shadow of One Stone. Plate B. (the Right) shows the Shadow of Three Stones. J Röntgen Soc 1:51–51. https://doi.org/10.1259/jrs.1904.0032

11. Biddle JG (1905) Typical "Roentgen" Equipments. James G. Biddle, Chestnut Street, Philadelphia.

12. Kienböck R (1905) Über Dosimeter und das quantimetrische Verfahren. Fortschritte auf dem Geb Röntgenstrahlen 9:276–295

13. Albers-Schönberg, (1906) Die Röntgentechnik: Lehrbuch für Ärzte und Studierende, Second Edition. Lucas Gräfe & Sillem, Hamburg

14. Janus F (1909) Der Expositionsmesser, ein neues Hilfsinstrument für Röntgenaufnahmen. Fortschritte auf dem Geb Röntgenstrahlen 14:253–256

15. Arthur, D., Muir, J. (1909) A Manual of Practical X-Ray Work. Rebman Company, New York

16. Béclère, Henri (1910) Le Radiodiagnostic des affections du foie. G. Steinheil, Editeur, Paris

17. Jaugeas, F. (1910) Radiographie rapide et radiographie instantanée. J Radiol Belge December:601–629

18. Jaugeas F (1911) Rapid and Instantaneous Radiology. Arch Roentgen Ray 15:372–387. https://doi.org/10.1259/arr.1911.0130

19. Tousey S (1910) Medical Electricity and Röntgen Rays. W.B. Saunders Company, Philadelphia

20. Siemens (1913) Ueber die Expositionszeit bei unseren Röntgeneinrichtungen. In: Vertrauliche Mitteilung über Elektromedizin. Siemens&Halske, Berlin, pp 6–8

21. Massiot G, Biquard R (1917) Manuel pratique du manipulateur radiologiste. A. Maloine et fils, Paris

22. Schall & Son (1914) Exposure Table. In: Electro-Medical Instruments and their Management and Illustrated Price List of Electro-Medical Apparatus, 16e ed. John Wright & Sons Ltd, Stone Bridge, Bristol

23. Cole, L.G. (1914) Vorläufige Mitteilung über die diagnostische und therapeutische Verwendung des Coolidge-Rohres. Fortschritte auf dem Geb Röntgenstrahlen 22:29–34

24. Reiniger, Gebbert & Schall (1914) Universal-Expositionstabelle. Reiniger, Gebbert & Schall, A.-G, Berlin-Erlangen

25. Knox, R. (1915) Radiography, X-RayTherapeutics and Radium Therapy, First Edition. The Macmillan Company, New York

26. Morton, E.R. (1915) A Text-Book of Radiology. E.B. Treat & CO, New York

27. Hackenbruch PT, Berger W (1915) Mittlerer Expositions-Tabelle in Milliampèresekunden. In: Vademekum für die Verwendung der Röntgenstrahlen und des Distraktionsklammerverfahren im Kriege. Otto Nemnich Verlag, Leipzig, p 111

28. Committee on preparedness & ARRS (1917) X-ray Manual U.S. Army. L. Middleditch CO, New York

29. Hammes, J. (1917) Über die Technik und den Wert seitlicher Wirbelaufnahmen. Fortschritte auf dem Geb Röntgenstrahlen 25, 1:1–13

30. Knox, R. (1917) Radiography and Radio-Therapeutics: Part I: - Radiography, Second Edition. A & C Black, Limited, London

31. Hirsch IS (1920) The Principles and Practice of Roentgenological Technique. American X-Ray Publishing Co., New York

32. Surgeon General of the Army (1920) United States army X-ray manual. Paul B. Hoeber, New York

33. Watson& Sons (Electro-Medical) Ltd (1921) Exposure chart for Coolidge X-ray tubes, circa 1921, 5-th ed. catalogue. In: Mould, A century of x-rays and radioactivity in medicine: with emphasis on photographic records of the early years. Institute of Physics Pub, Bristol ; Philadelphia, p 91

34. Holbeach CH (1921) The Bucky-Potter diaphragm. J Röntgen Soc 17:179–182

35. Reiniger (1922) “Ergos” der Röntgen-Apparat für den praktischen Arzt und den Facharzt. Reiniger, Gebbert & Schall, A.-G, Erlangen

36. Fritz O (1922) Zum Ausbau der spektrometrischen Eichungsmethode. Fortschritte auf dem Geb Röntgenstrahlen 29:218–223

37. Witherbee, W.D., Remer, J. (1922) X-Ray Dosage in Treatment and Radiography. The Macmillan Company, New York

38. Crowther, J.A. (1922) The Principles of Radiography. J & A Churchill, London

39. Dalton CHC (1927) The Comparative Efficiency of Different Types of Transformers. Br J Radiol BIR Sect 32:443–446. https://doi.org/10.1259/bir.1927.0136

40. Chantraine, H., Profitlich, P. (1927) Über die bei Durchleuchtungen und Aufnahmen verabfolgten Strahlenmengen. Fortschritte auf dem Geb Röntgenstrahlen 36:404–406

41. Saupe, E. (1928) Über die bei diagnostischen Arbeiten verabreichten Röntgenstrahlendosen in R-Einheiten. Fortschritte auf dem Geb Röntgenstrahlen 37:536–540

42. Braun R, Hase H, Küstner H (1928) Über die in der Diagnostik verabfolgten Dosen in R-Einheiten. Fortschritte auf dem Geb Röntgenstrahlen 38:385–398

43. Klövekorn GH, Profitlich P (1928) Ueber die bei Durchleuchtungen und Aufnahmen verabfolgten Röntgenstrahlenmengen. DMW - Dtsch Med Wochenschr 54:919–920. https://doi.org/10.1055/s-0028-1125326

44. Neeff, Th.C. (1930) Toleranzdosen in der Röntgendiagnostik. Fortschritte auf dem Geb Röntgenstrahlen 41:414–426

45. Schall WE (1932) X Rays: their origin, dosage and practical application, Fourth Edition. John Wright & Sons LTD, Stone Bridge, Bristol

46. Witte, E. (1933) Welche Mittel gibt es, um bei diagnostischer Verwendung der Röntgenstrahlen die Dosis zu vermindern? Fortschritte auf dem Geb Röntgenstrahlen 47:312–321

47. Jäderholm, K.B. (1935) Einige Untersuchungen über die Hautbelastung bei Röntgendiagnostik. Acta Radiol 16:518–527

48. Zimmer, K.G. (1935) Über Dosismessungen während Röntgendiagnostik. Fortschritte auf dem Geb Röntgenstrahlen 51:418–421

49. Turnbull, A, Leddy, E.T. (1935) A method for determining the limits of safety in roentgenography. Am J Roentgenol 34:258–260

50. Stehr, L. (1937) Strahlenschädigungen. Röntgenprax Diagn Röntgen- Radium- Lichttherapie 9:695–704

51. Ardran GM (1956) The Dose to Operator and Patient in X-ray Diagnostic Procedures. Br J Radiol 29:266–269. https://doi.org/10.1259/0007-1285-29-341-266

52. Lemmel, G. (1938) Strahlengefährdung des Kranken und des Arztes in der Röntgendiagnostik: Dosismessungen mit dem neuen Integral-Momentan-Dosismesser der Siemens-Reiniger -Werke. Fortschritte auf dem Geb Röntgenstrahlen 58:240–248

53. Smedal, M.I. (1942) Röntgen-ray dosage during routine diagnostic studies. Am J Roentgenol 48:807–815

54. Glasser O (1944) Medical Physics. The Year Book Publishers Inc., Chicago

55. Philips (1946) Philips Belichtingstabel 1946

56. Martin JH (1947) Radiation Doses Received by the Skin of a Patient during Routine Diagnostic X-ray Examinations. Br J Radiol 20:279–283. https://doi.org/10.1259/0007-1285-20-235-279

57. Ardran GM, Crooks HE (1953) A Comparison of Radiographic Techniques with Special Reference to Dosage. Br J Radiol 26:352–357. https://doi.org/10.1259/0007-1285-26-307-352

58. Sorrentino, J., Yalow, R. (1950) A Nomogram for Dose Determinations in Diagnostic Roentgenology. Radiology 55:748–753

59. Kirsch (1955) Radiation dangers in diagnostic radiology. J Am Med Assoc 158:1420–1423

60. Handloser, J.S., Love, R.A. (1951) Radiation Doses from Diagnostic X-Ray Studies. Radiology 57:252–254

61. Wachsmann, F. (1951) Dosisbelastung des Patienten bei röntgendiagnostischen Untersuchungen. Fortschritte auf dem Geb Röntgenstrahlen 75:728–733

62. Ritter VW, Warren SR, Pendergrass EP (1952) Roentgen Doses During Diagnostic Procedures. Radiology 59:238–251. https://doi.org/10.1148/59.2.238

63. Trout DE, Kelly JP, Cathey GA (1952) The use of filters to control radiation exposure to the patient in diagnostic roentgenology. Am J Roentgenol 67:946–963

64. van der Plaats, G.J. (1953) Medische Röntgendiagnostiek, First Edition. Tijdstroom, Lochem

65. Stanford, R.W., Vance, J. (1955) The quantity of radiation received by the reproductive organs of patients during routine diagnostic X-ray examinations. Br J Radiol 28:266–272

66. Ardran GM, Crooks HE (1957) Gonad Radiation Dose from Diagnostic Procedures. Br J Radiol 30:295–297. https://doi.org/10.1259/0007-1285-30-354-295

67. Billings MS, Norman A, Greenfield MA (1957) Gonad Dose During Routine Roentgenography. Radiology 69:37–41. https://doi.org/10.1148/69.1.37

68. Janker, H.C. (1958) Röntgen-Aufnahmetechnik. Teil I: Einstellungen. Johann Ambrosius Barth, München

69. Lincoln, A., Gupton, E.D. (1958) Radiation dose to gonads from diagnostic x-ray exposure. J Am Med Assoc 166:233–239

70. Sanders, A.P., Sharpe K, Cahoon JB, et al (1960) Radiation dose to the skin in Roentgen diagnostic procedures: optimum kVp, and tissue measurement techniques. Am J Roentgenol 84:359–368

71. Stahl, W., Sullivan RR, Wagstaff DG (1960) Findings of a Survey of X-Ray Units. Public Health Rep 75:652–658

72. Höfling, O. (1961) Strahlengefarhr und Strahlenschutz. In: Mathematisch-Naturwissenschaftliche Taschenbücher. Ferd. Dümmlers Verlag, Bonn, pp 172–179

73. Burr, R.C. (1962) Protection in the Use of Diagnostic Radiation. Can Med Assoc J 86:527–531

74. Spalding CK, Cowing RF (1964) A Survey of Exposures Received by Hospitalized Patients During Routine Diagnostic X-Ray Procedures. Radiology 82:113–119. https://doi.org/10.1148/82.1.113

75. Janker, H.C. (1964) Die Bestimmung von Röntgen-Belichtungsdatenund einfache Hilfsmittel dazu (Meßgerät und Röntgen-Belichtungsscheibe). Röntgenpraxis 17:13–21

76. van der Plaats, G.J. (1966) Medische Röntgendiagnostiek, Third Edition. N.V. Uitgeversmaatschappij de Tijdstroom, Lochem

77. Heller MB, Terdiman JF, Pasternack BS (1966) A procedure for calculation of gonadal x-ray dose in diagnostic radiography. Br J Radiol 39:686–692. https://doi.org/10.1259/0007-1285-39-465-686

78. Reekie D, Davison M, Davidson JK (1967) The radiation hazard in radiography of the female abdomen and pelvis. Br J Radiol 40:849–854. https://doi.org/10.1259/0007-1285-40-479-849

79. Rogers, R.T. (1969) Radiation dose to the skin in diagnostic radiography. Br J Radiol 42:511–518

80. Antoku S, Russell WJ, Milton RC, et al (1972) Dose to patients from Roentgenography. Health Phys 23:291-299.

81. Unscear (1972) Volume II: Scientific Annexes; Annex B. Doses from medical irradiation. In: UNSCEAR 1972 Report: Ionizing radiation: Levels and Effects. United Nations Publications

82. Matsunaga Y, Kawaguchi A, Kobayashi K, et al (2017) Patient exposure during plain radiography and mammography in Japan in 1974-2014. Radiat Prot Dosimetry 176:347–353. https://doi.org/10.1093/rpd/ncx017

83. ICRP (1982) ICRP 34. Protection of the Patient in Diagnostic Radiology. ICRP Publication 34. Pergamon Press, Oxford

84. van der Plaats, G.J., Vijlbrief P (1978) Medische Röntgentechniek in de Diagnostiek, Fifth Edition. Uitgeversmaatschappij De Tijdstroom B.V., Lochem

85. Bengtsson G, Blomgren P-G, Bergman K, Åberg L (1978) Patient Exposures and Radiation Risks In Swedish Diagnostic Radiology. Acta Radiol Oncol Radiat Phys Biol 17:81–105. https://doi.org/10.3109/02841867809127910

86. Buch J, Duftschmid KE (1980) Strahlenbelastung des Arztes und des Patienten in der Unfallchirurgie. Band XX. In: Strahlenschutz in Forschung und Praxis. Georg Thieme Verlag, Stuttgart

87. Stieve, F.E. (1982) Strahlenexposition von Patienten bei röntgendiagnostischen Maßnahmen. Strahlenschutz Forsch Prax 23:37–78

88. Padikal TN, Fivozinsky SP (1982) Medical Physics Data Book. U.S. Department of Commerce, Washington, D.C

89. Drexler G, Panzer W, Widenmann L, Zankl M (1985) Die Bestimmung von Organdosen in der Röntgendiagnostik. Verlag H. Hoffmann, Berlin

90. Shrimpton PC, Wall BF, Jones DG, et al (1986) Doses to patients from routine diagnostic X-ray examinations in England. Br J Radiol 59:749–758. https://doi.org/10.1259/0007-1285-59-704-749

91. Zamenhof, R.G., Shahabi, S., Morgan, H. T. (1987) An Improved Method for Estimating the Entrance Exposure in Diagnostic Radiographic Examinations. Am J Roentgenol 149:631–637

92. Padovani, R., Contento G, Fabretto M, et al (1987) Patient doses and risks from diagnostic radiology in North-East Italy. Br J Radiol 60:155–165

93. Gallini, R.R., Belletti, S., Berna, V., Giugni, U. (1992) Adult and child doses in standardised x ray examinations. Radiat Prot Dosimetry 43:41–47

94. Siemens (1994) Siemens-Basisbelichtungstabelle. In: Medizinische Technik: Daten, Formeln, Fakten. Siemens, pp 44–45

95. Hart D, Hillier MC, Wall BF (2002) Doses to patients from medical X-ray examinations in the U.K.: 2000 review. National Radiological Protection Board, Chilton, Didcot, Oxfordshire

96. Rannikko S, Karila KTK, Toivonen M (1997) Patient and population doses of X-ray diagnostics in Finland. Radiation and Nuclear Safety Authority

97. Persliden J, Beckman K-W, Geijer H, Andersson T (2002) Dose-image optimisation in digital radiology with a direct digital detector: an example applied to pelvic examinations. Eur Radiol 12:1584–1588. https://doi.org/10.1007/s00330-001-1206-0

98. Ciraj, O., Markovic, S., Kosutic, D. (2004) Patient dose from conventional diagnostic radiology procedures in Serbia and Montenegro. J Prev Med 12:26–34

99. Hart D, Hillier MC, Wall BF (2007) Doses to patients from radiographic and fluoroscopic X-ray imaging procedures in the UK - 2005 review. Health Protection Agency, Radiation Protection Division, Didcot, Oxfordshire

100. Compagnone G, Pagan L, Bergamini C (2005) Effective dose calculations in conventional diagnostic X-ray examinations for adult and paediatric patients in a large Italian hospital. Radiat Prot Dosimetry 114:164–167. https://doi.org/10.1093/rpd/nch508

101. Schuncke A, Neitzel U (2005) Retrospective patient dose analysis of a digital radiography system in routine clinical use. Radiat Prot Dosimetry 114:131–134. https://doi.org/10.1093/rpd/nch535

102. Aldrich, J.E., Duran, E., Dunlop, P., Mayo, J.R. (2006) Optimization of Dose and Image Quality for Computed Radiography and Digital Radiography. J Digit Imaging 19:126–131

103. Toosi M.T.B, M. Nazery, H. Zare (2006) Application of dose-area product compared with three other dosimetric quantities used to estimate patient effective dose in diagnostic radiology. Iran J Radiat Res 21–27

104. Kloth JK, Rickert M, Gotterbarm T, et al (2015) Pelvic X-ray examinations in follow-up of hip arthroplasty or femoral osteosynthesis – Dose reduction and quality criteria. Eur J Radiol 84:915–920. https://doi.org/10.1016/j.ejrad.2015.02.001

105. Tsapaki V, Tsalafoutas IA, Chinofoti I, et al (2007) Radiation doses to patients undergoing standard radiographic examinations: a comparison between two methods. Br J Radiol 80:107–112. https://doi.org/10.1259/bjr/87150291

106. Compagnone G, Pagan L, Baleni MC, et al (2008) Patient dose in digital projection radiography. Radiat Prot Dosimetry 129:135–137. https://doi.org/10.1093/rpd/ncn013

107. Hart D, Hillier MC, Shrimpton PC, et al (2012) Doses to patients from radiographic and fluoroscopic X-ray imaging procedures in the UK: 2010 review. Health Protection Agency, Didcot, Oxfordshire

108. Tonkopi E, Daniels C, Gale MJ, et al (2012) Local Diagnostic Reference Levels for Typical Radiographic Procedures. Can Assoc Radiol J 63:237–241. https://doi.org/10.1016/j.carj.2011.02.004

109. Osei EK, Darko J (2013) A Survey of Organ Equivalent and Effective Doses from Diagnostic Radiology Procedures. ISRN Radiol 2013:1–9. https://doi.org/10.5402/2013/204346

110. Ofori K, Gordon SW, Akrobortu E, et al (2014) Estimation of adult patient doses for selected X-ray diagnostic examinations. J Radiat Res Appl Sci 7:459–462. https://doi.org/10.1016/j.jrras.2014.08.003

111. de Vries G, Bijwaard H (2015) A comparison of radiation doses of radiology procedures to Diagnostic Reference Levels (DRLs) in Dutch hospitals. In: ESR - Electronic Presentation Online System. European Society of Radiology, Vienna, pp 1–11

112. Elshiekh E, Suliman II, Habbani F (2015) Performance evaluation of two computed radiography systems and patient dose in pelvic examination. Radiat Prot Dosimetry 165:392–396. https://doi.org/10.1093/rpd/ncv125

113. England, A., Evans P, Harding L, et al (2015) Increasing source-to-image distance to reduce radiation dose from digital radiography pelvic examinations. Radiol Technol 86:246–256

114. Jacobs, S.J., Kuhl L., Xu G, et al (2015) Optimum tube voltage for pelvic direct radiography: a phantom study. South Afr Radiogr 53:15–19

115. Aliasgharzadeh A, Mihandoost E, Masoumbeigi M, et al (2015) Measurement of Entrance Skin Dose and Calculation of Effective Dose for Common Diagnostic X-Ray Examinations in Kashan, Iran. Glob J Health Sci 7:. https://doi.org/10.5539/gjhs.v7n5p202

116. Asada Y, Suzuki S, Minami K, et al (2016) Survey of patient exposure from general radiography and mammography in Japan in 2014. J Radiol Prot 36:N8–N18. https://doi.org/10.1088/0952-4746/36/2/N8

117. Khoshdel-Navi D, Shabestani-Monfared A, Deevband MR, et al (2016) Local-Reference Patient Dose Evaluation in Conventional Radiography Examinations in Mazandaran, Iran. J Biomed Phys Eng 6:61–70

118. Heilmaier C, Zuber N, Weishaupt D (2017) Implementation of a patient dose monitoring system in conventional digital X-ray imaging: initial experiences. Eur Radiol 27:1021–1031. https://doi.org/10.1007/s00330-016-4390-7

119. Yacoob HY, Mohammed HA (2017) Assessment of patients X-ray doses at three government hospitals in Duhok city lacking requirements of effective quality control. J Radiat Res Appl Sci 10:183–187. https://doi.org/10.1016/j.jrras.2017.04.005

120. Jeukens CRLPN (2018) Dose adult anteroposterior pelvic radiograph. MUMC+, Maastricht (personal communication)
